# Health impact, budget impact, and price threshold for cost-effectiveness of lenacapavir for HIV pre-exposure prophylaxis in eastern and southern Africa: a modelling analysis

**DOI:** 10.1016/S2352-3018(24)00239-X

**Published:** 2024-09-20

**Authors:** Linxuan Wu, David Kaftan, Rachel Wittenauer, Cory Arrouzet, Nishali Patel, Arden L Saravis, Brian Pfau, Edinah Mudimu, Anna Bershteyn, Monisha Sharma

**Affiliations:** a Department of Epidemiology, University of Washington, Seattle, WA, USA; b Department of Global Health, University of Washington, Seattle, WA, USA; c Department of Health Metrics Sciences, University of Washington, Seattle, WA, USA; d Department of Population Health, New York University Grossman School of Medicine, New York, NY, USA; e CHOICE Institute, University of Washington School of Pharmacy, Seattle, WA, USA; f Department of Decision Sciences, University of South Africa, Pretoria, South Africa

## Abstract

**Background:**

Injectable lenacapavir administered every 6 months is a promising product for HIV pre-exposure prophylaxis (PrEP). We aimed to estimate the health and budget impacts and threshold price at which lenacapavir could be cost-effective in eastern and southern Africa.

**Methods:**

We adapted an agent-based network model, EMOD-HIV, to simulate lenacapavir scale-up in Zimbabwe, South Africa, and western Kenya from 2026 to 2035. Uptake assumptions were informed by a literature review of PrEP product preferences. In the main analysis, we varied lenacapavir coverage by subgroup: female sex workers (40% coverage); male clients of female sex workers (40%); adolescent girls and young women aged 15–24 years with more than one sexual partner (32%); women aged 25 years and older with more than one sexual partner (36%); and males with more than one sexual partner (32%). We also assessed a higher coverage scenario (64–76% across subgroups) and scenarios of expanding lenacapavir use, varying from concentrated among those at highest HIV risk to broader coverage including those at medium HIV risk. We estimated the maximum per-dose lenacapavir price that achieved cost-effectiveness (<US$500 per disability-adjusted life-year averted), infections averted, and 5-year budget impact, compared with daily oral PrEP only.

**Findings:**

In the main analysis, lenacapavir was projected to achieve from 1·6% (95% uncertainty interval [UI] 1·5–1·8) to 4·0% (3·4–5·1) population coverage across settings and to avert from 12·3% (5·4–19·5) to 18·0% (11·0–22·9) of infections over 10 years. The maximum price per dose was highest in South Africa ($106·28 [95% UI 95·72–115·87]), followed by Zimbabwe ($21·15 [17·70–24·89]), and lowest in western Kenya ($16·58 [15·44–17·70]). The 5-year budget impact was US$507·25 million (95% UI 436·14–585·42) in South Africa, $16·80 million (13·95–22·64) in Zimbabwe, and $4·09 million (3·86–4·30) in western Kenya. In the higher coverage scenario, lenacapavir distribution was projected to reach from 3·2% (95% UI 2·9–3·6) to 8·1% (6·8–10·5) population coverage and to avert from 21·2% (95% UI 14·7–18·5) to 33·3% (28·5–36·9) of HIV infections across settings over 10 years. Price thresholds were lower than in the main analysis: $88·34 (95% UI 83·02–94·19) in South Africa, $17·71 (15·61–20·05) in Zimbabwe, and $14·78 (14·33–15·30) in western Kenya. The 5-year budget impact was higher than the main analysis: $835·29 million (95% UI 736·98–962·98) in South Africa, $29·50 million (24·62–39·52) in Zimbabwe, and $7·45 million (7·11–7·85) in western Kenya. Expanding lenacapavir coverage resulted in higher HIV infections averted but lower price thresholds than scenarios of concentrated use among those with highest HIV risk.

**Interpretation:**

Our findings suggest that lenacapavir could avert substantial HIV incidence and that price thresholds and budget impacts vary by setting and coverage. These results could inform policy deliberations regarding lenacapavir pricing and resource planning.

**Funding:**

The Bill & Melinda Gates Foundation.

## Introduction

HIV incidence remains unacceptably high in eastern and southern Africa, causing substantial morbidity, mortality, and financial strain on health systems. Daily oral pre-exposure prophylaxis (PrEP) is effective at preventing HIV acquisition and is available in clinics in the region, but uptake is far below UNAIDS targets^1^ and adherence and persistence are suboptimal. Barriers to oral PrEP use include pill burden and stigma associated with taking daily antiretrovirals.^2^ Studies among key groups and the general population indicate a preference for long-acting PrEP compared with daily oral pills, with injectables preferred over implants and a strong desire for longer duration products.^2^ Long-acting PrEP can overcome barriers associated with uptake, adherence, and persistence by providing more convenient and discreet alternatives to daily pill taking and mitigating adherence challenges.

**Research in context**

**Evidence before this study**

We searched PubMed for modelling studies published from July 31, 2014, to July 31, 2024, that assessed the health or economic impact of long-acting pre-exposure prophylaxis (PrEP) scale-up in Africa using the terms: “HIV” AND “lenacapavir” OR “pre-exposure prophylaxis” OR “PrEP” AND (a list of terms indicating health impact), “cost*”, “budget impact”, “economic evaluation” AND (a list of countries in sub-Saharan Africa), “sub-Saharan” AND “model*”, OR “mathematical model*” without language restrictions. We did not find any modelling studies evaluating the maximum cost or budget impact of lenacapavir for PrEP. However, several modelling studies evaluated the cost-effectiveness of other long-acting injectable PrEP products in South Africa. Two studies found that long-acting cabotegravir was cost-effective when used by populations at substantial risk of HIV, and another found it was not cost-effective when delivered to heterosexual men. Five more recent modelling studies (four parameterised to South Africa and one to sub-Saharan Africa) found that long-acting cabotegravir was not cost-effective when targeted to those at highest risk of HIV unless the price was reduced. One compartment model evaluated the price threshold at which long-acting cabotegravir would be similarly cost-effective to oral PrEP in South Africa and found a per-dose cost ranging from US$9·05 to $14·47. We found no studies evaluating long-acting PrEP in African settings other than South Africa, which has a considerably higher gross domestic product than other countries in the region.

**Added value of this study**

We evaluated the health impact, budget impact, and maximum price threshold of lenacapavir in three African settings (South Africa, Zimbabwe, and western Kenya), which were early adopters of oral PrEP. We found that lenacapavir scale-up to 1·6–4·0% population coverage across settings could avert 12·3–18·0% of infections and could be cost-effectively implemented at a price per dose of US$106·28 (South Africa), $21·15 (Zimbabwe), and $16·58 (western Kenya) in our main analysis based on PrEP preference data. Price threshold, volume of doses needed, and budget impact varied by setting and coverage.

**Implications of all the available evidence**

Long-acting PrEP formulations have the potential to substantially reduce HIV incidence in low-income and middle-income countries, but costs will likely need to be reduced to enable equitable implementation. Our findings could inform price negotiations and public health planning regarding adoption of novel PrEP products.

Lenacapavir has emerged as a particularly promising long-acting product for HIV prevention, with interim phase 3 clinical trial results showing 100% efficacy in females aged 16–25 years in Brazil, Peru, South Africa, and the USA.^3^ Lenacapavir is an antiviral that disrupts the HIV-1 capsid, interfering with several stages of HIV replication.^4^ Administered through twice yearly subcutaneous injections, lenacapavir has an extended half-life, which provides 6 months of HIV protection.^4^ As a first-in-class antiretroviral, lenacapavir is not expected to have overlapping drug resistance mutations with other antiretrovirals and has a favourable safety profile, suggesting feasibility for widespread use.^4^

Although lenacapavir has the potential to greatly expand PrEP coverage in eastern and southern Africa, its effect is contingent on its affordability, particularly in resource constrained settings disproportionately impacted by HIV. Evaluating realistic scenarios of lenacapavir uptake is crucial for informing price thresholds that enable cost-effective implementation, estimating product volume needed, and projecting health impact. We sought to estimate the health and economic effects of lenacapavir scale-up in South Africa, western Kenya, and Zimbabwe, countries that were early adopters of oral PrEP, suggesting favourable regulatory environments for lenacapavir adoption. As the per-dose price of lenacapavir is uncertain, we estimated the maximum price that achieves cost-effectiveness in each setting.

## Methods

### Mathematical model

We augmented an agent-based network model previously developed by the Institute for Disease Modeling.^5^, ^6^ EMOD-HIV is an open-source microsimulation model integrating population demography, HIV disease progression, and heterosexual network-based transmission of HIV, designed to match age and sex specific propensities of sexual partnership formation. The model simulates HIV transmission and the effect of HIV treatment and prevention interventions on the epidemic. HIV interventions are incorporated via configurable health-care modules, including an HIV care continuum with HIV testing, linkage, and retention on antiretroviral therapy (ART), and a PrEP continuum with uptake, adherence, persistence, and re-engagement. The model tracks outcomes including HIV infections, HIV-related deaths, and health-care utilisation, using monthly time steps to enable calculation of disability-adjusted life-years (DALYs) and health-related costs.

The model was parameterised using epidemiological data from South Africa (adult HIV prevalence in 2020, 19·1%), western Kenya (Homa Bay, Kisii, Kisumu, Migori, Nyamira, and Siaya counties; adult HIV prevalence in 2020, 11·3%), and Zimbabwe (adult HIV prevalence in 2020, 11·9%), including age-specific fertility, mortality, voluntary male circumcision coverage, number of people on oral PrEP, health-care-seeking behaviour, population size and age and sex structure, and sizes of key populations including female sex workers and male clients of female sex workers.^7^, ^8^ We calibrated the model to primary data for HIV prevalence by age and sex and number of people on ART using an optimisation algorithm that maximises the likelihood of matching observed data. We selected 100 good-fitting model parameter sets using roulette resampling in proportion to the goodness-of-fit to calibration data. Parameterisation and calibration data are in the appendix (pp 11–60).

### Modelled scenarios

In the baseline (no lenacapavir) scenario, we assumed availability of only daily oral PrEP, which was used by both males and females aged 16–49 years with HIV risk indication and scaled to currently observed levels in each country.^9^ We assumed oral PrEP decreased HIV acquisition risk by 75% based on clinical trials accounting for average adherence and assumed a 3-month persistence based on real-world implementation.^10^ In intervention scenarios, lenacapavir was introduced in 2026 and scaled linearly to target coverage by 2029. We assumed lenacapavir implementation ends in 2035 to evaluate the effect of a 10-year delivery commitment and used a 35-year analytic time horizon (2026–60) to capture long-term outcomes and estimate the price threshold of lenacapavir. For the main analysis, we estimated realistic lenacapavir uptake using a comprehensive literature review of PrEP preferences in eastern and southern Africa among different population subgroups at risk of HIV and evaluation of health-care accessibility across groups (appendix pp 4–10). Based on our review, we developed the following coverage estimates: 40% for female sex workers; 40% for male clients of female sex workers; 32% for adolescent girls and young women aged 15–24 years with more than one sexual partner; 36% for women aged 25 years and older with more than one sexual partner; and 32% for men aged 18 years and older with more than one sexual partner. We assumed lenacapavir decreased HIV acquisition risk among both sexes by 95% for 6 months.^2^ People who discontinued lenacapavir were eligible to re-initiate if they met eligibility criteria. Leveraging insights from family planning literature, which shows an increase in total contraceptive use with introduction of new methods, we assumed oral PrEP uptake would remain at currently observed levels and the effect of lenacapavir would be largely additive.^11^ This assumption aligns with qualitative findings on PrEP preferences suggesting that some portion of the population would still choose oral PrEP despite availability of long-acting products.^2^

### Sensitivity analysis

Because lenacapavir clinical trial results are not yet available for males, we evaluated a scenario of lower lenacapavir effectiveness (80%) among males while maintaining 95% effectiveness in females. Additionally, using the setting of South Africa, we assessed two scenarios varying background oral PrEP adherence to 58% and 95% in both the baseline and intervention simulations, and a third scenario in which background daily oral PrEP was scaled up to three times currently observed levels by 2026 in both the baseline and intervention simulations. We also conducted one-way sensitivity analyses of our main analysis, varying costs of lenacapavir provision, ART, lenacapavir demand generation, and wastage. We additionally evaluated the budget impact of a US$50 per-dose price, which has been estimated as a potential near-term price point for generic drugs as production scales up.^12^

Due to the uncertainty surrounding product volume and populations reached by lenacapavir scale-up, we conducted extensive sensitivity analyses varying lenacapavir coverage. We assessed a higher uptake scenario informed by the upper bound estimates of our PrEP preferences literature review, assuming the following uptake across subgroups: 72% for female sex workers; 72% for male clients of female sex workers; 76% for adolescent girls and young women aged 15–24 years with more than one sexual partner; 72% for women aged 25 years and older with more than one sexual partner; and 64% for men age 18 years and older with more than one sexual partner. We also conducted a set of sensitivity analyses varying distribution of lenacapavir to females with expanding degrees of HIV risk (with and without coverage among males). To categorise HIV risk in the model, we modified an empirically validated risk scoring tool (Vaginal and Oral Interventions to Control the Epidemic [VOICE] score) developed to identify African females with a high likelihood of HIV acquisition based on demographic, behavioural, and partnership-level factors.^13^ We evaluated the following scenarios from highest to lowest HIV risk (with higher VOICE score indicating greater risk): (1) female sex workers; (2) a VOICE score of 5 or more (defined as three of the following factors: age 15–24 years, unmarried, one or more male sexual partner who has other partners, and medium sexual activity category); (3) a VOICE score of 3 or more (defined as sexually active with at least two of the previously mentioned factors); and (4) a VOICE score of 1 or more (defined as sexually active with at least one of the previously mentioned factors). Each subsequent scenario is inclusive of the previous ones. All scenarios were evaluated alone and in combination with males who are clients of female sex workers or males with more than one sexual partner (appendix p 58).

### Model outcomes

For intervention scenarios, we estimated number of HIV infections, HIV-related deaths, DALYs, and percentage of each outcome averted compared with the counterfactual scenario of oral PrEP only. We estimated lenacapavir coverage, total doses of lenacapavir distributed over the first 5 years, and infections averted per 1000 doses. We calculated 95% uncertainty intervals across 100 parameter sets to assess parameter uncertainty. Analysis of model outputs was performed using R version 4.2.2.

### Price threshold and budget impact analysis

Using the payer perspective, we calculated the maximum price per dose for lenacapavir to achieve cost-effectiveness per scenario using a commonly referenced supply-side cost-effectiveness threshold of US$500 per DALY averted.^14^ Costs (in 2021 $US) included HIV testing, ART, HIV-related hospitalisations, and costs related to lenacapavir provision including personnel, consumables, product wastage, and demand generation activities (assumed to be 10% of the per-dose price; table 1 and appendix pp 54–56). We additionally evaluated a lower cost threshold of $200 per DALY averted for all settings and a higher threshold of $1175 per DALY averted for South Africa based on country-specific estimates, which are higher in South Africa than in the rest of eastern and southern Africa.^14^

**Table 1:** Model parameters with their assumptions and cost inputs

		**Value**	**Source**
Oral PrEP effectiveness		75%	Baeten et al (2012)^10^
Lenacapavir effectiveness		95%	Bekker et al (2024)^15^
Lenacapavir scale-up period		2026–29	Assumption
Lenacapavir implementation period		2026–35	Assumption
Analytic time horizon		2026–60	Assumption
Costs			
	Lenacapavir demand generation	10% per dose	Assumption based on VMMC literature
	Lenacapavir provision cost	$8·55	Mangale et al (2022)^16^ and UNICEF Supply Division^17^
	Lenacapavir wastage	5%	Assumption
Western Kenya annual health-care costs (among those not on ART)			
	HIV-positive CD4 <200 cells per μL	$110·30	Eaton et al (2014)^18^
	HIV-positive CD4 200–349 cells per μL	$30·38	Eaton et al (2014)^18^
	HIV-positive CD4 >350 cells per μL	$8·59	Eaton et al (2014)^18^
	End-of-life care	$105·68	Eaton et al (2014)^18^
	Annual ART provision	$196·85	Larson et al (2018),^19^ Long et al (2010),^20^ The Global Fund,^21^ and Haas et al (2015)^22^
	Oral PrEP per person-month	$10·88	Wanga et al (2021)^23^
	Facility-based HIV-positive test	$3·68	Meisner et al (2021)^24^
	Facility-based HIV-negative test	$2·64	Meisner et al (2021)^24^
South Africa annual health-care costs (among those not on ART)			
	HIV-positive CD4 <200 cells per μL	$374·08	Eaton et al (2014)^18^
	HIV-positive CD4 200–349 cells per μL	$102·95	Eaton et al (2014)^18^
	HIV-positive CD4 >350 cells per μL	$29·10	Eaton et al (2014)^18^
	End-of-life care	$358·10	Eaton et al (2014)^18^
	Annual ART provision	$189·56	Larson et al (2018),^19^ Long et al (2010),^20^ The Global Fund,^21^ and Haas et al (2015)^22^
	Oral PrEP per person-month	$15·20	Jamieson et al (2022)^25^
	Facility-based HIV-positive test	$5·62	Meyer-Rath et al (2019)^26^
	Facility-based HIV-negative test	$3·62	Meyer-Rath et al (2019)^26^
Zimbabwe annual health-care costs (among those not on ART)			
	HIV-positive CD4 <200 cells per μL	$93·98	Eaton et al (2014)^18^
	HIV-positive CD4 200–349 cells per μL	$25·89	Eaton et al (2014)^18^
	HIV-positive CD4 >350 cells per μL	$7·32	Eaton et al (2014)^18^
	End-of-life care	$89·83	Eaton et al (2014)^18^
	Annual ART provision	$176·01	Larson et al (2018),^19^ Long et al (2010),^20^ The Global Fund,^21^ and Haas et al (2015)^22^
	Oral PrEP per person-month	$9·25	Wanga et al (2021)^23^
	Facility-based HIV-positive test	$3·13	Meisner et al (2021)^24^
	Facility-based HIV-negative test	$2·24	Meisner et al (2021)^24^

Costs are adjusted for inflation and gross domestic product per capita ratio where applicable. All costs are in 2021 US$. ART costs are of delivery costs and assume 3% of patients receive second-line ART. Lenacapavir demand generation costs are adapted from the literature for VMMC demand generation. Additional details on costing methods are in the appendix (pp 55–57). ART=antiretroviral therapy. PrEP=pre-exposure prophylaxis. VMMC=voluntary medical male circumcision.

Using the per-dose price threshold calculated in each scenario as well as delivery and programme costs, we estimated the undiscounted 5-year budget impact of lenacapavir scale-up from the health-care payer perspective by cost category.

### Role of the funding source

The funder of the study had no role in study design, data collection, data analysis, data interpretation, or writing of the report.

## Results

In the main scenario, lenacapavir scale-up was estimated to avert 18·0% (95% uncertainty interval [UI] 11·0–22·9) of HIV infections in western Kenya, 17·0% (3·3–28·2) in Zimbabwe, and 12·3% (5·4–19·5) in South Africa during 10 years of implementation compared with the reference scenario of oral PrEP only (table 2). Although the proportion of infections and deaths averted was lowest in South Africa, the absolute number of DALYs averted was greatest in South Africa due to higher HIV prevalence and population size (table 2). Population-level lenacapavir coverage was 1·6% (1·5–1·8) in South Africa, 2·9% (2·8–3·0) in western Kenya, and 4·0% (3·4–5·1) in Zimbabwe, with number of doses required over the first 5 years of implementation highest in South Africa, followed by Zimbabwe, and western Kenya (table 2). Using a threshold of US$500 per DALY averted, the maximum per-dose price to achieve cost-effectiveness was $106·28 (95% UI 95·72–115·87) in South Africa, $21·15 (17·70–24·89) in Zimbabwe, and $16·58 (15·44–17·70) in western Kenya. Under a lower threshold of $200 per DALY averted, per-dose prices decreased to $74·87 (66·65–82·33) in South Africa, $11·68 (9·09–14·21) in Zimbabwe, and $11·32 (10·34–12·23) in western Kenya. Assuming a higher cost threshold ($1175 per DALY averted for South Africa only) resulted in higher maximum per-dose prices: $177·09 (155·81–196·12) for the baseline coverage and $146·67 (135·80–158·56) for the higher coverage scenario.

**Table 2:** Health and budget impact and maximum price threshold for lenacapavir scale-up

	**Main scenario**			**Higher lenacapavir coverage scenario**		
	Western Kenya	Zimbabwe	South Africa	Western Kenya	Zimbabwe	South Africa
Lenacapavir coverage	2·9% (2·8 to 3·0)	4·0% (3·4 to 5·1)	1·6% (1·5 to 1·8)	5·8% (5·6 to 6·0)	8·1% (6·8 to 10·5)	3·2% (2·9 to 3·6)
HIV infections averted	18·0% (11·0 to 22·9)	17·0% (3·3 to 28·2)	12·3% (5·4 to 19·5)	33·3% (28·5 to 36·9)	31·0% (21·3 to 45·8)	21·2% (14·7 to 28·5)
HIV-associated deaths averted	3·5% (0·1 to 6·6)	5·8 (−3·2 to 14·3)	3·0% (−0·7 to 6·8)	6·7% (3·1 to 10·1)	10·6% (1·6 to 19·3)	4·9% (0·8 to 8·8)
Doses required over the first 5 years	145 454 (139 757 to 151 137)	501 406 (421 531 to 645 223)	3 942 509 (3 541 901 to 4 408 477)	286 776 (275 591 to 298 169)	998 447 (833 636 to 1 309 415)	7 728 013 (6 915 810 to 8 677 953)
HIV infections averted per 1000 doses	29 (17 to 40)	34 (6 to 74)	28 (11 to 46)	27 (22 to 32)	31 (15 to 57)	24 (16 to 36)
DALYs averted	45 315 (73 231 to 22 453)	282 649 (−12 843 to 833 338)	1 324 790 (−79 790 to 2 908 432)	83 650 (113 843 to 60 873)	509 733 (1 044 646 to 135 655)	2 176 579 (4 055 428 to 697 331)
Maximum price per dose at $500 per DALY averted, $	16·58 (15·44 to 17·70)	21·15 (17·70 to 24·89)	106·28 (95·72 to 115·87)	14·78 (14·33 to 15·30)	17·71 (15·61 to 20·05)	88·34 (83·02 to 94·19)
Maximum price per dose at $200 per DALY averted, $	11·32 (10·34 to 12·23)	11·68 (9·09 to 14·21)	74·87 (66·65 to 82·33)	9·87 (9·38 to 10·31)	9·23 (7·73 to 10·77)	62·34 (58·18 to 66·88)
5-year budget impact, $	4 086 333 (3 863 089 to 4 297 703)	16 803 502 (13 951 046 to 22 639 690)	507 252 479 (436 138 529 to 585 420 059)	7 451 884 (7 110 730 to 7 845 199)	29 500 374 (24 615 177 to 39 523 839)	835 292 422 (736 980 200 to 962 975 488)
Modelled population size in 2026	4 299 746 (4 291 296 to 4 310 059)	10 683 464 (10 621 909 to 10 754 812)	41 792 955 (41 519 588 to 42 030 162)	4 299 746 (4 291 296 to 4 310 059)	10 683 464 (10 621 909 to 10 754 812)	41 792 955 (41 519 588 to 42 030 162)

Health impacts are compared to the baseline scenario of daily oral pre-exposure prophylaxis only. HIV infections averted are reported for people aged 15–65 years over 10 years of lenacapavir implementation; deaths averted are calculated over the 35-year time horizon. Lenacapavir coverage is reported for people aged 15–49 years over 10 years of lenacapavir implementation. Prices are in 2021 US$. Values in parentheses show 95% uncertainty intervals representing the 2·5th and 97·5th percentiles across 100 parameter sets. DALYs=disability-adjusted life-years.

**Figure 1:**
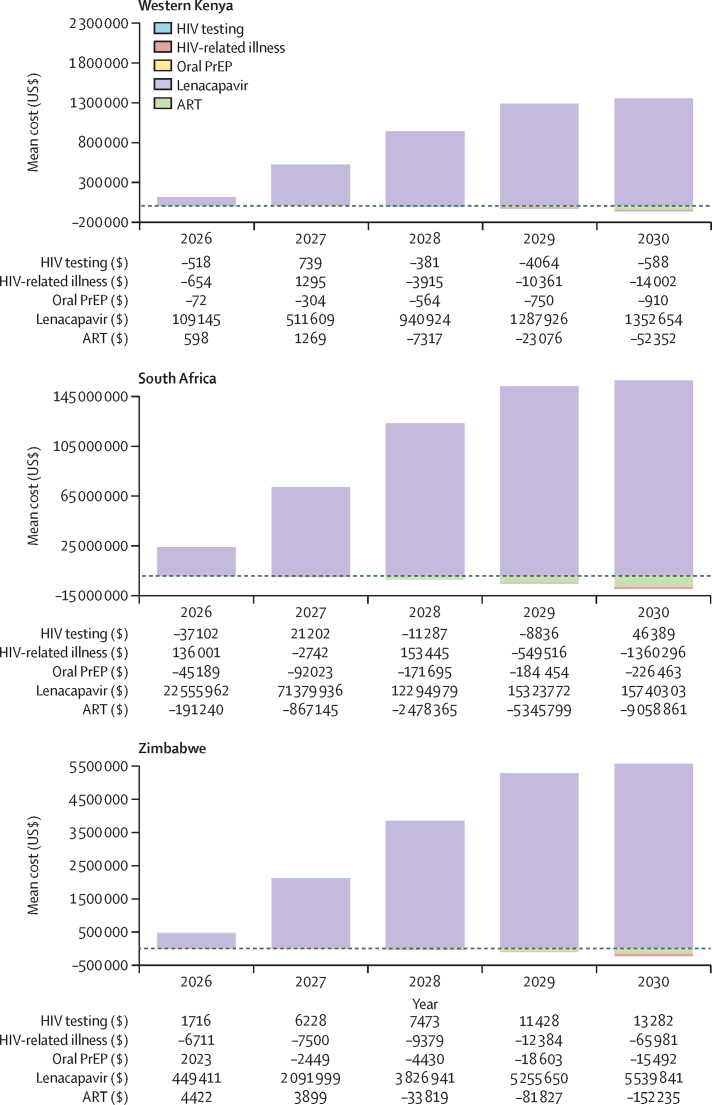
5-year budget impact analysis ART=antiretroviral therapy. PrEP=pre-exposure prophylaxis.

**Figure 2:**
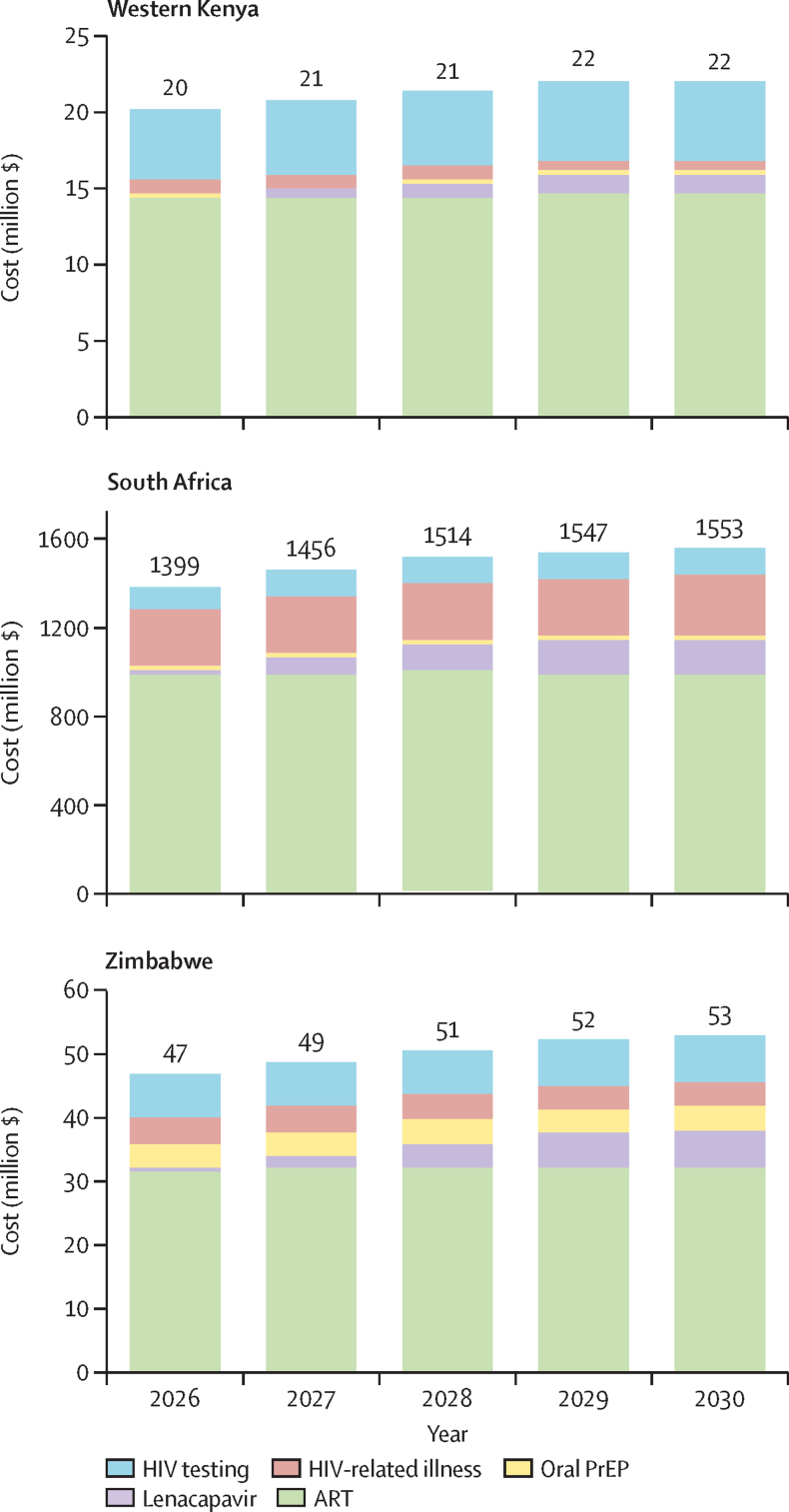
5-year total costs ART=antiretroviral therapy. PrEP=pre-exposure prophylaxis.

Using the per-dose price calculated in the main analysis (at the $500 threshold), the 5-year budget impact of lenacapavir implementation was $4 086 333 (95% UI 3 863 089–4 297 703) in western Kenya, $16 803 502 (13 951 046–22 639 690) in Zimbabwe, and $507 252 479 (436 138 529–585 420 059) in South Africa (table 2). Costs were highest in South Africa, which had the largest modelled population size and highest per-dose price. Costs increased with implementation year as lenacapavir scaled to targeted levels; for example, year 1 costs in Kenya were $108 500 (89 887–128 366) versus $1 284 802 (1 185 136–1 372 277) in year 5; more than 90% of the budget impact was due to lenacapavir provision (figure 1 and appendix p 65). Cost savings due to HIV-related illness and ART averted increased over time but accounted for less than 2% of costs across settings. Although lenacapavir provision made up the largest portion of the budget impact, the highest total costs were due to ART provision (approximately 70% of total; figure 2).

In sensitivity analyses evaluating a higher lenacapavir coverage scenario, lenacapavir distribution reached 3·2–8·1% population coverage across settings and averted 33·3% (95% UI 28·5–36·9) of HIV infections in western Kenya, 31·0% (21·3–45·8) in Zimbabwe, and 21·2% (14·7–28·5) in South Africa over 10 years (table 2). Both doses required and DALYs averted were nearly double that of the main scenario and the price threshold at $500 per DALY averted was 10–18% lower across settings: $88·34 (95% UI 83·02–94·19) in South Africa, $17·71 (15·61–20·05) in Zimbabwe, and $14·78 (14·33–15·30) in western Kenya. The budget impact followed a similar pattern as the main scenario, with lenacapavir provision accounting for most costs incurred (appendix pp 86–88). Cost savings from HIV-related illness and ART averted were higher but still represented a small proportion of the budget impact (5–8%; appendix pp 66–68).

The sensitivity analysis assuming higher oral PrEP availability (three times observed levels) did not substantially affect the maximum price threshold in South Africa, nor did varying oral PrEP effectiveness to 58% or 95% (appendix p 69). Assuming 80% lenacapavir effectiveness in men decreased HIV infections averted by 0·8–2·8% across settings and reduced price thresholds by 6·8–7·8% in western Kenya and South Africa and 16·8% in Zimbabwe (appendix p 70). In one-way sensitivity analyses, price thresholds were most sensitive to demand generation and ART costs; prices in Zimbabwe and western Kenya were additionally sensitive to lenacapavir provision costs (appendix p 92).

In sensitivity analyses evaluating lenacapavir distribution to varying subgroups, price thresholds were considerably higher for scenarios of lenacapavir for female sex workers only than other scenarios; however, HIV infections averted were lowest (table 3). HIV incidence reductions increased with expanding coverage to females with lower VOICE scores, but price thresholds decreased. In scenarios of lenacapavir provision to females with the highest HIV risk (eg, female sex workers and those with VOICE scores >5), adding males lowered the price threshold considerably (up to 50%), but price thresholds were similar when adding males to scenarios that included females with lower HIV risk (VOICE scores >3 and VOICE scores >1; table 3). In South Africa and Zimbabwe, including males was more efficient (in terms of price threshold and HIV infections averted) than expansion to the group of females with the next highest risk across VOICE scores (eg, for South Africa, the maximum price for females with VOICE score >5 and males with more than one sexual partner is $65·0 [95% UI 61·0–69·0] *vs* $43·0 [40·0–45·0] for females with VOICE >3 only). However, in western Kenya, the opposite trend was observed, with adding males being less efficient than expansion to the VOICE score across scenarios. Similar patterns were observed when evaluating the effect of adding male clients of female sex workers to scenarios of females only instead of males with more than one sexual partner (appendix pp 72–85). Additional health impacts, number of doses needed, and budget impact results are in the appendix (pp 71–85).

**Table 3:** HIV incidence reduction and maximum price per dose for increasing lenacapavir distribution scenarios

	**South Africa**		**Western Kenya**		**Zimbabwe**	
	Price threshold	HIV incidence reduction	Price threshold	HIV incidence reduction	Price threshold	HIV incidence reduction
**Female sex workers**						
Female sex workers only	$589·0 (454·0–726·0)	6·0% (1·0–14·0)	$23·0 (18·0–28·0)	7·0% (1·7–12·8)	$39·0 (28·0–54·0)	5·7% (9·2–17·2)
Female sex workers and males with more than one sexual partner	$87·0 (81·0–92·0)	19·0% (13·0–25·0)	$10·2 (9·6–10·7)	31·0% (27·4–35·0)	$13·3 (13·0–15·8)	21·7% (10·1–33·8)
**VOICE scores**						
Females with VOICE score >5 only	$92·0 (84·0–100·0)	16·0% (10·0–22·0)	$14·0 (7·4–13·2)	35·0% (32·0–39·0)	$4·9 (2·6–7·2)	13·0% (0·2–25·4)
Females with VOICE score >5, and males with more than one sexual partner	$65·0 (61·0–69·0)	25·0% (20·0–31·0)	$7·5 (7·2–8·0)	49·0% (49·0–52·0)	$7·2 (5·9–8·9)	28·8% (18·6–40·5)
Females with VOICE score >3 only	$43·0 (40·0–45·0)	27·0% (20·0–34·0)	$8·9 (8·5–9·3)	54·4% (51·5–57·4)	$6·0 (4·7–7·2)	31·0% (19·0–40·0)
Females with VOICE score >3, and males with more than one sexual partner	$41·0 (39·0–42·0)	35·0% (30·0–39·0)	$5·4 (5·2–5·7)	63·4% (60·9–66·0)	$5·3 (4·5–6·2)	40·0% (30·0–51·0)
Females with VOICE score >1 only	$33·0 (32·0–35·0)	35·8% (30·2–41·2)	$6·4 (6·1–6·7)	63·0% (60·0–66·0)	$4·3 (3·2–5·5)	35·0% (26·0–44·0)
Females with VOICE score >1, and males with more than one sexual partner	$32·0 (31·0–33·0)	42·5% (38·3–46·9)	$4·2 (4·0–4·4)	71·0% (69·0–73·0)	$4·0 (3·3–4·9)	45·0% (37·0–53·0)

VOICE score >5 is inclusive of female sex workers. HIV infections averted are compared to scenario of daily oral pre-exposure prophylaxis only among people aged 15–65 years over 10 years of lenacapavir implementation. Prices are in 2021 US$. Values in parentheses show 95% uncertainty intervals representing the 2·5th and 97·5th percentiles across 100 parameter sets. VOICE=Vaginal and Oral Interventions to Control the Epidemic.

## Discussion

In this analysis, we modelled the health and economic impact of lenacapavir scale-up in western Kenya, Zimbabwe, and South Africa. We projected that lenacapavir provision at 1·6–4·0% population coverage could substantially decrease HIV incidence and be cost-effectively implemented at a per-dose price of approximately $17 in western Kenya, $21 in Zimbabwe, and $106 in South Africa. A 2024 study estimated that mass production of generic lenacapavir could be achieved at a per-dose price of $17·50–20,^12^ in line with our price thresholds for western Kenya and Zimbabwe. Although lenacapavir introduction can result in some cost savings in the long term from ART and hospitalisations averted, the projected short-term budget impact was substantial, with costs increasing as lenacapavir is scaled up. In an era of shrinking donor funding for HIV programmes, policy makers might weigh the impacts of lenacapavir implementation against other non-HIV health interventions that often have a lower cost-effectiveness threshold. Using the threshold of $200 per DALY averted reduced the price threshold for lenacapavir to approximately $11 (western Kenya), $12 (Zimbabwe), and $75 (South Africa).

HIV incidence reductions and price thresholds were similar in western Kenya and Zimbabwe; however, the price threshold was five times higher in South Africa, probably owing to higher HIV prevalence and health-care costs. The number of lenacapavir doses needed was highest in South Africa (seven times higher than in Zimbabwe) due to the larger population size, which could inform policy negotiations as South Africa could present a sizable market for lenacapavir that might sustain initially higher prices while production scales up. Using a higher cost threshold of $1175 increased the price threshold for South Africa to approximately $177, higher than in the main analysis. These findings also highlight the importance of country-specific analyses as budgetary impact, product volume, and price thresholds vary across settings due to differences in costs and HIV epidemics.

Health impacts and maximum price were sensitive to assumed lenacapavir uptake among subgroups with HIV risk indication. A strength of this analysis is that we conducted a literature review of long-acting PrEP preferences among key groups and the general population in eastern and southern Africa to inform model inputs. In the higher uptake scenario, HIV infections averted increased by approximately 10 percentage points across settings (compared with the main analysis), but per-dose price thresholds were approximately 10–18% lower and doses required doubled. The relationships between health impacts, product volume, and price threshold are illustrated by our sensitivity analysis exploring expansion by VOICE score. Lenacapavir provision to female sex workers could sustain the highest price per dose due to high HIV incidence in this group, but population-level infections averted were low due to the relatively small number of female sex workers. Price thresholds substantially decline with expanding lenacapavir coverage to populations with lower HIV risk, and infections averted showed diminishing marginal returns. Additionally, increased demand generation costs might be needed to reach broader coverage levels, which would increase programme costs. Interestingly, including males with more than one sexual partner was more efficient than expanding to the female category with the next highest risk in Zimbabwe and South Africa but not in western Kenya. This finding might be due to high coverage of voluntary medical male circumcision in western Kenya, which reduces HIV acquisition risk. At coverage of females with VOICE score greater than 1 and males with more than one sexual partner, the price threshold decreased to approximately $4 in western Kenya and Zimbabwe and approximately $32 in South Africa, consistent with previous analyses showing broad PrEP distribution in eastern and southern Africa is unlikely to be cost-effective unless PrEP costs are low.^27^, ^28^ Taken together, these sensitivity analyses can provide insights to policy deliberations regarding the product volume that can be cost-effectively purchased at different price thresholds because countries might be able to commit to obtaining more doses of lenacapavir at lower prices.

To our knowledge, this is the first modelling study to investigate the impact of lenacapavir scale-up in eastern and southern Africa informed by data on PrEP preferences across subgroups. Our findings are within the range of a previous modelling study of long-acting cabotegravir implementation in a general eastern and southern Africa setting, which projected that it would be cost-effective at 2·5% coverage at a cost of $57 per 6 months of use,^29^ which is lower than our estimate for South Africa but higher than that of Kenya and Zimbabwe. All other modelling studies of injectable long-acting PrEP we found in the literature evaluated the setting of South Africa and showed that long-acting PrEP could be cost-effective if provided to female sex workers and young women with HIV risk indication.^30^ One study estimated that the threshold cost of long-acting cabotegravir in South Africa was $33–50 for 6 months of use, using the threshold of the same cost-effectiveness as oral PrEP.^25^ Our results contribute to the literature by estimating the impact of long-acting PrEP in eastern and southern African settings outside South Africa, which differs considerably from other regions in terms of health system costs and HIV epidemic.

We used PrEP provision costs from community settings such as pharmacies and assumed that making lenacapavir widely accessible will result in individuals aligning use with HIV risk. This is consistent with previous studies of oral PrEP in eastern and southern Africa that found that making PrEP easily accessible resulted in substantial reductions in HIV incidence in the population despite low uptake rates, suggesting that individuals were effectively using PrEP during periods of HIV risk.^31^, ^32^ We assumed a demand generation cost equivalent to 10% of the estimated per-dose price to support educational campaigns to help individuals identify and target PrEP to times of HIV risk. If additional campaigns are needed to increase lenacapavir use among some subgroups, or if lenacapavir is distributed through higher-resource home or mobile provision, these costs might be underestimated, as highlighted in our sensitivity analyses varying provision costs. However, some subgroups such as female sex workers could sustain a higher price per dose, suggesting that more intensive outreach might be cost-effective due to higher incidence in this group. Future patterns of oral PrEP use in the context of long-acting products are uncertain; however, our results were robust to tripling oral PrEP use and varying adherence. This is likely to be due to the low levels of current use despite substantial efforts to increase coverage—tripling of low levels still resulted in a very small proportion of oral PrEP coverage.

Our analysis has several limitations. First, because lenacapavir is not yet available in eastern and southern Africa, we relied on stated preference literature to parameterise lenacapavir uptake, which might not align with observed behaviour. However, evidence shows that stated preferences are strongly correlated with actual user choices.^33^ Additionally, we conducted extensive sensitivity analyses varying lenacapavir uptake across populations with HIV risk indication. Second, we assumed lenacapavir availability in eastern and southern Africa by 2026; delays in lenacapavir scale-up will be likely to result in lower price thresholds due to declining HIV incidence. Third, our model only simulates heterosexual mixing, which accounts for the majority of the HIV epidemic in eastern and southern Africa; therefore, we cannot assess the impact of lenacapavir among men who have sex with men, or people who inject drugs, who are important target groups for long-acting PrEP. Additionally, we did not model pregnant women, who have high HIV incidence in eastern and southern Africa and can benefit from lenacapavir. As with all model-based analyses, the findings are dependent on our model's underlying assumptions, which might be subject to bias or misclassification. Fourth, we used fixed model probabilities instead of assigning a probability distribution. Fifth, we used the payer perspective for our price threshold analysis, which does not include participant costs incurred or averted by accessing lenacapavir. Sixth, we did not evaluate the effect of availability of other long-acting PrEP products, including long-acting cabotegravir or dapivirine ring, on lenacapavir scale-up. Empirical data show a strong preference for injectables and longer duration products, so we assume most individuals with HIV risk would choose lenacapavir over other long-acting PrEP products. Last, in light of these limitations, policy makers will be likely to consider cost-effectiveness analysis alongside many other factors, including feasibility, acceptability, political will, and distributional equity when making health-care decisions.

Strengths of this analysis include using an individual-based network model and evaluating uncertainty across 100 parameter sets in three eastern and southern African settings. By including costs of HIV testing, lenacapavir delivery, demand generation, wastage, and HIV-related health-care costs, we were able to isolate the product price of lenacapavir to better inform decision making regarding lenacapavir purchasing and budgeting.

Overall, we found that lenacapavir could avert substantial HIV incidence, but prices will have to be affordable to ensure equitable and cost-effective distribution. Our findings could inform policy deliberations regarding price thresholds and product volume in an era of novel PrEP products.

### Contributors

### Data sharing

Data used for the modelling can be found in the appendix (pp 11–60). EMOD-HIV is open-source and publicly available at https://docs.idmod.org/projects/emod-hiv/en/2.20_a/.

## Declaration of interests

MS reports funding from the National Institutes of Health and the Bill & Melinda Gates Foundation during the conduct of this study. AB reports funding from the National Institutes of Health, the Gates Foundation, the New York City Department of Health and Mental Hygiene, and the Foundation for Innovative New Diagnostics; and consulting fees from Gates Ventures during the study. All other authors declare no competing interests. The views expressed in this Article do not necessarily represent the views or policies of the institutions with which they are affiliated.
