# Supplementary material for: Health impact, budget impact, and price threshold for cost-effectiveness of lenacapavir for HIV pre-exposure prophylaxis in eastern and southern Africa: a modelling analysis
Source: Lancet HIV. 2024 Sep 20;11(11):e765–73. doi: 10.1016/S2352-3018(24)00239-X (PMC11519315; doi:10.1016/S2352-3018(24)00239-X)

THE LANCET
HIV

Supplementary appendix

This appendix formed part of the original submission and has been peer reviewed. We post it as supplied by the authors.

Supplement to: Wu L, Kaftan D, Wittenauer R, et al. Health impact, budget impact, and price threshold for cost-effectiveness of lenacapavir for HIV pre-exposure prophylaxis in eastern and southern Africa: a modelling analysis. *Lancet HIV* 2024; published online Sept 20. https://doi.org/10.1016/S2352-3018(24)00239-X.

Online Supplemental Appendix

Accompanying the manuscript:

Health and budget impact, and price threshold for cost-effectiveness of lenacapavir for PrEP in Eastern and Southern Africa: a modeling analysis

Contents

Supplemental Appendix Part I 4

Lenacapavir uptake estimates by subgroup 4

Table S1: Summaries of studies evaluating healthcare accessibility in Eastern and Southern Africa 5

Table S2: Characteristics of studies assessing PrEP preference in Sub-Saharan Africa 6

Table S3: Lenacapavir uptake by subgroup in Eastern and Southern Africa 10

Rationale for countries selected: 10

Empiric Data for Model Calibration and Validation 11

Table S4: HIV incidence by region 11

HIV prevalence in counties by age and gender 11

Table S5a. HIV prevalence in counties of the former Nyanza province (Homa Bay, Kisii, Kisumu, Migori, Nyamira, Siaya), by age and gender in Kenya[¥] 11

Table S5b. HIV prevalence data by age and sex from population-based surveys for model calibration in South Africa[¥] 12

Table S5c. HIV prevalence data by age and sex from population-based surveys for model calibration in Zimbabwe[¥] 13

Table S6. HIV prevalence among men and women ages 15-49, by county and gender in Kenya[¥] 13

Number of people on ART 14

Table S7a. Number of people on ART by county, gender, and age group in Kenya[¥] 14

Table S7b: Number of people on ART by sex (ages 15-49 years) in South Africa [¥] 16

Table S7c: Number of people on ART by year and sex (ages 15-49 years) in Zimbabwe [¥] 16

Population size by age and gender 17

Table S8a. Population size by gender, county, and age group in 2009 in western Kenya[¥] 17

Table S8b. Population of South Africa by age and sex [¥] 18

Table S8c. Population of Zimbabwe by age and sex in 2010[¥] 19

Voluntary medical male circumcision 20

Table S9a. Circumcision status quo by county, age group (years), and year in Kenya [¥] 20

Table S9b. Number of voluntary medical male circumcisions conducted in South Africa by age group[*] 22

Table S9c. Number of voluntary medical male circumcisions conducted in Zimbabwe among male aged 15-25 years old[*] 23

Age-specific population fertility rates 24

Table S10a. Age-specific population fertility rates in Kenya 1950-2044[¥] 24

Table S10b. Age-specific population fertility rates in South Africa 1950-2044[¥] 25

Table S10c. Age-specific population fertility rates in Zimbabwe 1950-2044[¥] 26
HIV-deleted mortality rates calculations 27
Table S11a. Age-specific HIV deleted mortality rates in Kenya 1950-2049 by gender [¥] 27
Table S11b. Age-specific mortality rates in South Africa 1950-2049 by gender[¥] 28
Table S11C. Age-specific HIV-deleted mortality rates in Zimbabwe 1950-2049 by gender 30
Model fit to age-specific and overall prevalence from population-based surveys by sex 31
Figure S1a Model fit to age-specific and overall prevalence from population-based surveys by sex in Kenya 31
Figure S1b Model fit to age-specific and overall prevalence from population-based surveys by sex in South Africa 35
Figure S1c. Model fit to age-specific and overall prevalence from population-based surveys by sex in Zimbabwe 39
Model fit to age-specific and overall prevalence from population-based surveys by sex 44
Figure S2a Model fit to age-specific and overall ART coverage from population-based surveys by sex in Kenya[*] 44
Figure S2b Model fit to age-specific and overall ART coverage from population-based surveys by sex in South Africa 44
Figure S2c Model fit to age-specific and overall ART coverage from population-based surveys by sex in Zimbabwe 45
Model Overview and Parameters 45
Model Parameters 47
Table S12a. Select model parameters used to fit the EMOD-HIV transmission model to survey data on prevalence and ART coverage from Kenya. 47
Table S12b. Select model parameters used to fit the EMOD-HIV transmission model to survey data on prevalence and ART coverage from South Africa. 49
Table S12c. Select model parameters used to fit the EMOD-HIV transmission model to survey data on prevalence and ART coverage from Zimbabwe. 52
Table S13. Utility weights for estimating disability-adjusted life-years averted 54
Costing parameters 55
Table S14 Kenya costing parameter calculations 55
Table S15 South Africa costing parameter calculations 56
Table S16 Zimbabwe costing parameter calculations 57
Number of Oral PrEP initiations in Western Kenya, South Africa, and Zimbabwe 58
Table S17 Number of PrEP initiations in Western Kenya, South Africa, and Zimbabwe 59
VOICE Risk Score 59
FSW and male client of FSW estimation 59
Table S18: Lifetime probability of becoming a female sex worker or male client of FSW in western Kenya by county 59
Table S19: Lifetime probability of becoming a female sex worker or male client of FSW in South Africa 60
Table S20: Lifetime probability of becoming a female sex worker or male client of FSW in Zimbabwe 60
References 61
Supplemental Appendix Part II Additional Results 65

Table S1: Five-year budget impact analysis - main scenario* 66
Table S2: Five-year budget impact analysis - Higher lenacapavir coverage scenario* 67
Table S3 Five-year budget impact analysis of a $50 per does price- main scenario* 68
Table S4: Health and budget impact and maximum price threshold for South Africa under varying scenarios of background oral PrEP* 69
Table S5: Health and budget impact and maximum price threshold varying lenacapavir effectiveness in men * 70
Figure S1: Sensitivity analyses: Expanding coverage to females only by VOICE score 71
Figure S2: Sensitivity analyses: Expanding lenacapavir coverage to females by VOICE score and males with >1 partner 72
Table S6: Western Kenya: lenacapavir distribution to FSWs and males 73
Table S7: Western Kenya: lenacapavir distribution to females VOICE≥5 and males 74
Table S8: Western Kenya: lenacapavir distribution to females VOICE≥3 and males 75
Table S9: Western Kenya: lenacapavir distribution to females VOICE≥1 and males 76
Table S10: Zimbabwe: lenacapavir distribution to female sex workers (FSWs) and males 77
Table S11: Zimbabwe: lenacapavir distribution to females VOICE≥5 and males 78
Table S12: Zimbabwe: lenacapavir distribution to females VOICE≥3 and males 79
Table S13: Zimbabwe: lenacapavir distribution to females VOICE≥1 and males 80
Table S14: South Africa: lenacapavir distribution to female sex workers (FSWs) and males 81
Table S15: South Africa: lenacapavir distribution to females VOICE≥5 and males 82
Table S16: South Africa: lenacapavir distribution to females VOICE≥3 and males 83
Table S17: South Africa: lenacapavir distribution to females VOICE≥1 and males 84
Table S18: Summary of HIV incidence reduction and maximum price per dose for expanding Lenacapavir distribution scenarios 85
Figure S3: Five-Year Budget Impact for higher Lenacapavir coverage scenario: Western Kenya 86
Figure S4: Five-Year Budget Impact for higher Lenacapavir coverage scenario: South Africa 87
Figure S5: Five-Year Budget Impact for higher Lenacapavir coverage scenario: Zimbabwe 88
Figure S6: Component Costs by Scenario in western Kenya 89
Figure S7: Component Costs by Scenario in western Kenya 90
Figure S8: Component Costs by Scenario in Zimbabwe 91
Figure S9 Tornado diagram of one-way sensitivity analyses of price threshold in three countries 92

Supplemental Appendix Part I

Lenacapavir uptake estimates by subgroup

We conducted a scoping literature review of user preferences for oral vs. long-acting PrEP in Eastern and Southern Africa. We identified 34 publications published between 2014-2024 that assessed demand for long-acting PrEP products among the general population, adolescent girls and young women, and female sex workers. The preprint review is available here:

Brian Pfau, Arden Saravis, Sarah N. Cox, Linxuan Wu, Rachel Wittenauer, Emily Callen, Cory Arrouzet, Monisha Sharma. User Preferences on Long-Acting Pre-Exposure Prophylaxis for HIV Prevention in Sub-Saharan Africa: A Scoping Review medRxiv 2024.04.01.24305173; doi: https://doi.org/10.1101/2024.04.01.24305173 (1)

Of 34 articles identified,15 evaluated long-acting injectable (LAI) PrEP and were considered relevant to the present analysis **(Table S1)**. For each of the 15 studies, we estimated the proportion of participants who reported an interest in using LAI PrEP if it were to become available, along with upper and lower ranges of uncertainty. We then summarized proportions across studies for each subgroup of interest. Across subgroups, long-acting injectable PrEP was more commonly preferred over daily oral PrEP, although was not universally preferred, with a minority of participants reporting a desire to use daily oral PrEP over LA PrEP.

We assumed healthcare accessibility among the population was not 100%, therefore a proportion of individuals with a desire to use LAI PrEP would be unable to access it. We conducted a literature review on healthcare accessibility among sugroups in ESA, including female sex workers (FSW), male clients of FSW, adolescent girls and young women (AGYW), and the general population. We found 7 studies assessing healthcare accessibility in adults and female sex workers seeking facility- or community-based healthcare for any or a specific conditions in ESA **(Table S2)**. We found an overall healthcare utilization of 20-96% among adults in the general population and 40-80% of FSW in SSA. Based on the data, we assumed that healthcare was accessible by 90% of women in the general population and 80% of FSWs, males and adolescent girls and young women.

We multiplied healthcare accessibility by LAI demand to estimate the Lenacapavir uptake by subgroup in ESA **(Table S3).**

Table S1: Summaries of studies evaluating healthcare accessibility in Eastern and Southern Africa

Study	Population and settings	Condition/Service	Relevant Findings
**Adults**			
Bigogo 2010 (2)	Individuals with symptoms of illness in rural western Kenya	Fever, respiratory infections, and diarrhea	Among 208,007 illness episodes identified from 27,171 individuals, 57% sought care at a healthcare facility or community-based organization.
Abuya 2007 (3)	Adults with recent illnesses (i.e., fever) in three districts in Kenya with different levels of malaria endemicity	Fever	57% of adults with fever purchased over-the-counter medicines
Panzner 2016 (4) 8/31/2024 7:47:00 PM	Adults with fever <3 days seeking public or private care in 9 sub-Saharan African countries	Fever	20-88% of the population sought healthcare for fever at a healthcare facility across different sites
Abaerei 2017 (5)	Adult residents in Gauteng province, South Africa	Non-specific	96% reported utilizing any healthcare services from public or private facilities
**Female Sex Workers (FSW)**			
Lafort 2017 (6)	FSW in SA, Mozambique, and Kenya	Facility based sexual reproductive health	26-41% of the participants utilized facility-based SRH service across different study sites
Pande 2019 (7)	FSW in Uganda	HIV test in the past 12 months in facility, community, or outreach events	86% of the participants reported having an HIV test in the last 12 months
Richter 2014 (8)	FSW in Johannesburg, Rustenburg and Cape Town, SA	Received facility or community-based health service in the last month	60% of participants interacted with healthcare services in the last month

Table S2: Characteristics of studies assessing PrEP preference in Sub-Saharan Africa

Author (year)	Study design	Study population	Method/question for preference assessment	Location	Findings	Methods for adapting study estimate for model parameters	% of participants who report preference for LAI PrEP			
							Adult Women	AGYW	Adult Men	FSW
Ngure et al. (2021) (9)	Cross-sectional survey among a subset of women existing a PrEP adherence support study after 2 years' follow up	AGYW aged 18-24 at high HIV risk after finishing an oral PrEP RCT	If all these methods (vaginal ring, injection [such as contraception Depo provera], implants [such as Norplant], oral pills) were available, which one would you prefer? Rank in order of preference with 1 being the most preferred	Kenya	59 (36.0%) of participants preferred injectables, 56 (34.2%) preferred oral pills, 36 (22.0%) preferred implants, and 24 (14.6%) preferred vaginal rings	We took the proportion of participants who preferred injectables (36%) as lower bound and proportion of participants who preferred any long-acting PrEP as the upper bound (36%+22%+14.6%=73%)		36-73%		
Wara et al. (2023) (10)	Cross-sectional survey of women participating in the PrEP-PP or PrIMA-X and attending follow up visits	Pregnant/postpartum women with oral prior PrEP experience median age 27-29 yo	Would you prefer to switch to injectable PrEP over remaining on oral PrEP if it were available?	South Africa, Kenya	75% participants preferred long-acting injectable over oral PrEP		75%			
Minnis et al. (2020) (11)	Discrete choice experiment	Female and male aged 18-24 yo with no prior exposure to product	Assessed preference weights and trade-offs for long-acting PrEP: injectable and implant	South Africa	Youth indicated strong interest in using a long-acting HIV prevention product; for 96% of the choice tasks (7032/7263), respondents stated they would be willing to use their chosen product if available.			96%		

Montgomery et al. (2021) (12)	discrete choice experiment	MSM and MSW aged 18-24 years old; MSW product naive; MSM with PrEP experience	Assessed 5 attributes of LA PrEP (injectable vs. implant) including the delivery form, duration, insertion location, soreness and deliveyr facility	South Africa	Duration-dominant decision-makers” (46%) were largest class, defined by significant preference for a longer-duration product. “Comprehensive decision-makers” (36%) had preferences shaped equally by multiple attributes, and preferred implants. “Injection-dominant decision-makers” (18%) had strong preference for injections (vs. implant) and were significantly more likely to be MSM. When estimating shares for a 2-month injection in the buttocks with mild soreness (HPTN regimen) vs. a 6-month implant (to arm) with moderate soreness (current target), 95% of “injection-dominant” would choose injections, whereas 79% and 63% of “duration-dominant” and “comprehensive”, would choose implant.	We calculated the proportion of participants who preferred LAI by using the proportion of decision-maker multiplied by the corresponding proportion of who preferred LAI, 0.46*0.95+0.36*0.21+0.18*0.37=58%			58%	
Cheng et al. (2019) (13)	Cross sectional survey from a discrete choice experiment	Adult men, mostly MSW	Rank from most to least preferred: LAI, oral, and condoms	South Africa	48% (n= 85) of participants preferred LAI PrEP, while 33% (n= 58) and 20% (n= 35) chose oral PrEP and condoms.				48%	
Tolley et al. (2019) (14)	Cross sectional survey among women participating in phase RCT of LA PrEP (HPTN 076) at baseline	Low HIV risk women median age 31 (24-37) years old	Quantitative question: initial preferences for future HIV prevention product use	South Africa, Zimbabwe, US	Among African women at baseline, 81% prefer bi month injection, 11% for daily oral pill, 2% for vaginal ring , 0% for vaginal gel , and 6% for other options (e.g.,implant, sterilization, IUD)	We took proportion of participants who preferred injectables (81%) as the lower bound and proportion of participants who preferred any long acting PrEP as the upper bound (81+2=83%)	81-83%			
van der Straten et al (2018) (15)	Preference of product after trying placebo product (injectable, ring, and oral)	Sexually active women aged 18-30 years	what were women's preferences for TRIO products compared to each other and to male condoms?	South Africa, Zimbabwe, Kenya	Overall, 85% preferred a TRIO product over condoms, and all three products were chosen by a significant number of women (injections 64%, tablets 21%, vaginal ring 15%)	We calculated the proportion of participants who preferred LAI by using the proportion of women who preferred a TRIO product multiplied by the corresponding proportion who preferred LAI as the lower bound and any long-acting product as the upper bound.<br>% prefer LAI: 85%*64% =54%,<br>% prefer any long-acting PrEP 85%*(64%+15%)=67%	54-67%			

Jansen van Vuuren et al. (2023) (16)	Cross-sectional survey among women enrolled in PrEP implementation project	Sexually active women aged 18-30 years	participants were asked to choose one method which would be most suitable to incorporate into their lifestyle	South Africa	The %(n) for each PrEP modality is: Three-monthly injection 31.3 (133), two-monthly injection 5.4 (23), daily oral PrEP 37.2 (158), pill before and after sex 8.0 (34), six-monthly implant 10.6 (45), monthly vaginal rings 5.4 (23), no method chosen 1.6(7), and missing 0.5 (2)	We took the proportion of participants who preferred injectables (31.3+5.4=37%) as the lower bound and proportion of participants who preferred any long acting PrEP as the upper bound (31.3+5.4+10.6+5.4=52.7).	37-53%			
Kidman et al. (2020) (17)	cross sectional survey among a subset of children enrolled in a cohort study	Girls age 10-16 years	If participants were willing to get an injection, and if they would prefer a daily pill to an injection	Malawi	Willing to have injection among sexually active girls is 91%; 53-65% of girl prefer injectable vs. oral daily	We took willingness to have injection as upper bound and preference for injectable over oral as the lower bound		53-91%		
Mayanja et al. (2022) (18)	Cross sectional: Assessed preference during first two weeks of enrollment in PrEP cohort study among PrEP naive women	Sexually active women aged 14-24 (high HIV risk, 94% had paid sex)	Ranking 5 methods (oral PrEP, LAI, ring, implant, and vaccine) on a 1 to 5 scale (1=most preferred, 5=least preferred); considered preferred is ranked the top two choices	Uganda	Participants were offered oral PrEP during the study; 47.6% preferred oral PrEP	Assume LA as 1-47.6%=52%				52%
Harling et al. (2019) (19)	Cross sectional survey among female bar workers (FBW)	FBW aged 23-29 yo with low exposure to PrEP before (5% heard of PrEP)	how interested in taking PrEP as daily pill, injection every 2 mo, vaginal gel, and monthly ring; rank the four modalities from most to least preferred.	Tanzania	79% of FBW interested in LAI; 42% of FBW listed LAI as their first choice and 18% listed LAI as their second choice	We took those who were interested in LAI as the upper bound (i.e., 80%) and those listed LAI as their first and second choices as the lower bound(i.e., 60%)				60-80%
Luecke et al. (2016) (20)	Cross sectional survey among women finishing the VOICE trial	Women aged 21–41	select what type of product formulation, if any, they would be interested to take and explain their choice(s). Women could select as many	South Africa, Uganda, Zimbabwe	A majority of women (81%) expressed a preference for injectable, implant, or vaginal ring; 40% preferred to use LAI	We took 80% as the upper bound (80%) and 40% as the lower bound.	40-80%			

			products as they wanted							
Beckham et al. (2022) (21)	Cross sectional survey among FSW	FSW with little exposure to PrEP	If you personally were going to take ART to prevent HIV infection, would you prefer to take it in the form of a daily pill or an injection once every 3 months? Injection/pill	Tanzania	Most (88%) preferred LA vs oral PrEP					88%
Siedner et al. (2018) (22)	Cross sectional survey among clients from public sector clinic	Clients from public sector primary care with 86% being female and median age of 28 years (IQR 22-35); at least 22% had exposure to oral PrEP	If in the future an injectable PrEP would become available, would you have a preference for either oral or injectable PrEP?	Eswatini	Seventy-five percent of respondents (95%CI 65–83%) expressed a preference for LAI every two months over daily oral PrEP. This preference was reported in both sexes (P=0.16)		65-83%		65-83%	
Were et al. (2023) (23)	Cross sectional survey from PrEP delivery site	Individuals eligible for PrEP from PrEP delivery clinics	Participants were presented with all PrEP options and asked to rank from most preferred to least preferred of injection, implant, monthly oral, and ED-PrEP (men only); Particiapnts also asked willingness to use each product	Kenya	50% of AGYW, 54% of FSW, and 45% of male most prefer LAI. Slightly more than half(3,026;50.3%) of the participants indicated willingness to use two-month injectable PrEP (cabotegravir), with more females 2,052(67.8%) expressing preference.		68%	50%	45-50%	54%

Rousseau (2024) (24)	prospective cohort study	Young people	Let participants to choose from the following: oral PrEP, dapivirine vaginal ring (DVR), and CAB-LA	South Africa	As of July 20th, among 717 enrolled AGYW, 550 (77%) choose to use CAB-LA, 13 (2%) choose to use DVR, and 154 (21%) choose to use oral PrEP.	We took the proportion who used CAB-LA and DVR as the upper bound (i.e., 79%) and proportion who used CAB-LA as the lower bound(i.e., 77%)		77-79%		

Table S3: Lenacapavir uptake by subgroup in Eastern and Southern Africa

Population	Healthcare accessibility	% participants preferred long-acting injectable PrEP		Lenacapavir uptake	
		Lower	Upper	Lower	Upper
FSW	80%	50%	90%	40%	72%
Male clients of FSW	80%	50%	90%	40%	72%
AGYW	80%	40%	95%	32%	76%
Women with >1 partner	90%	40%	80%	36%	72%
Men with >1 partner	80%	40%	80%	32%	64%

The denominator for percentages uptake among subgroup include all those in the subgroup of interest regardless of HIV status. Therefore groups with higher HIV prevalence will have higher uptake in the remainder of the subgroup without HIV.

Rationale for countries selected:

South Africa, Zimbabwe, and western Kenya were chosen for the present analysis since they were early adopters of oral PrEP and represent geographically diverse regions in ESA with high HIV incidence and prevalence. We included South Africa as it has the potential to be a sizable market for lenacapavir and will likely have a distinct price threshold from other countries in ESA. We chose western Kenya as it has the highest HIV incidence in Kenya and has been a leader in oral PrEP rollout.

Empiric Data for Model Calibration and Validation

Table S4: HIV incidence by region

**Setting**	**Incidence per 1000 Adults aged 15 – 49**
Nyanza, Kenya	2.34 [1.90 – 3.08]
South Africa	7.79 [4.58 - 10.80]
Zimbabwe	2.75 [1.95 - 3.92]

Source: https://aidsinfo.unaids.org/

HIV prevalence in counties by age and gender

Table S5a. HIV prevalence in counties of the former Nyanza province (Homa Bay, Kisii, Kisumu, Migori, Nyamira, Siaya), by age and gender in Kenya[¥]

**Age group (years)**	**2003**		**2007**		**2008**		**2012**		**2018**	
	**Men**	**Women**	**Men**	**Women**	**Men**	**Women**	**Men**	**Women**	**Men**	**Women**
**15 - 19**	0.0015	0.0459	0.0121	0.0773	0.0184	0.1078	0.0151	0.0486	0.0025	0.0294
**20 - 24**	0.0562	0.2997	0.0257	0.2056	0.0578	0.1201	0.0289	0.1411	0.0216	0.0910
**25 - 29**	0.2429	0.2301	0.1956	0.2454	0.2450	0.2228	0.2107	0.2454	0.0705	0.2334
**30 - 34**	0.1840	0.1632	0.2578	0.2576	0.1530	0.2593	0.2392	0.2047	0.0911	0.2696
**35- 39**	0.2064	0.1838	0.2384	0.2227	0.2275	0.2259	0.1955	0.2811	0.1675	0.2705
**40 - 44**	0.2533	0.3500	0.2024	0.1799	0.2501	0.0927	0.3132	0.1694	0.2075	0.2765
**45 - 49**	0.1624	0.1651	0.2103	0.1291	0.1331	0.1716	0.1623	0.2287	0.2796	0.1991
**15 - 49**	0.1160	0.1830	0.1140	0.1760	0.1140	0.1600	0.1340	0.1760	0.0826	0.1667

[¥]**Sources:** Kenya Demographic and Health Surveys, 2003 & 2008; Kenya AIDS Indicator Surveys, 2007 & 2012; Kenya Population-Based HIV Impact Assessment 2018.

Table S5b. HIV prevalence data by age and sex from population-based surveys for model calibration in South Africa[¥]

Sex	Age group (years)	Year				
		2002	2005	2008	2012	2017
Men	15-19	0.040	0.032	0.025	0.007	0.047
	20-24	0.080	0.060	0.051	0.051	0.048
	25-29	0.220	0.121	0.157	0.173	0.124
	30-34	0.240	0.233	0.258	0.256	0.184
	35-39	0.180	0.233	0.185	0.288	0.238
	40-44	0.120	0.175	0.192	0.158	0.224
	45-49	0.120	0.103	0.084	0.134	0.248
	50-54	0.050	0.142	0.104	0.155	0.202
	55-59	0.070	0.064	0.062	0.055	0.148
	15-49	0.128	0.117	0.116	0.145	0.148
Women	15-19	0.070	0.094	0.067	0.056	0.058
	20-24	0.170	0.239	0.211	0.174	0.156
	25-29	0.320	0.333	0.327	0.284	0.275
	30-34	0.240	0.260	0.291	0.360	0.347
	35-39	0.140	0.193	0.248	0.316	0.394
	40-44	0.190	0.124	0.163	0.280	0.359
	45-49	0.110	0.087	0.141	0.197	0.303
	50-54	0.080	0.075	0.102	0.148	0.222
	55-59	0.070	0.030	0.077	0.097	0.176
	15-49	0.177	0.202	0.213	0.232	0.263

[¥]Sources: South African National HIV Prevalence, Incidence and Behaviour Surveys (2002, 2005, 2008, 2012 and 2017) from the Human Sciences Research Council (HSRC)

Table S5c. HIV prevalence data by age and sex from population-based surveys for model calibration in Zimbabwe[¥]

Age group (years)	2006		2011		2015		2016	
	Men	Women	Men	Women	Men	Women	Men	Women
**15 - 19**	0.031	0.062	0.034	0.042	0.025	0.040	0.032	0.039
**20 - 24**	0.058	0.163	0.038	0.106	0.037	0.103	0.027	0.081
**25 - 29**	0.130	0.288	0.103	0.201	0.075	0.155	0.066	0.143
**30 - 34**	0.295	0.355	0.174	0.290	0.131	0.219	0.122	0.219
**35- 39**	0.321	0.345	0.251	0.291	0.180	0.280	0.194	0.266
**40 - 44**	0.329	0.257	0.262	0.257	0.270	0.313	0.254	0.296
**45 - 49**	0.260	0.180	0.299	0.225	0.232	0.243	0.281	0.289
**15 - 49**	0.145	0.211	0.123	0.177	0.105	0.167	-	-

Zimbabwe Demographic and Health Surveys 2005-06, 2010-22, 2015. Zimbabwe Population-Based HIV Impact Assessment 2016.

Table S6. HIV prevalence among men and women ages 15-49, by county and gender in Kenya[¥]

County	2003		2007		2008		2012		2018	
	Men	Women	Men	Women	Men	Women	Men	Women	Men	Women
**Homa Bay**	0.1097	0.2458	0.2514	0.3259	0.1737	0.2524	0.2217	0.2787	0.1279	0.2532
**Kisii**	0.0114	0.0853	0.0445	0.0693	0.0330	0.0573	0.0346	0.0368	0.0458	0.0684
**Kisumu**	0.1663	0.1914	0.1139	0.1847	0.1109	0.1810	0.1940	0.2022	0.0960	0.2096
**Migori**	0.1804	0.1860	0.1685	0.2181	0.1923	0.2228	0.1435	0.1925	0.0706	0.1758
**Nyamira**	0.0029	0.0742	-	-	0.0234	0.0544	0.0419	0.1045	0.0247	0.0432
**Siaya**	0.1824	0.2424	0.1445	0.2130	0.1526	0.1921	0.2596	0.2990	0.0961	0.1905

[¥]**Sources:** Kenya Demographic and Health Surveys, 2003 & 2008; Kenya AIDS Indicator Surveys, 2007 & 2012; Kenya Population-Based HIV Impact Assessment 2018.

Number of people on ART

Table S7a. Number of people on ART by county, gender, and age group in Kenya¥

Gender	County	Age group (years)	Year													
			2004	2005	2006	2007	2008	2009	2010	2011	2012	2013	2014	2015	2016	2017
Men	Homa Bay	0 - 14	-	-	-	-	-	-	-	-	-	-	2,945	3,583	4,109	4,192
		15 - 99	1,067	2,313	5,148	7,194	10,002	14,436	17,178	15,954	17,522	18,279	19,157	22,834	26,441	29,220
	Kisii	0 - 14	-	-	-	-	-	-	-	-	-	-	828	993	1,109	1,083
		15 - 99	-	-	-	-	-	-	-	2,972	-	-	4,614	5,451	6,604	7,169
	Kisumu	0 - 14	-	-	-	-	-	-	-	-	-	-	3,101	3,245	3,525	3,607
		15 - 99	945	2,047	4,557	6,368	8,853	12,779	15,206	14,122	15,511	16,180	21,216	24,550	28,082	31,021
	Migori	0 - 14											2,309	2,295	2,678	2,673
		15 - 99	711	1,541	3,430	4,793	6,664	9,619	11,446	10,630	11,675	12,179	13,929	15,165	17,438	18,455
	Nyamira	0 - 14	-	-	-	-	-	-	-	-	-	-	484	552	578	611
		15 - 99	-	-	-	-	-	-	-	1,362	-	-	2,120	2,585	3,142	3,474
	Siaya	0 - 14	-	-	-	-	-	-	-	-	-	-	2,645	2,950	3,017	3,197

		15 - 99	860	1,864	4,148	5,797	8,060	11,633	13,843	12,856	14,120	14,730	16,163	18,611	21,477	23,762
**Women**	**Homa Bay**	0 - 14	-	-	-	-	-	-	-	-	-	-	3,431	3,835	4,426	4,535
		15 - 99	1,359	2,944	6,551	9,155	12,522	17,202	21,798	31,454	35,182	38,819	40,118	49,956	57,286	61,811
	**Kisii**	0 - 14	-	-	-	-	-	-	-	-	-	-	906	1,079	1,200	1,146
		15 - 99	-	-	-	-	-	-	-	7,902	-	-	11,691	14,350	17,274	19,044
	**Kisumu**	0 - 14	-	-	-	-	-	-	-	-	-	-	3,241	3,393	3,810	3,831
		15 - 99	1,203	2,606	5,799	8,104	11,084	15,227	19,296	27,843	31,143	34,362	41,230	48,424	56,384	60,789
	**Migori**	0 - 14	-	-	-	-	-	-	-	-	-	-	2,526	2,448	2,868	2,884
		15 - 99	905	1,961	4,365	6,100	8,343	11,461	14,524	20,958	23,442	25,865	27,896	31,964	38,637	40,891
	**Nyamira**	0 - 14	-	-	-	-	-	-	-	-	-	-	506	567	601	622
		15 - 99	-	-	-	-	-	-	-	3,766	-	-	5,964	7,210	8,258	8,654
	**Siaya**	0 - 14	-	-	-	-	-	-	-	-	-	-	2,778	3,136	3,299	3,569
		15 - 99	1,095	2,372	5,279	7,377	10,090	13,862	17,566	25,347	28,351	31,281	33,911	39,853	44,892	48,808
**Both**	**All**	15-99	8,954	19,404	43,185	60,350	83,142	116,787	143,877	175,003	194,552	210,769	246,293	301,029	333,333	389,159

¥Source: Kenya Ministry of Health

Table S7b: Number of people on ART by sex (ages 15-49 years) in South Africa ¥

Year	Male	Female
2001	2713	3543
2002	5768	7586
2003	9321	12313
2004	17717	24423
2005	34874	59240
2006	66629	123308
2007	118977	228753
2008	186564	367389
2009	277931	548206
2010	402000	781477
2011	558131	1095411
2012	713294	1415016
2013	876749	1732515
2014	1013499	2003014
2015	1130063	2239669
2016	1238815	2518238
2017	1403702	2998170

¥Source: South Africa Department of Health Surveys

Table S7c: Number of people on ART by year and sex (ages 15-49 years) in Zimbabwe ¥

Year	Male	Female
**2004**	4854	6146
2005	10049	15818
2006	22323	31309
2007	37527	52562
2008	57603	77120
2009	82266	117364
2010	119235	211144
2011	148551	296112

2012	178027	340774
2013	245690	373190
2014	264614	468305
2016	361399	579378
2017	388658	663132

¥Zimbabwe Ministry of Health

Population size by age and gender

Table S8a. Population size by gender, county, and age group in 2009 in western Kenya¥

Age Group (years)	Men						Women					
	Homa Bay	Kisii	Kisumu	Migori	Nyamira	Siaya	Homa Bay	Kisii	Kisumu	Migori	Nyamira	Siaya
**0 - < 1**	18,335	18,236	17,457	19,265	10,313	15,093	18,354	17,993	16,926	19,309	10,263	14,860
**1 - 4**	69,799	69,529	63,054	69,921	41,165	56,269	69,250	69,023	63,172	69,519	40,396	55,901
**5 - 9**	75,926	76,757	67,083	73,872	46,450	60,966	75,973	75,778	67,779	74,333	46,867	60,710
**10 - 14**	68,689	68,473	62,706	64,300	42,590	58,296	67,159	68,072	63,359	63,249	42,198	56,248
**15 - 19**	57,430	59,228	55,597	53,075	36,604	49,220	54,119	60,776	56,741	52,238	36,786	47,825
**20 - 24**	39,573	41,898	47,281	38,690	24,409	32,725	50,309	58,225	57,649	48,004	34,184	41,443
**25 - 29**	30,437	32,792	40,964	30,727	19,515	25,961	36,016	42,878	40,614	34,670	27,273	30,135
**30 - 34**	23,259	26,678	30,412	23,344	16,605	20,359	26,342	30,031	27,515	25,630	19,487	22,328
**34 - 39**	16,013	21,766	21,251	17,024	14,039	14,793	20,010	26,051	20,611	19,313	17,106	17,932
**40 - 44**	11,914	15,718	15,145	12,170	10,470	11,118	16,513	18,360	16,894	14,773	11,377	16,082
**45 - 49**	11,124	16,797	13,361	10,549	11,318	10,390	15,248	19,181	15,298	12,888	11,886	15,486
**50 - 54**	9,705	12,789	11,251	8,565	8,379	9,079	12,942	14,136	12,504	10,314	8,703	14,541
**55 - 59**	8,159	9,527	8,718	6,399	5,999	8,414	9,833	9,528	9,175	7,692	5,819	12,265
**60 - 64**	6,989	7,395	7,054	5,250	5,026	7,712	8,587	7,654	7,597	6,000	5,107	11,081

**65 - 69**	4,325	4,637	4,163	3,382	3,094	5,107	5,957	5,320	5,402	4,508	3,322	7,732
**70 - 74**	4,029	3,945	3,777	2,907	2,753	5,175	5,355	5,017	4,757	3,524	3,153	7,173
**75 - 79**	2,835	2,743	2,392	2,033	1,778	3,549	3,891	3,338	3,356	2,968	1,919	5,464
**80 - 99**	3,726	3,701	2,821	2,624	2,393	4,159	5,316	5,891	4,615	3,636	3,475	6,155

[¥]Source: Kenya National Bureau of Statistics, 2009 Census

Table S8b. Population of South Africa by age and sex [¥]

Sex	Age group (years)	Year				
		2002	2005	2008	2012	2017
**Men**	0 - 4	2634839	2651819	2692300	2784372	2886299
	5 - 9	2559246	2570683	2592577	2644720	2767111
	10 - 14	2582620	2565499	2557928	2578751	2636981
	15 - 19	2546968	2596517	2584945	2572032	2587023
	20 - 24	2348165	2488613	2572307	2606447	2594656
	25 - 29	2069939	2215800	2362377	2530034	2612453
	30 - 34	1759391	1899279	2018461	2227862	2488823
	35 - 39	1488894	1568198	1664587	1845019	2131687
	40 - 44	1270053	1324166	1367150	1493652	1731088
	45 - 49	1077752	1122009	1159644	1234887	1398602
	50 - 54	831061	925655	973184	1038560	1147992
	55 - 59	636945	677128	760379	856728	947123
**Women**	0 - 4	2587465	2602664	2640997	2729586	2825703
	5 - 9	2523770	2535299	2555462	2604517	2719700
	10 - 14	2557891	2533039	2526253	2545219	2603685
	15 - 19	2527828	2568409	2552976	2535920	2555420
	20 - 24	2323512	2451570	2523985	2560705	2550570
	25 - 29	2064262	2167428	2282514	2447992	2550351
	30 - 34	1807453	1906808	1967958	2123170	2394266
	35 - 39	1577255	1648612	1717873	1821711	2039776
	40 - 44	1370835	1434647	1491937	1594914	1745050
	45 - 49	1184477	1243314	1302936	1396666	1541175
	50 - 54	924873	1054059	1124243	1214556	1348848

	55 - 59	727478	783876	898804	1035986	1160340

¥Source: United Nations Population Database

Table S8c. Population of Zimbabwe by age and sex in 2010¥

Age Group (years)	Male	Female
**0-4**	1003778	993503
**5-9**	849773	849884
**10-14**	791114	795446
**15-19**	729897	766954
**20-24**	646316	730145
**25-29**	511221	596614
**30-34**	407730	471291
**35-39**	296577	333524
**40-44**	220200	245985
**45-49**	163800	200252
**50-54**	125947	167229
**55-59**	99658	142147
**60-64**	68299	100376
**65-69**	60276	94053
**70-74**	39694	70357
**75-79**	21954	48407
**80-84**	9792	28377
**85-89**	2574	10891
**90-94**	--	2858

Source: Zimbabwe National Statistics Agency.

Voluntary medical male circumcision

Table S9a. Circumcision status quo by county, age group (years), and year in Kenya ¥

	Homa Bay			Kisii			Kisumu			Migori			Nyamira			Siaya		
**Year**	**10-14**	**15-24**	**25-49**	**10-14**	**15-24**	**25-49**	**10-14**	**15-24**	**25-49**	**10-14**	**15-24**	**25-49**	**10-14**	**15-24**	**25-49**	**10-14**	**15-24**	**25-49**
**Pre-2008**	0.249	0.249	0.249	0.948	0.948	0.948	0.322	0.322	0.322	0.410	0.410	0.410	0.965	0.965	0.965	0.252	0.252	0.252
**2008**	0.118	0.235	0.275	0.948	0.948	0.948	0.152	0.303	0.333	0.194	0.385	0.451	0.965	0.965	0.965	0.119	0.243	0.270
**2009**	0.122	0.240	0.283	0.948	0.948	0.948	0.178	0.315	0.339	0.199	0.388	0.463	0.965	0.965	0.965	0.127	0.250	0.277
**2010**	0.166	0.285	0.299	0.948	0.948	0.948	0.324	0.389	0.362	0.214	0.395	0.478	0.965	0.965	0.965	0.222	0.310	0.293
**2011**	0.226	0.355	0.313	0.948	0.948	0.948	0.450	0.479	0.385	0.280	0.423	0.486	0.965	0.965	0.965	0.293	0.375	0.304
**2012**	0.337	0.486	0.339	0.948	0.948	0.948	0.516	0.559	0.409	0.516	0.530	0.509	0.965	0.965	0.965	0.417	0.483	0.324
**2013**	0.368	0.565	0.361	0.948	0.948	0.948	0.564	0.634	0.436	0.587	0.614	0.528	0.965	0.965	0.965	0.435	0.549	0.340
**2014**	0.471	0.710	0.399	0.948	0.948	0.948	0.620	0.712	0.467	0.717	0.726	0.554	0.965	0.965	0.965	0.537	0.659	0.368
**2015**	0.506	0.811	0.438	0.948	0.948	0.948	0.672	0.790	0.502	0.742	0.813	0.580	0.965	0.965	0.965	0.562	0.739	0.395
**2016**	0.476	0.894	0.488	0.948	0.948	0.948	0.756	0.844	0.538	0.697	0.891	0.613	0.965	0.965	0.965	0.628	0.841	0.425
**2017**	0.489	0.937	0.537	0.948	0.948	0.948	0.863	0.889	0.572	0.680	0.949	0.648	0.965	0.965	0.965	0.759	0.917	0.457
**2018**	0.589	0.925	0.637	0.948	0.948	0.948	0.944	0.925	0.651	0.775	0.976	0.744	0.965	0.965	0.965	0.844	0.959	0.562
**2019**	0.741	0.904	0.678				0.784	0.943	0.677	0.785		0.775				0.790	0.976	0.596
**2020**	0.802	0.901	0.717				0.787	0.925	0.703	0.800		0.808				0.800		0.633

**2021**	0.802	0.909	0.749				0.836	0.913	0.727			0.836						0.668
**2022**		0.917	0.779				0.804	0.947	0.750			0.865						0.704
**2023**		0.926	0.807				0.804		0.773			0.892						0.738
**2024**		0.935	0.833				0.804		0.795			0.918						0.772
**2025**		0.944	0.858				0.804		0.815			0.942						0.803
**2026**		0.949	0.879				0.801		0.832			0.961						0.832
**2027**		0.953	0.898				0.801		0.848			0.976						0.976
**2028**		0.955	0.915				0.801		0.862									
**2029**			0.931				0.801		0.874									
**2030**			0.945				0.801		0.885									
**2031**			0.955				0.802		0.897									
**2032**									0.907									
**2033**									0.917									
**2034**									0.924									
**2035**									0.931									

¥**Source:** Circumcision prevalence prior to 2008 is obtained from the Kenya Demographic and Health Survey, 2003. Prevalence of circumcision from 2008 onward combines prevalence of traditional male circumcision and voluntary medical male circumcision estimates obtained from the Decision-Makers' Program Planning Toolkit 2.

Table S9b. Number of voluntary medical male circumcisions conducted in South Africa by age group*

Year	Age Group (years)					
	10 - 14	15 - 19	20 - 24	25 - 34	35 - 49	≥50
2010	55,431	30,552	15,856	18,047	7,864	1,160
2011	137,648	75,866	39,374	44,816	19,527	2,881
2012	175,060	96,487	50,075	56,996	24,834	3,664
2013	156,496	86,255	44,765	50,952	22,201	3,276
2014	199,750	110,095	57,138	65,035	28,337	4,181
2015	199,535	109,976	57,076	64,965	28,306	4,176
2016	165,672	91,312	47,390	53,940	23,502	3,468
2017	203,960	112,415	58,342	66,406	28,934	4,269
2018	279,500	154,050	79,950	91,000	39,650	5,850
2019	258,000	142,200	73,800	84,000	36,600	5,400
2020	236,500	130,350	67,650	77,000	33,550	4,950
2021	107,500	59,250	30,750	35,000	15,250	2,250
2022 onwards	43,000	23,700	12,300	14,000	6,100	900

*Source: South Africa Department of Health (unpublished data) and South Africa National Strategic Plan for HIV, TB, and STIs 2017-2022(25).

Table S9c. Number of voluntary medical male circumcisions conducted in Zimbabwe among male aged 15-25 years old*

Year	Target # of males (15-25 years old)
**2008.5**	2784
**2009.5**	9381
**2010.5**	27973
**2011.5**	29321
**2012.5**	65679
**2013.5**	110163
**2014.5**	133012
**2015.5**	136986
**2016.5**	159476
**2017.5**	197705
**2018.5**	207375
**2019.5**	53743
**2020.5**	53743
**2021.5-2040.5**	4288000

Source: McGillen JB, Stover J, Klein DJ, Xaba Sinokuthemba, et al. The emerging health impact of voluntary medical male circumcision in Zimbabwe: An evaluation using three epidemiological models. Jul 2018 PLOS One. https://doi.org/10.1371/journal.pone.0199453

Age-specific population fertility rates

Table S10a. Age-specific population fertility rates in Kenya 1950-2044[¥]

	Age-specific fertility rates (births per 1,000 women) (age groups in years)						
**Year**	**15-19**	**20-24**	**25-29**	**30-34**	**35-39**	**40-44**	**45-49**
**1950-1955**	169.1	351.6	338.1	284.3	203.5	110.7	38.9
**1955-1960**	175.9	365.9	351.9	295.8	211.8	115.2	40.5
**1960-1965**	182.3	379.1	364.5	306.5	219.4	119.4	41.9
**1965-1970**	183.3	381.2	366.6	308.2	220.6	120	42.2
**1970-1975**	180.6	375.5	361.1	303.6	217.3	118.3	41.5
**1975-1980**	172.7	359.1	345.3	290.3	207.8	113.1	39.7
**1980-1985**	163.1	339.2	326.2	274.2	196.3	106.8	37.5
**1985-1990**	147.8	307.3	295.5	248.4	177.8	96.8	34.0
**1990-1995**	115.3	268.9	252.0	206.8	161.6	73.4	52.0
**1995-2000**	111.5	260.7	253.3	196.2	143.3	62.4	42.7
**2000-2005**	104.2	243.6	236.7	183.4	133.9	58.3	39.9
**2005-2010**	97.1	227.1	221.4	170.6	123.7	53.6	36.5
**2010-2015**	86.2	201.9	202.3	149.2	102.1	42.4	27.9
**2015-2020**	75.1	176.5	179.8	129.6	85.8	34.8	22.4
**2020-2024**	69.9	165.1	171.8	120.2	76.2	29.9	18.6
**2025-2029**	65.0	154.7	164.8	112.6	68.5	26.0	15.5
**2030-2034**	60.6	145.7	159.1	106.7	62.5	22.9	13.1
**2035-2039**	56.2	137.2	153.6	101.8	57.6	20.4	11.0
**2040-2044**	52.2	129.7	149.3	98.2	53.8	18.5	9.4

[¥]**Source**: 2019 World Population Prospects

Table S10b. Age-specific population fertility rates in South Africa 1950-2044[¥]

	Age-specific fertility rates (births per 1,000 women) (age groups in years)						
**Year**	**15-19**	**20-24**	**25-29**	**30-34**	**35-39**	**40-44**	**45-49**
**1950-1955**	66.8	265	291.9	242.2	189.8	132	72.3
**1955-1960**	65.7	260.8	287.3	238.3	186.7	130	71.2
**1960-1965**	64.7	256.6	282.7	234.5	183.7	127.9	70
**1965-1970**	60.4	239.7	264.1	219.1	171.7	119.5	65.4
**1970-1975**	76.1	233.9	253.6	211	160.2	105.1	54
**1975-1980**	86.1	217.3	231.8	193.6	142.3	87.4	41.5
**1980-1985**	93.6	201.1	211.3	177	125.9	71.7	30.5
**1985-1990**	95.4	179.4	185.7	155.9	107.3	56	20.4
**1990-1995**	90.8	152.2	155.2	130.8	86.9	41	11.7
**1995-2000**	80.6	140.5	142.5	111.5	74.4	31	10.3
**2000-2005**	70.7	139	141.8	105.6	67.4	27.1	8.8
**2005-2010**	59.2	131.7	135.1	95.9	58.4	22.6	7.1
**2010-2015**	50.9	129	133.1	90.2	52.3	19.4	5.9
**2015-2020**	43.6	127	131.6	85.3	47	16.6	4.8
**2020-2024**	37.2	125.7	130.8	81.3	42.4	14.1	3.8
**2025-2029**	31.4	124.8	130.4	77.8	38.3	11.9	2.9
**2030-2034**	26.2	124.5	130.4	74.8	34.6	9.8	2.1
**2035-2039**	21.5	124.8	131.3	72.5	31.4	7.9	1.4
**2040-2044**	17	125.7	132.6	70.6	28.5	6.2	0.6

[¥]**Source**: 2012 World Population Prospects

Table S10c. Age-specific population fertility rates in Zimbabwe 1950-2044[¥]

	Age-specific fertility rates (births per 1,000 women) (age groups in years)						
**Year**	**15-19**	**20-24**	**25-29**	**30-34**	**35-39**	**40-44**	**45-49**
**1950-1955**	159.9	296.1	289.3	261.5	188.6	124.2	40.4
**1955-1960**	164.6	304.8	297.8	269.2	194.2	127.8	41.6
**1960-1965**	171.7	317.8	310.5	280.8	202.5	133.3	43.4
**1965-1970**	174.0	322.2	314.8	284.6	205.3	135.1	44.0
**1970-1975**	174.0	322.2	314.8	284.6	205.3	135.1	44.0
**1975-1980**	171.7	317.8	310.5	280.8	202.5	133.3	43.4
**1980-1985**	127.9	280.5	280.7	249.2	188.7	98.5	34.9
**1985-1990**	110.6	241.6	240.0	214.4	161.6	81.1	25.2
**1990-1995**	102.7	207.0	195.0	171.9	126.7	60.5	19.0
**1995-2000**	100.1	190.7	178.9	143.9	103.2	46.1	14.1
**2000-2005**	100.4	190.2	171.8	136.8	92.8	40.8	11.3
**2005-2010**	111.1	202.9	178.2	142.7	94.8	38.3	9.1
**2010-2015**	108.8	209.3	197.8	153.3	104.0	37.5	7.1
**2015-2020**	86.1	184.4	174.0	149.1	90.3	35.9	5.3
**2020-2024**	71.7	166.2	162.5	143.9	83.5	32.8	3.9
**2025-2029**	60.2	150.7	152.8	139.5	77.8	30.3	2.9
**2030-2034**	51.0	137.6	144.7	135.9	73.2	28.1	2.2
**2035-2039**	43.6	126.2	137.7	132.9	69.4	26.2	1.7
**2040-2044**	37.4	115.9	131.3	129.9	66.0	24.5	1.4

[¥]**Source**: 2019 World Population Prospects

HIV-deleted mortality rates calculations

In EMOD, we modelled HIV cause-deleted mortality rates in the background and HIV transmission and related morality rates in the foreground. To calculate the HIV deleted mortality, we first investigated all-cause mortality trends between 1960 and 2000 between countries with and without widespread HIV-AIDS crises. Countries without the epidemic demonstrated an exponential decline in mortality, while those grappling with the crisis experienced an exponential decrease interrupted by a sudden spike in the 1980s. We assumed the difference between these two curves (i.e., the spike) is due to the impact of the HIV-AIDS epidemic. Taking Kenya as an example, we fitted an exponential curve from 1970 to 1980 to represent the cause-deleted mortality. We then conducted a check on the population demographics generated by EMOD, ensuring that the age structure of the population simulated through both cause-deleted mortality and simulated HIV transmission aligns with the UN WPP's population projections post-1980.

Table S11a. Age-specific HIV deleted mortality rates in Kenya 1950-2049 by gender ¥

		Age-specific mortality rates (%) (age groups in years)						
**Sex**	**Year**	**15-19**	**20-24**	**25-29**	**30-34**	**35-39**	**40-44**	**45-49**
Women	**1997.5**	0.136	0.191	0.244	0.294	0.361	0.453	0.542
	**2002.5**	0.116	0.167	0.215	0.259	0.321	0.408	0.493
	**2007.5**	0.100	0.145	0.188	0.229	0.286	0.367	0.449
	**2012.5**	0.085	0.127	0.166	0.202	0.254	0.331	0.408
	**2017.5**	0.073	0.110	0.145	0.178	0.226	0.298	0.371
	**2022.5**	0.063	0.096	0.128	0.158	0.201	0.268	0.338
	**2027.5**	0.054	0.084	0.112	0.139	0.179	0.241	0.307
	**2032.5**	0.046	0.073	0.099	0.123	0.160	0.217	0.280
	**2037.5**	0.039	0.064	0.087	0.108	0.142	0.196	0.254
	**2042.5**	0.034	0.055	0.076	0.096	0.126	0.176	0.231
Men	**1997.5**	0.165	0.253	0.288	0.345	0.433	0.551	0.713
	**2002.5**	0.142	0.219	0.251	0.304	0.385	0.494	0.648
	**2007.5**	0.121	0.189	0.219	0.267	0.343	0.443	0.589
	**2012.5**	0.104	0.163	0.191	0.235	0.305	0.398	0.535
	**2017.5**	0.089	0.141	0.166	0.207	0.271	0.357	0.486
	**2022.5**	0.076	0.122	0.145	0.182	0.241	0.320	0.441

	**2027.5**	0.065	0.106	0.126	0.160	0.215	0.287	0.401
	**2032.5**	0.056	0.091	0.110	0.141	0.191	0.258	0.364
	**2037.5**	0.048	0.079	0.096	0.124	0.170	0.231	0.331
	**2042.5**	0.041	0.068	0.083	0.109	0.151	0.208	0.300

¥**Source**: 2019 World Population Prospects

Table S11b. Age-specific mortality rates in South Africa 1950-2049 by gender¥

		Age-specific mortality rates (%) (age groups in years)						
**Sex**	**Year**	**15-19**	**20-24**	**25-29**	**30-34**	**35-39**	**40-44**	**45-49**
**Women**	**1997.5**	0.116	0.163	0.215	0.263	0.351	0.482	0.702
	**2002.5**	0.096	0.134	0.179	0.222	0.302	0.424	0.629
	**2007.5**	0.079	0.111	0.150	0.187	0.260	0.373	0.563
	**2012.5**	0.066	0.092	0.125	0.158	0.223	0.328	0.504
	**2017.5**	0.054	0.076	0.105	0.133	0.192	0.288	0.451
	**2022.5**	0.045	0.063	0.087	0.113	0.165	0.254	0.404
	**2027.5**	0.037	0.052	0.073	0.095	0.142	0.223	0.362
	**2032.5**	0.031	0.043	0.061	0.080	0.122	0.196	0.324
	**2037.5**	0.025	0.035	0.051	0.068	0.105	0.172	0.290
	**2042.5**	0.021	0.029	0.042	0.057	0.090	0.152	0.260
**Men**	**1997.5**	0.177	0.312	0.361	0.437	0.594	0.872	1.264
	**2002.5**	0.169	0.281	0.327	0.395	0.541	0.803	1.177
	**2007.5**	0.173	0.254	0.296	0.358	0.494	0.739	1.095
	**2012.5**	0.156	0.230	0.268	0.324	0.451	0.681	1.019
	**2017.5**	0.141	0.207	0.243	0.294	0.411	0.627	0.949
	**2022.5**	0.127	0.187	0.220	0.266	0.375	0.578	0.883

	**2027.5**	0.115	0.169	0.199	0.241	0.342	0.532	0.822
	**2032.5**	0.103	0.153	0.180	0.218	0.312	0.490	0.765
	**2037.5**	0.093	0.138	0.163	0.197	0.285	0.451	0.713
	**2042.5**	0.082	0.124	0.148	0.179	0.260	0.415	0.663

¥**Source**: 2012 World Population Prospects

		Age-specific mortality rates (%) (age groups in years)						
**Sex**	**Year**	**15-19**	**20-24**	**25-29**	**30-34**	**35-39**	**40-44**	**45-49**
**Women**	**1997.5**	0.130	0.205	0.293	0.369	0.470	0.580	0.692
	**2002.5**	0.104	0.172	0.251	0.321	0.416	0.521	0.631
	**2007.5**	0.084	0.144	0.216	0.279	0.368	0.467	0.574
	**2012.5**	0.068	0.121	0.186	0.243	0.325	0.420	0.523
	**2017.5**	0.055	0.102	0.159	0.212	0.288	0.377	0.477
	**2022.5**	0.044	0.085	0.137	0.184	0.254	0.338	0.434
	**2027.5**	0.035	0.071	0.118	0.160	0.225	0.303	0.395
	**2032.5**	0.029	0.060	0.101	0.140	0.199	0.272	0.360
	**2037.5**	0.023	0.050	0.087	0.121	0.176	0.244	0.328
	**2042.5**	0.018	0.042	0.075	0.106	0.156	0.219	0.299
**Men**	**1997.5**	0.185	0.285	0.333	0.407	0.521	0.666	0.869
	**2002.5**	0.154	0.240	0.283	0.351	0.456	0.590	0.784
	**2007.5**	0.128	0.203	0.241	0.302	0.399	0.522	0.708
	**2012.5**	0.107	0.171	0.205	0.261	0.349	0.463	0.638
	**2017.5**	0.089	0.144	0.174	0.225	0.305	0.410	0.576
	**2022.5**	0.074	0.122	0.148	0.194	0.267	0.363	0.520
	**2027.5**	0.062	0.103	0.126	0.167	0.234	0.321	0.469
	**2032.5**	0.052	0.087	0.107	0.144	0.204	0.285	0.423
	**2037.5**	0.043	0.073	0.091	0.124	0.179	0.252	0.381
	**2042.5**	0.036	0.062	0.078	0.107	0.156	0.223	0.344

Table S11C. Age-specific HIV-deleted mortality rates in Zimbabwe 1950-2049 by gender
¥**Source**: 2019 World Population Prospects

Model fit to age-specific and overall prevalence from population-based surveys by sex

Figure S1a Model fit to age-specific and overall prevalence from population-based surveys by sex in Kenya

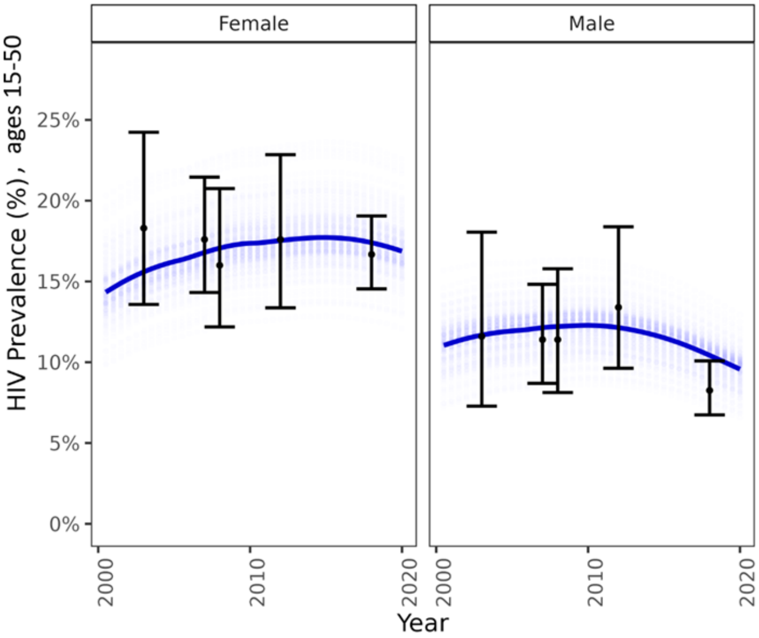

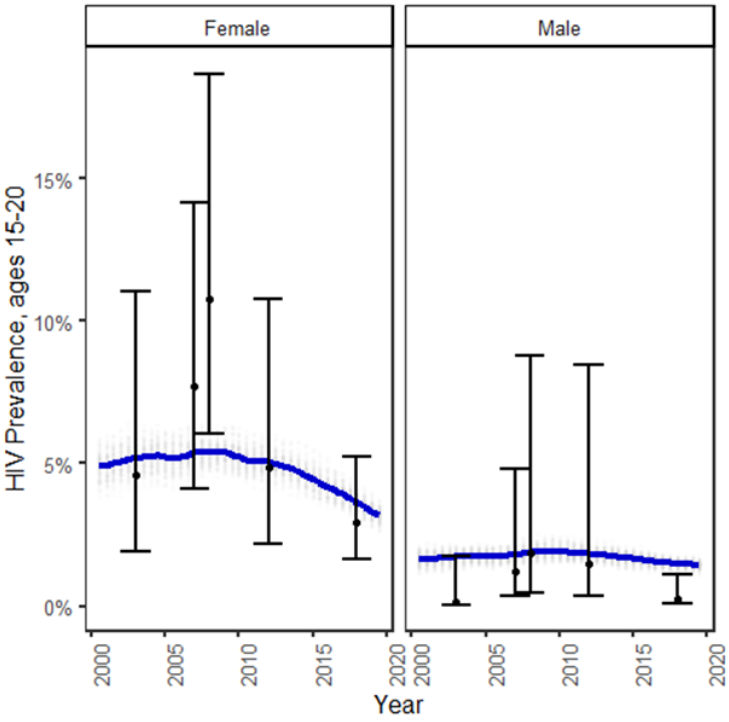
Female
Male
HIV Prevalence, ages 15-20
15%
10%
5%
0%
2000
2005
2010
2015
2020
Year

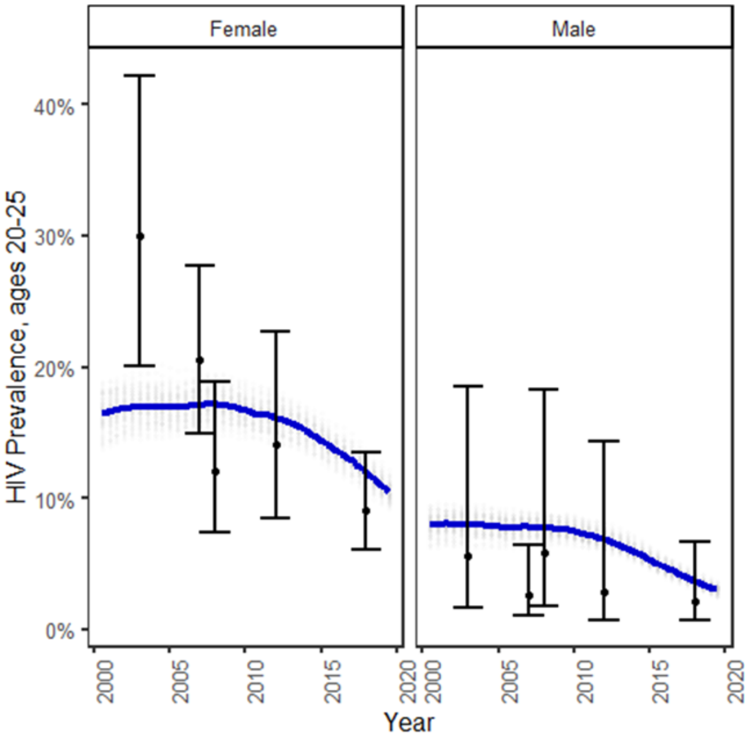
Female
Male
HIV Prevalence, ages 20-25
40%
30%
20%
10%
0%
2000
2005
2010
2015
2020
Year

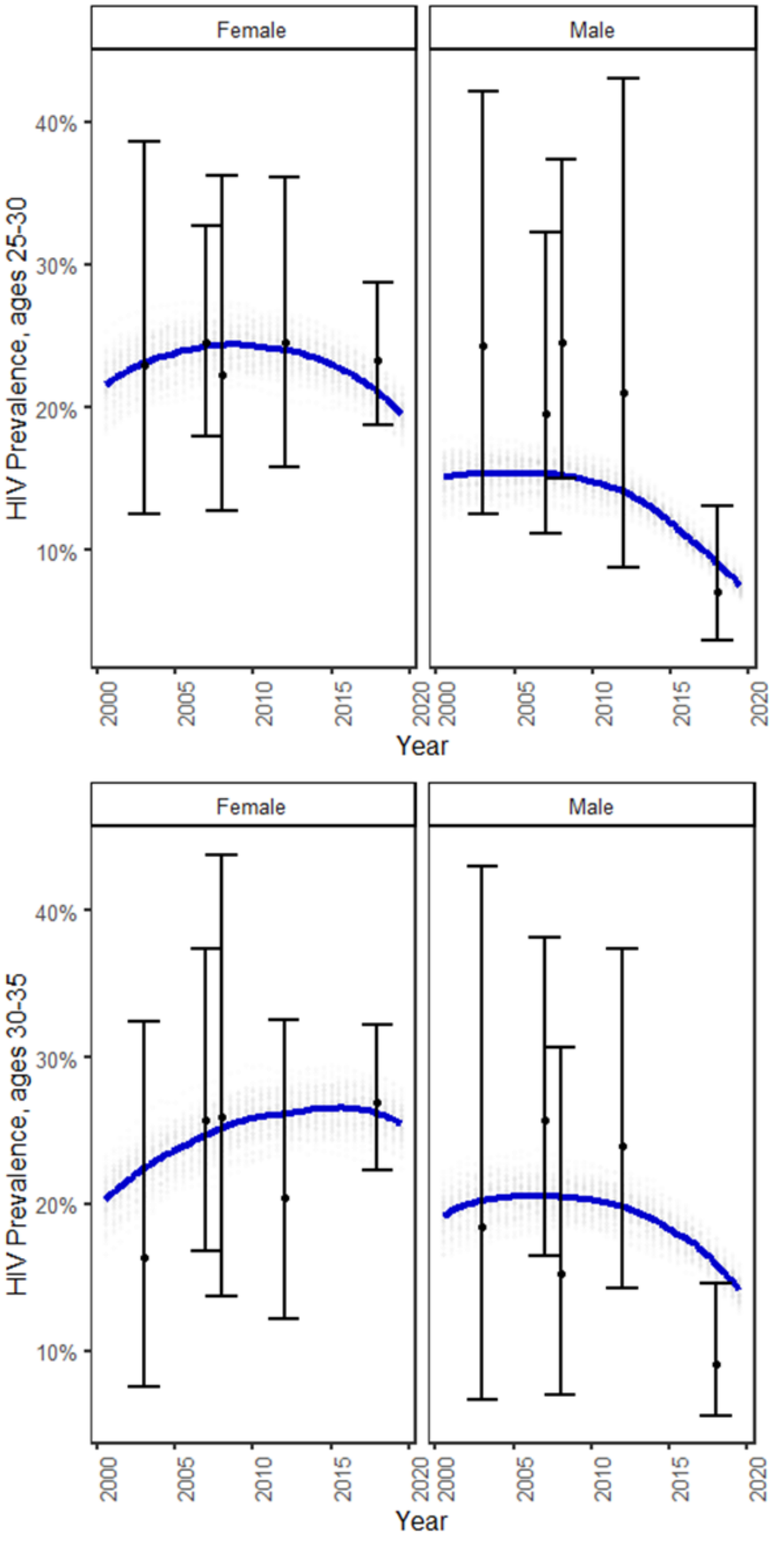
Female
Male
HIV Prevalence, ages 25-30
40%
30%
20%
10%
2000
2005
2010
2015
2020
Year
Female
Male
HIV Prevalence, ages 30-35
40%
30%
20%
10%
2000
2005
2010
2015
2020
Year

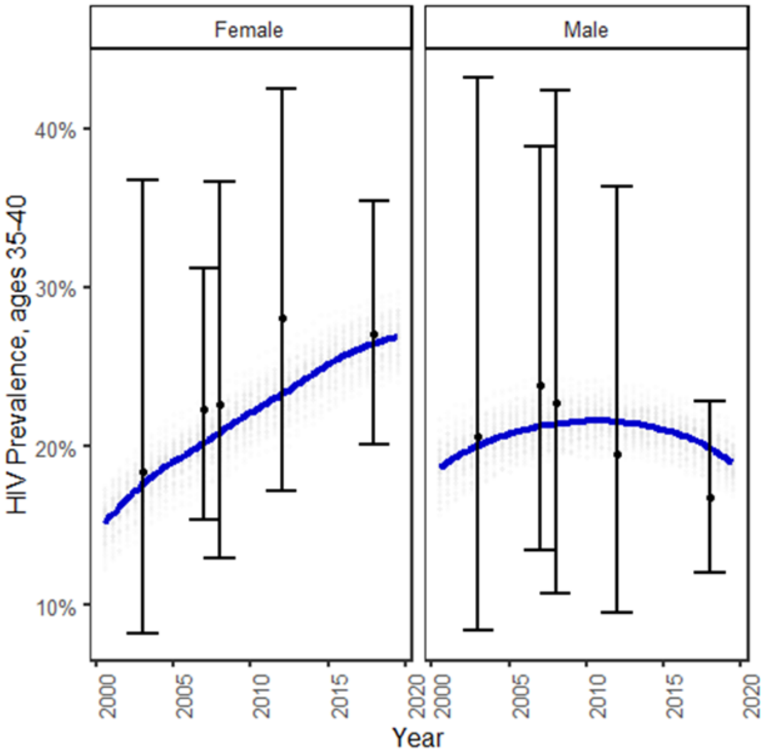
Female
Male
HIV Prevalence, ages 35-40
40%
30%
20%
10%
2000
2005
2010
2015
2020
Year

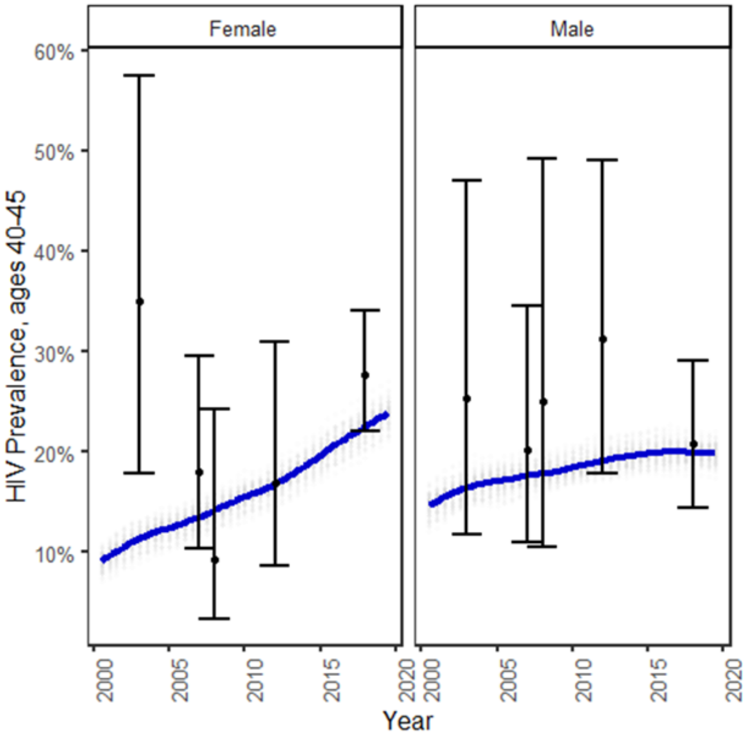
Female
Male
HIV Prevalence, ages 40-45
60%
50%
40%
30%
20%
10%
2000
2005
2010
2015
2020
Year

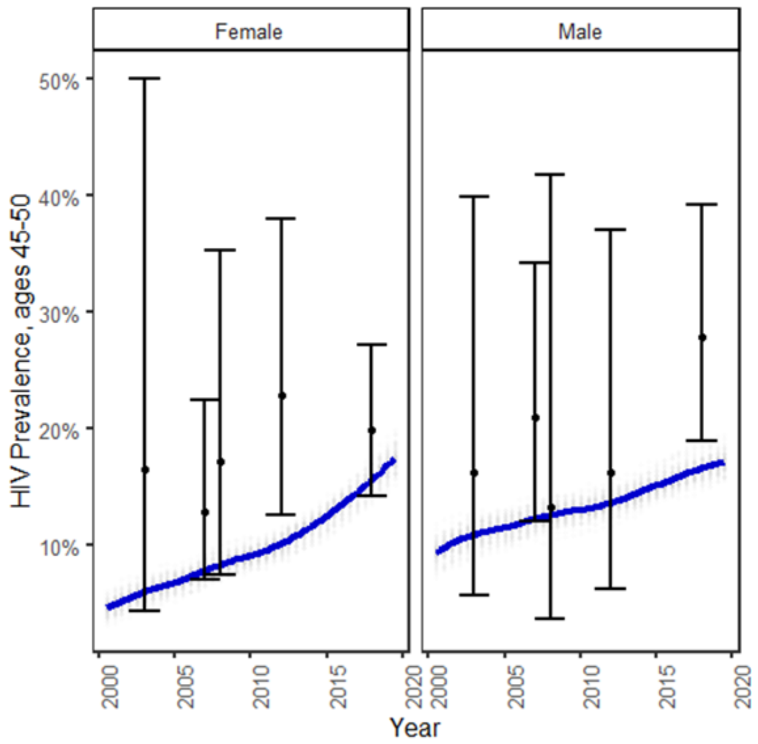

* Blue curves refer to the average over the 100 model simulations; the error bars refer to the empirical estimates and 95% confidence intervals for HIV prevalence obtained from Kenya Demographic and Health Surveys and Kenya AIDS Indicator Surveys; 95% confidence intervals were calculated using DHS survey weights and accounting for strata.

Figure S1b Model fit to age-specific and overall prevalence from population-based surveys by sex in South Africa

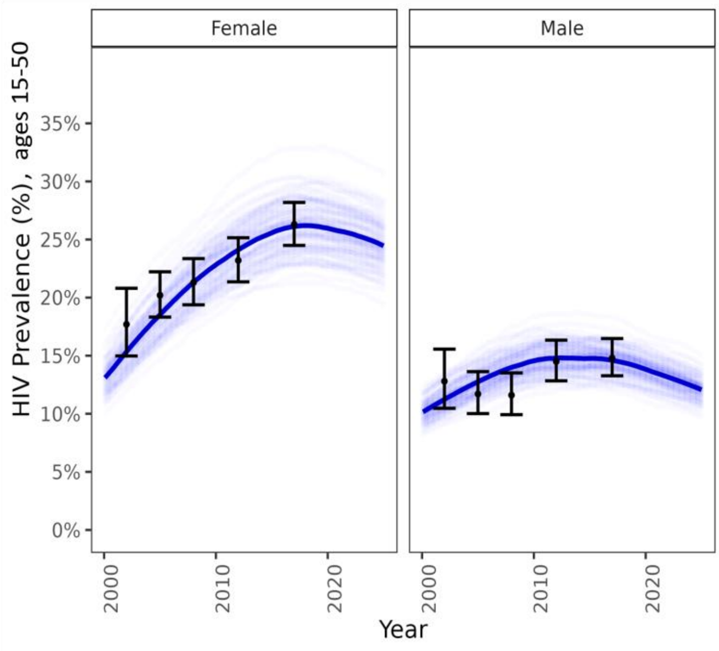

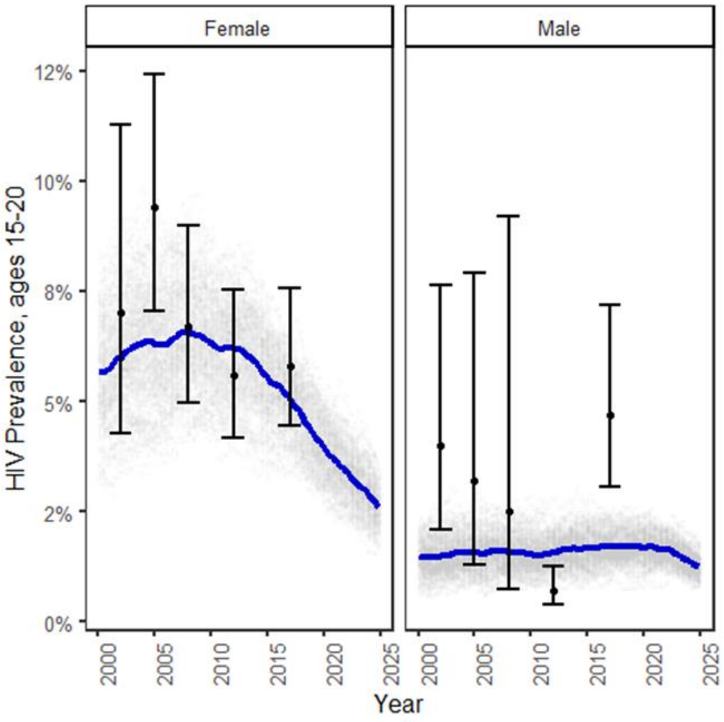
Female
Male
HIV Prevalence, ages 15-20
0%
2%
5%
8%
10%
12%
2000
2005
2010
2015
2020
2025
Year

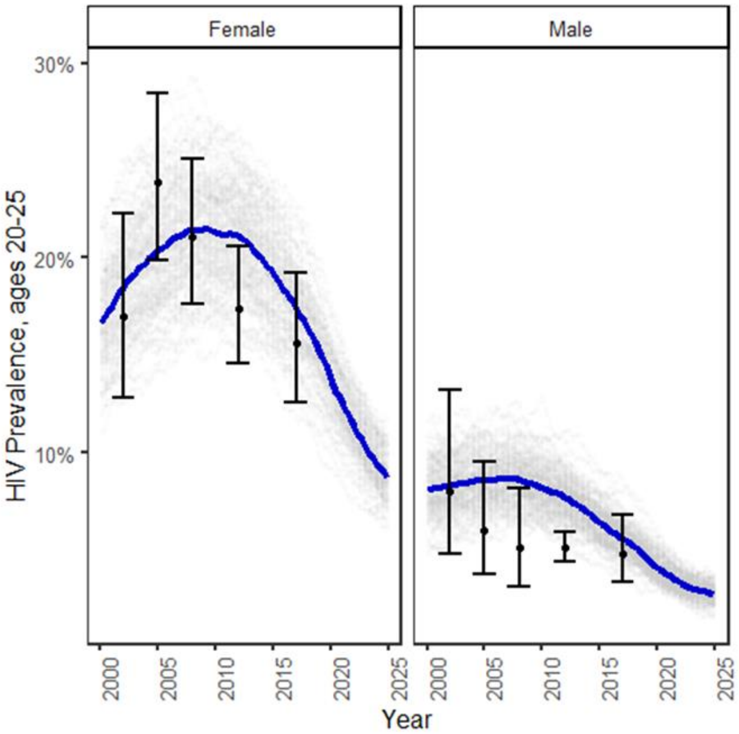
Female
Male
HIV Prevalence, ages 20-25
10%
20%
30%
2000
2005
2010
2015
2020
2025
Year

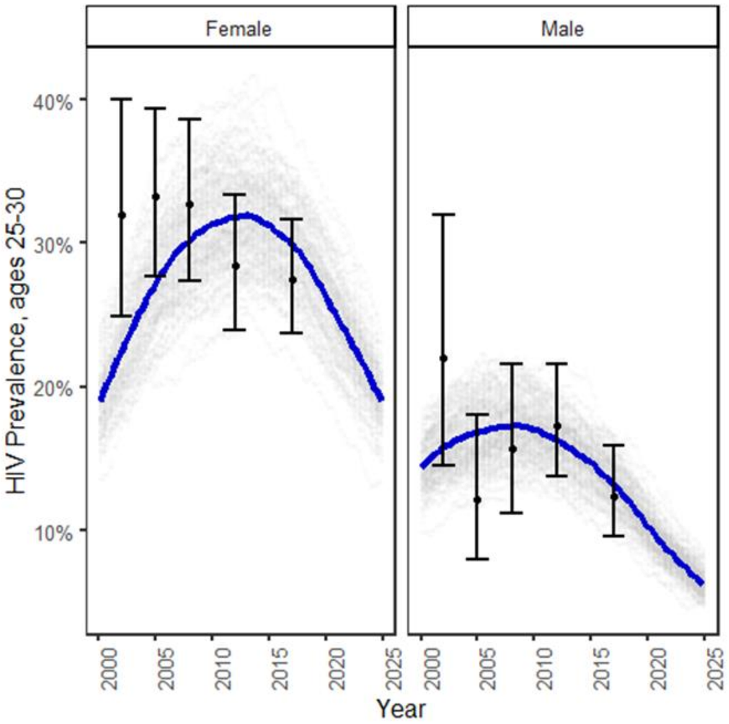

Female
Male
HIV Prevalence, ages 25-30
40%
30%
20%
10%
2000
2005
2010
2015
2020
2025
Year

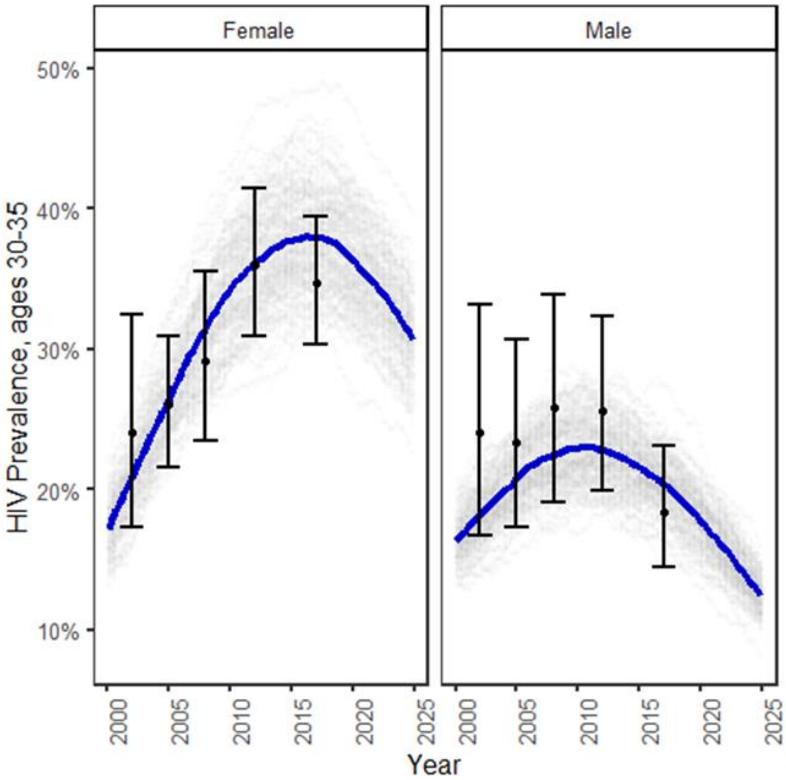

Female
Male
HIV Prevalence, ages 30-35
50%
40%
30%
20%
10%
2000
2005
2010
2015
2020
2025
Year

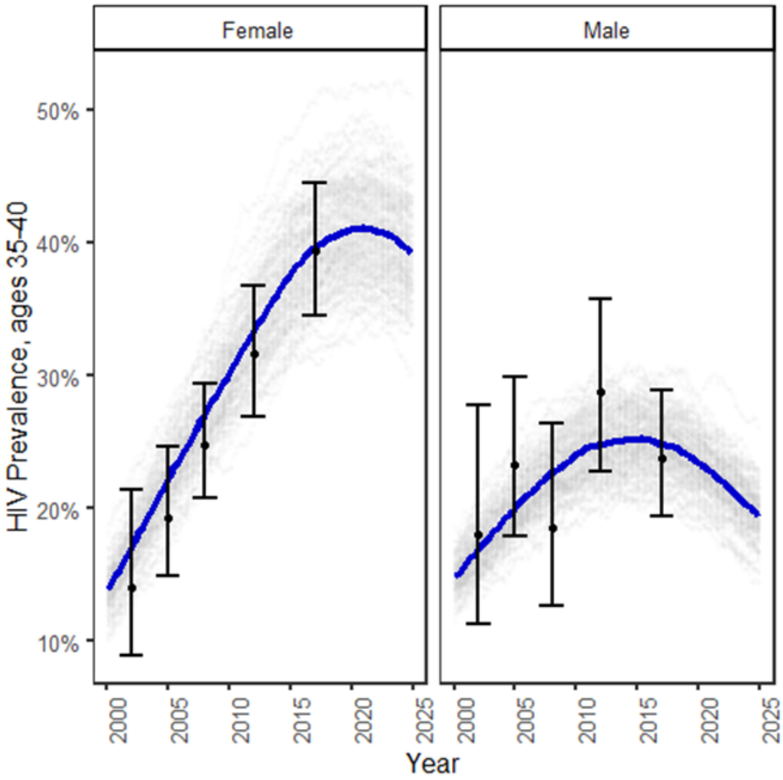
Female
Male
HIV Prevalence, ages 35-40
50%
40%
30%
20%
10%
2000
2005
2010
2015
2020
2025
Year

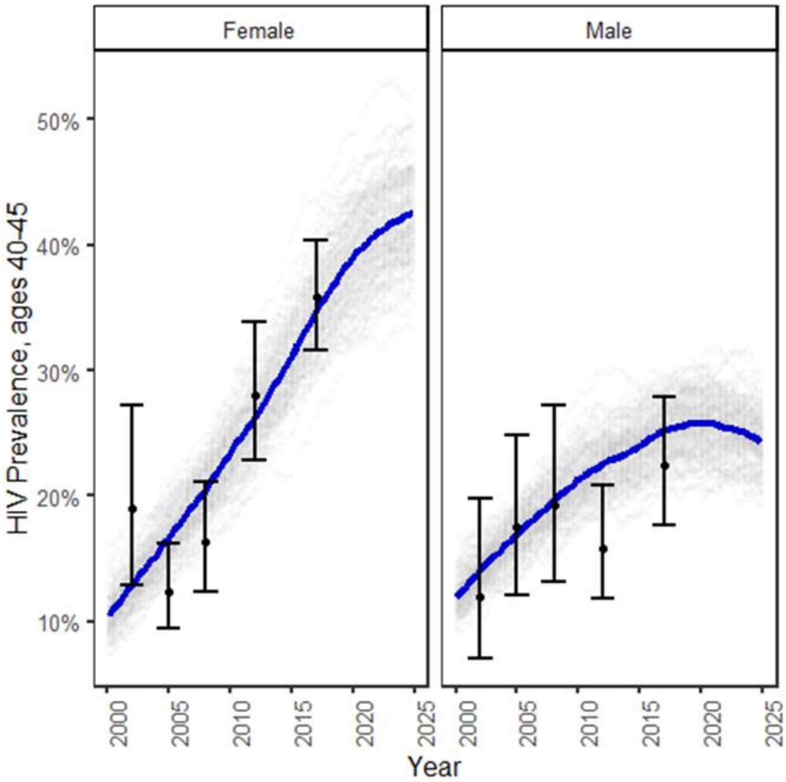
Female
Male
HIV Prevalence, ages 40-45
50%
40%
30%
20%
10%
2000
2005
2010
2015
2020
2025
Year

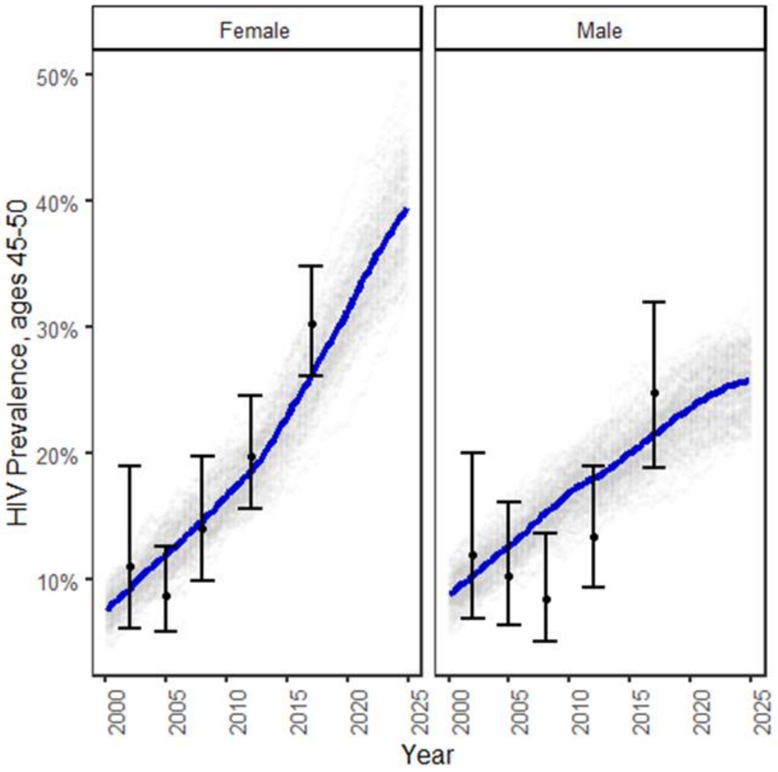

* Blue curves refer to the average over the 100 model simulations;; the error bars refer to the empirical estimates and 95% confidence intervals for HIV prevalence obtained from South African National HIV Prevalence, Incidence and Behaviour Surveys (2002, 2005, 2008, 2012 and 2017) from the Human Sciences Research Council (HSRC)

Figure S1c. Model fit to age-specific and overall prevalence from population-based surveys by sex in Zimbabwe

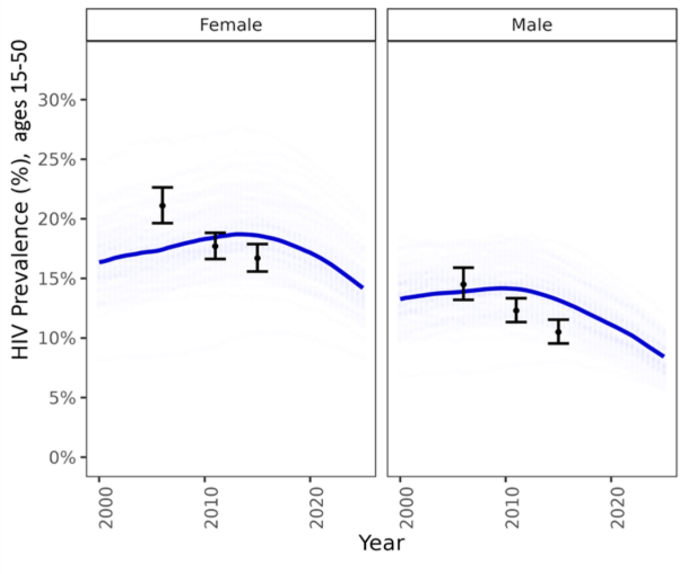

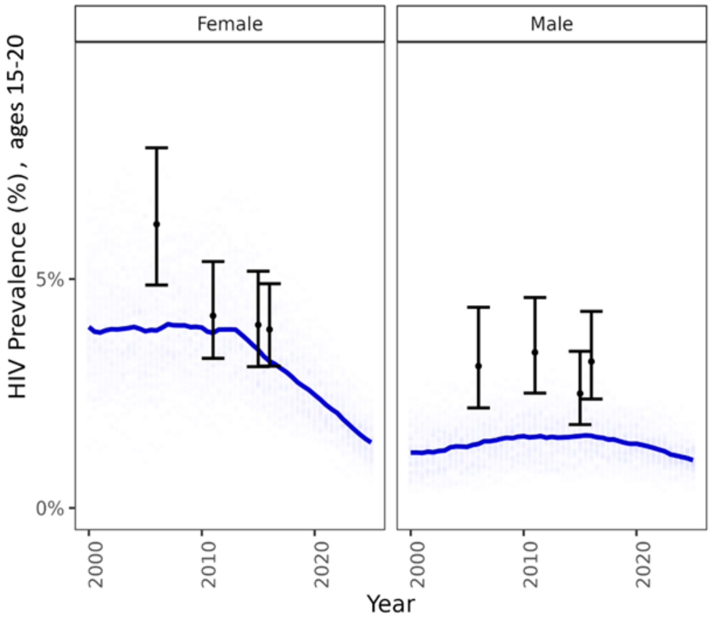
Female
Male
HIV Prevalence (%) , ages 15-20
5%
0%
2000
2010
2020
Year

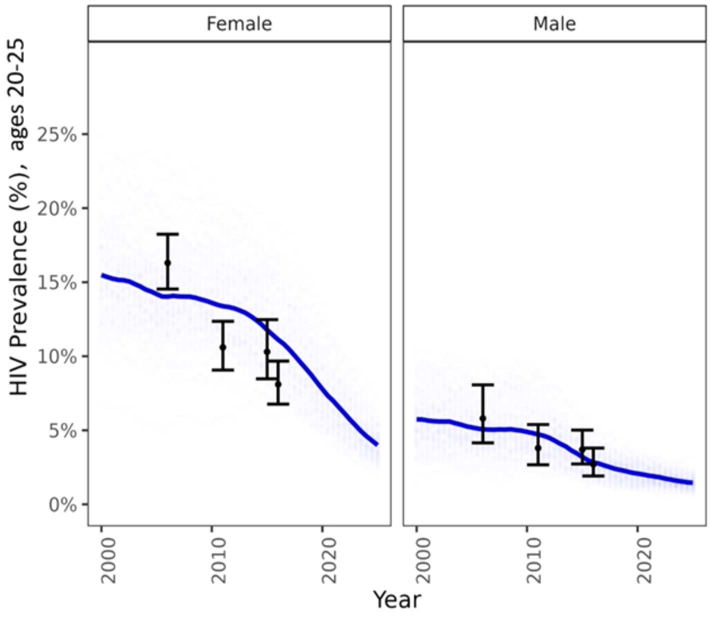
Female
Male
HIV Prevalence (%), ages 20-25
25%
20%
15%
10%
5%
0%
2000
2010
2020
Year

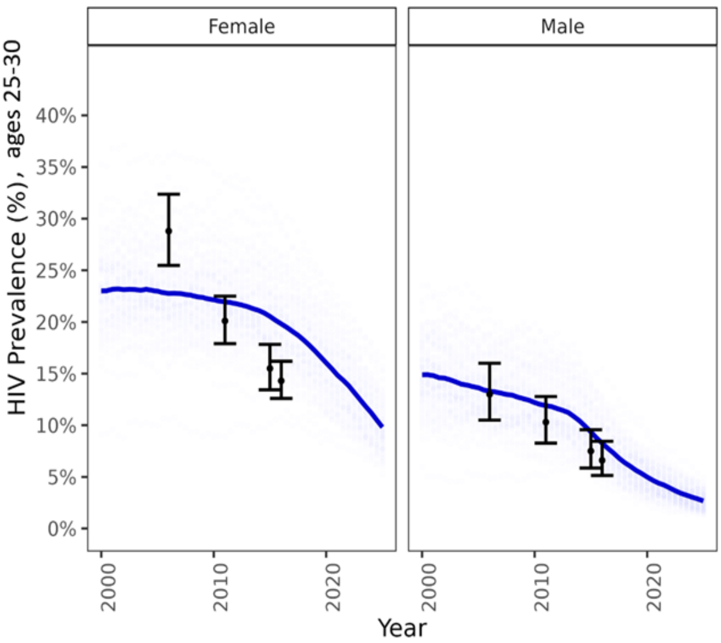

Female
Male
HIV Prevalence (%), ages 25-30
40%
35%
30%
25%
20%
15%
10%
5%
0%
2000
2010
2020
Year

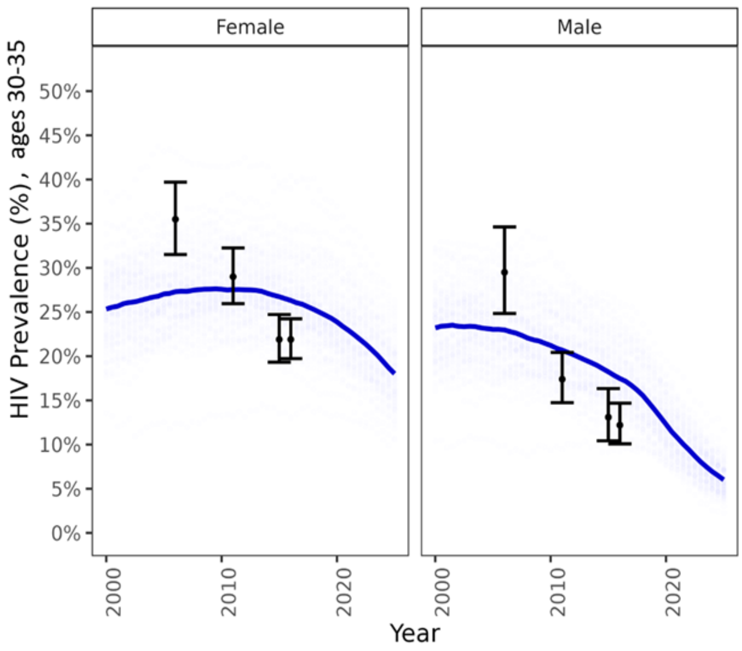

Female
Male
HIV Prevalence (%), ages 30-35
50%
45%
40%
35%
30%
25%
20%
15%
10%
5%
0%
2000
2010
2020
Year

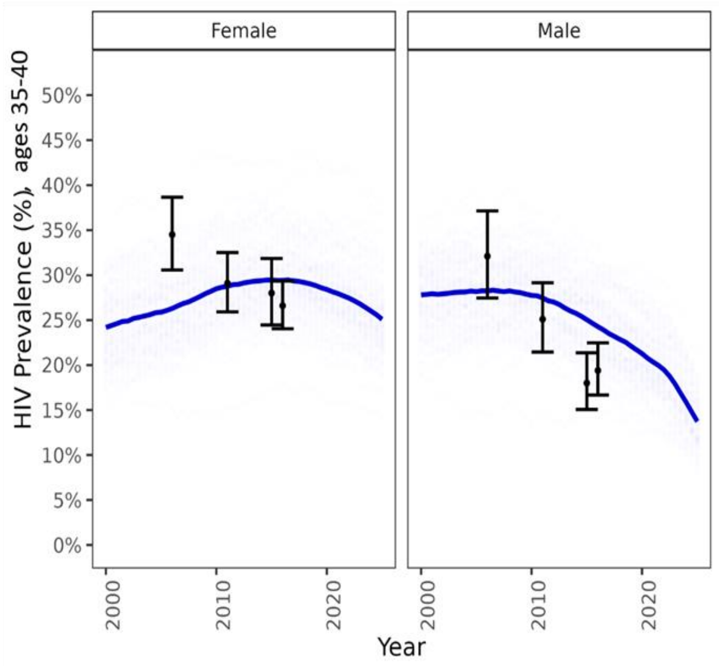
Female
Male
HIV Prevalence (%), ages 35-40
50%
45%
40%
35%
30%
25%
20%
15%
10%
5%
0%
2000
2010
2020
Year

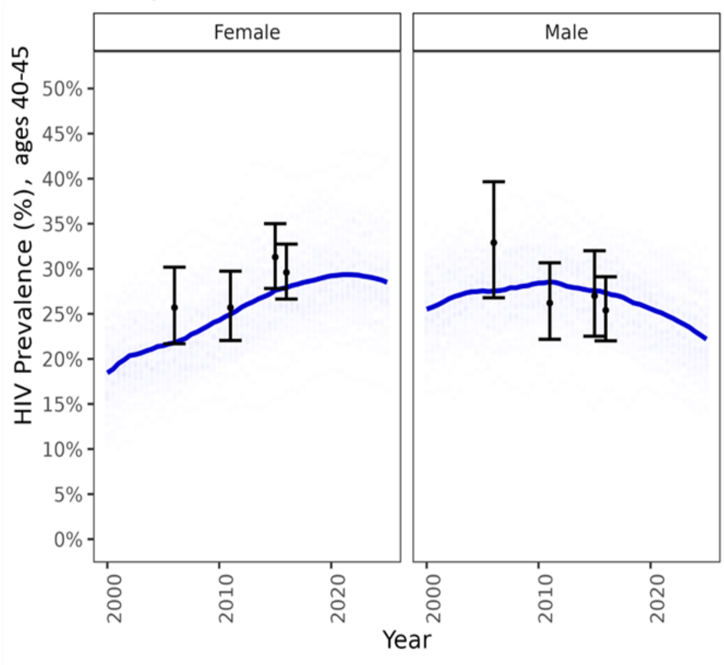
Female
Male
HIV Prevalence (%), ages 40-45
50%
45%
40%
35%
30%
25%
20%
15%
10%
5%
0%
2000
2010
2020
Year

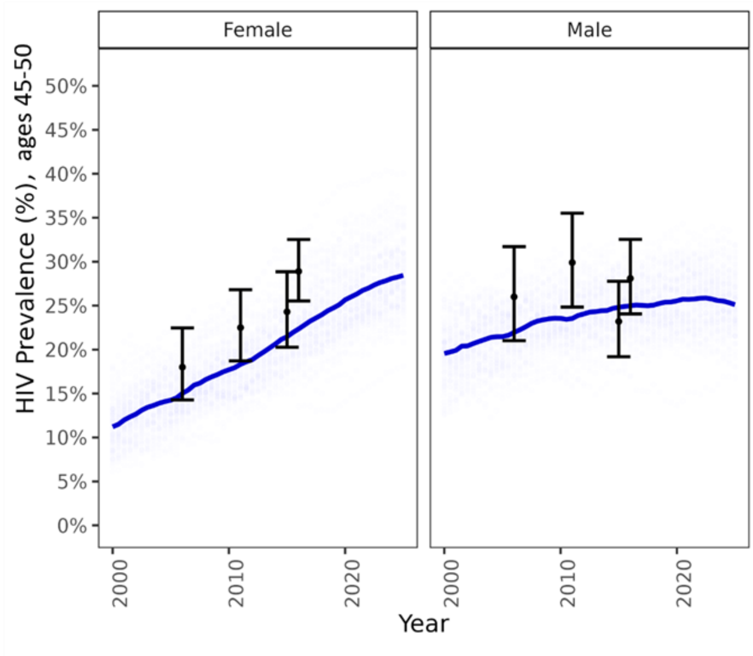

* Blue curves refer to the average over the 100 model simulations; the error bars refer to the empirical estimates and 95% confidence intervals for HIV prevalence obtained from PHIA Surveys

Model fit to age-specific and overall prevalence from population-based surveys by sex

Figure S2a Model fit to age-specific and overall ART coverage from population-based surveys by sex in Kenya*

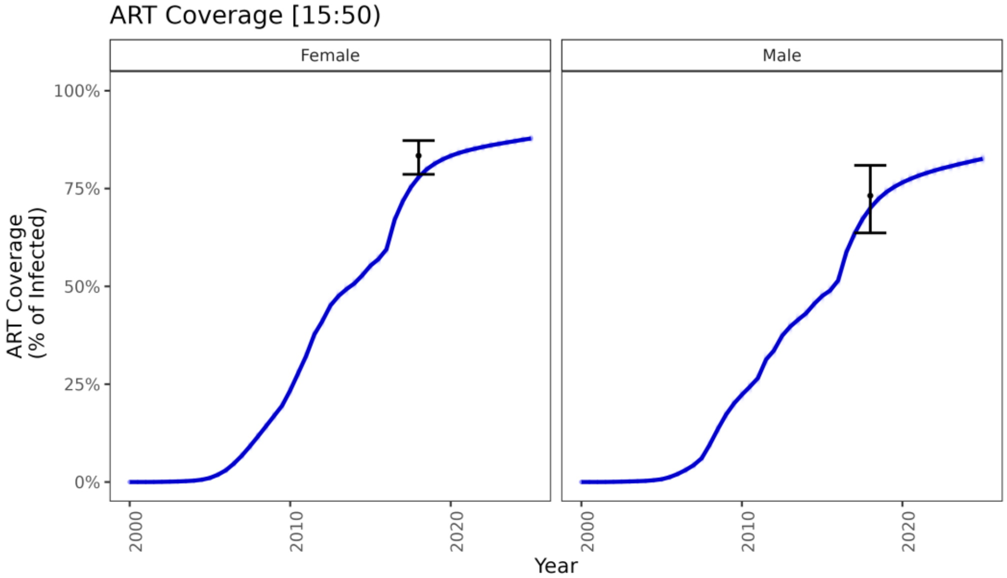

* There is a lack of data on historic ART coverage but the model fits well to the empiric data point and is calibrated to number of people on ART over time by sex, which provides confidence about historical fit.

Figure S2b Model fit to age-specific and overall ART coverage from population-based surveys by sex in South Africa

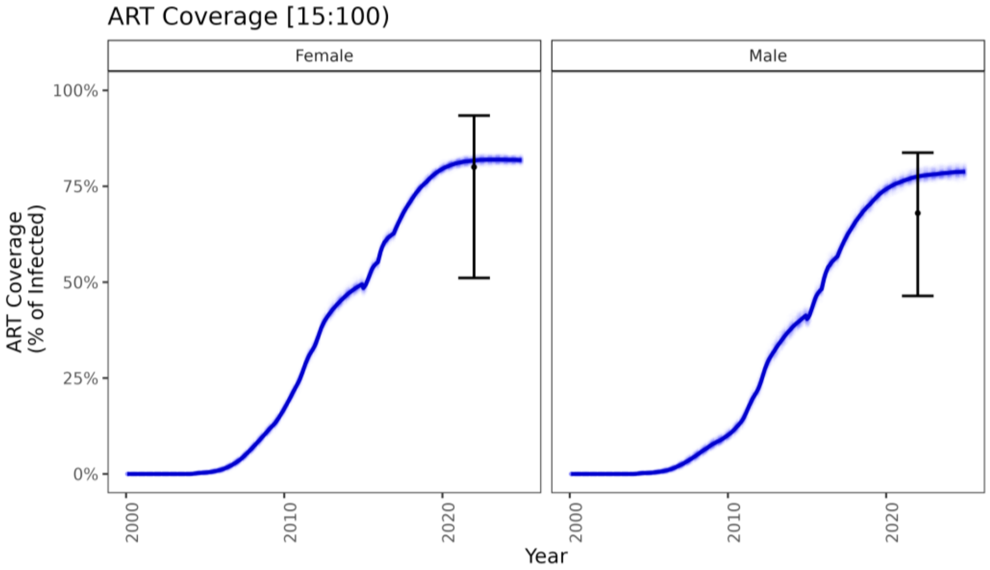

* The model fits well to the empiric data point for current coverage and is calibrated to number of people on ART over time by sex, which provides confidence about historical fit.

Figure S2c Model fit to age-specific and overall ART coverage from population-based surveys by sex in Zimbabwe

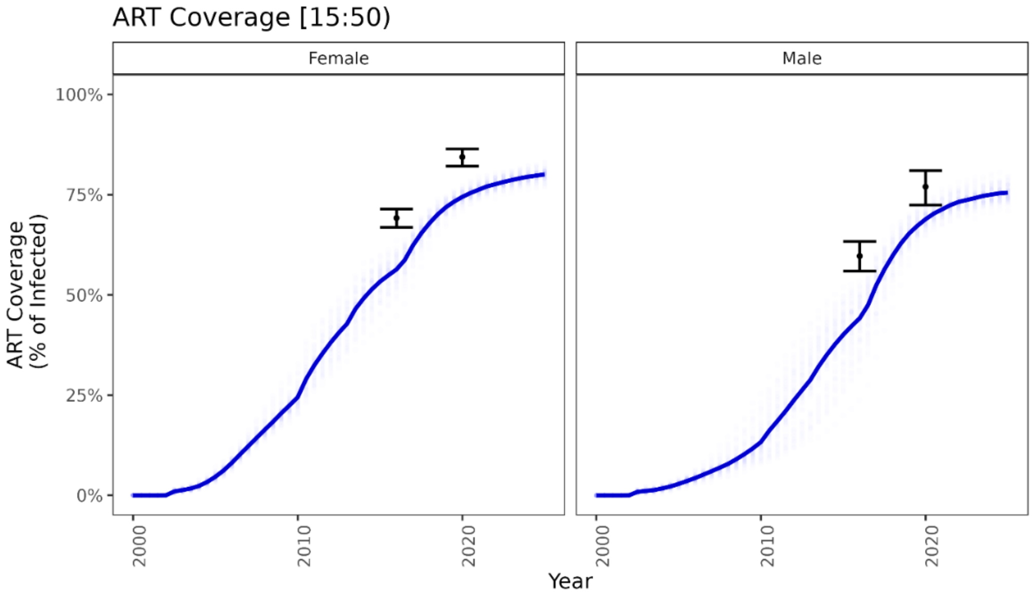

Model Overview and Parameters

Model initialization
The simulations begin prior to the start of infection in the year 1960 to allow sufficient time for the epidemic to burn-in. During this time, individuals with demographic properties specified in the demographics file begin forming relationships; relationship formation rates for each gender and relationship type are updated daily using a relationship flow algorithm. Adjustment of pair formation entry rates is terminated at a specific timepoint (e.g., 1975) and the rates are fixed at that value for the remainder of the simulation. The age profile of the population is initialized using demographic data including population age distribution or age-specific fertility rates. A prior analysis of our model evaluated the age/sex pairings, partnership length and other sexual network characteristics and confirmed that these outputs reached equilibrium within 20 years, prior to the introduction of HIV into the model: https://ieeexplore.ieee.org/abstract/document/6426573. (25) HIV infections are seeded in 1980 and affect a certain proportion of the population based on the age and gender distribution reported from historical data. ART intervention is introduced in the year 2012. Eligible individuals enroll in ART based on historical eligibility criteria based on CD4 count, which changes over time based on WHO guidelines for ART initiation until the implementation of universal ART, which is assumed to remain the same until the end of the simulations (year 2050). Calibration and validation processes are performed to refine the initialization and ensure that the model aligns with observed HIV dynamics in the target population (26,27).

The following provides a more detailed description of the model initialization process:
https://docs.idmod.org/projects/emod-hiv/en/latest/sti-model-relationships.html. (26)

EMOD-HIV is open-source and publicly available online: https://docs.idmod.org/projects/emod-hiv/en/2.20_a/

Modelled time step
The model is implemented using a monthly time step, aggregating events and changes over the course of each month. Monthly updated information can then be ascertained regarding a range of activities and occurrences, including sexual mixing, relationship formation, stages of HIV infection, HIV testing and its results, and PrEP initiation and discontinuation.

PrEP cascade

HIV-negative individuals aged 18 to 49 years were eligible for PrEP if testing negative at the time of initiation or continuation, and if sexually active with at least one partner. PrEP discontinuation occurred if individuals are

lost to follow-up or no longer met the eligibility criteria (i.e., an individual turned 50 years old, all partnerships end, or tested HIV-positive). Individuals who stopped PrEP can re-start at any time if they meet the eligibility criteria (i.e. start a new partnership).

Model Parameters

Table S12a. Select model parameters used to fit the EMOD-HIV transmission model to survey data on prevalence and ART coverage from Kenya.

Parameter	Parameter Description	Fitted median	(IQR)
ARTLinkMax	Maximum probability of linkage to ART	0.999	(0.978, 1.000)
ARTLinkMid	Year of ART linkage (given eligibility), that is, time of the inflection point in the sigmoid trend.	2003.008	(2002.920, 2003.837)
AcuteDurationMonths	The time since infection, in months, over which the Acute_Stage_Infectivity_Multiplier is applied to coital acts occurring in that time period.	1.000	(1.000, 1.331)
CircumcisionReducedAcquire	The reduction of susceptibility to STI by voluntary male medical circumcision (VMMC).	0.600	(0.598, 0.600)
Homa_BayInfrmlCondomsMax	Maximum rate of condom use in informal relationships in Homa Bay	0.231	(0.230, 0.233)
Homa_BayLOWRisk	Proportion of the population that is low-risk in Homa Bay	0.557	(0.549, 0.559)
Homa_BayTrnsCondomsMax	Maximum rate of condom use in transitory relationships in Homa Bay	0.244	(0.232, 0.245)
InfmrlFormRate	Informal relationship formation rate	0.000	(0.000, 0.000)
InfrmlCondomMid	Year midpoint of logistic scale-up of condom use in informal relationships	1993.007	(1991.629, 1998.680)
InfrmlCondomRate	Rate of logistic scale-up of condom use in informal relationships	2.661	(1.953, 2.909)
InfrmlCondomsMax	Maximum rate of condom use in informal relationships	0.216	(0.215, 0.217)
InfrmlDurHet	Heterogeneity in duration of informal relationships	0.750	(0.750, 0.750)
KisiiInfrmlCondomsMax	Maximum rate of condom use in informal relationships in Kisii	0.231	(0.226, 0.232)
KisiiLOWRisk	Proportion of the population that is low-risk in Kisii	0.938	(0.935, 0.939)
KisiiTrnsCondomsMax	Maximum rate of condom use in transitory relationships in Kisii	0.375	(0.369, 0.376)
KisumuInfrmlCondomsMax	Maximum rate of condom use in informal relationships in Kisumu	0.182	(0.181, 0.183)
KisumuLOWRisk	Proportion of the population that is low-risk in Kisumu	0.764	(0.762, 0.765)
KisumuTrnsCondomsMax	Maximum rate of condom use in transitory relationships in Kisumu	0.338	(0.337, 0.347)
LogBaseInfectivity	The probability of transmission when none of the transmission multipliers apply to a particular coital act.	0.002	(0.002, 0.002)
MaleToFemaleOld	Male-to-female relative risk of infection among older individuals	2.012	(1.506, 2.132)
MaleToFemaleYoung	Male-to-female relative risk of infection among young individuals	1.116	(1.000, 1.242)
MaxInfrmlFLOW	Maximum number of informal relationships among low-risk females	1.311	(1.255, 1.315)
MaxInfrmlFMED	Maximum number of informal relationships among medium-risk females	2.050	(2.002, 2.351)
MaxInfrmlMLOW	Maximum number of informal relationships among low-risk males	1.165	(1.159, 1.203)
MaxInfrmlMMED	Maximum number of informal relationships among medium-risk males	2.530	(2.442, 2.735)
MaxMrtlFMED	Maximum number of marital relationship among medium-risk females	1.139	(1.136, 1.159)
MaxMrtlMMED	Maximum number of marital relationship among medium-risk males	1.302	(1.268, 1.316)
MaxTrnsFLOW	Maximum number of transitory relationships among low-risk females	1.599	(1.580, 1.625)
MaxTrnsFMED	Maximum number of transitory relationships among medium-risk females	2.943	(2.891, 3.000)
MaxTrnsMLOW	Maximum number of transitory relationships among low-risk males	1.599	(1.588, 1.677)

Parameter	Parameter Description	Fitted median	(IQR)
MaxTrnsMMED	Maximum number of transitory relationships among medium-risk males	2.557	(2.475, 2.879)
MigoriInfrmlCondomsMax	Maximum rate of condom use in informal relationships in Migori	0.186	(0.185, 0.189)
MigoriLOWRisk	Proportion of the population that is low-risk in Migori	0.795	(0.793, 0.797)
MigoriTrnsCondomsMax	Maximum rate of condom use in transitory relationships in Migori	0.249	(0.248, 0.257)
MrtlCondomMax	Maximum rate of condom use in marital relationships	0.192	(0.191, 0.193)
MrtlCondomMid	Year midpoint of logistic scale-up of condom use in marital relationships	2003.131	(1999.017, 2005.000)
MrtlCondomRate	Rate of logistic scale-up of condom use in marital relationships	2.971	(2.595, 3.000)
MrtlFormRate	Marital relationship formation rate	0.000	(0.000, 0.000)
NyamiraInfrmlCondomsMax	Maximum rate of condom use in informal relationships in Nyamira	0.096	(0.093, 0.097)
NyamiraLOWRisk	Proportion of the population that is low-risk in Nyamira	0.902	(0.900, 0.910)
NyamiraTrnsCondomsMax	Maximum rate of condom use in transitory relationships in Nyamira	0.309	(0.304, 0.313)
PrExInfrmlFemLOW	Probability of potential for extra-relational informal relationship among low-risk females	0.366	(0.364, 0.371)
PrExInfrmlFemMED	Probability of potential for extra-relational informal relationship among medium-risk females	0.401	(0.401, 0.407)
PrExInfrmlMaleLOW	Probability of potential for extra-relational informal relationship among low-risk males	0.244	(0.222, 0.249)
PrExInfrmlMaleMED	Probability of potential for extra-relational informal relationship among medium-risk males	0.382	(0.379, 0.389)
PrExTrnsFemLOW	Probability of potential for extra-relational transitory relationship among low-risk females	0.033	(0.033, 0.033)
PrExTrnsFemMED	Probability of potential for extra-relational transitory relationship among medium-risk females	0.460	(0.450, 0.468)
PrExTrnsMaleLOW	Probability of potential for extra-relational transitory relationship among low-risk males	0.330	(0.328, 0.332)
PrExTrnsMaleMED	Probability of potential for extra-relational transitory relationship among medium-risk males	0.593	(0.589, 0.611)
PreARTLinkMax	Maximum probability of linkage to pre-ART care	0.715	(0.708, 0.737)
PreARTLinkMid	Year midpoint of logistic scale-up of pre-ART linkage	1996.709	(1995.685, 1998.896)
PreARTLinkMin	Minimum probability of linkage to pre-ART care	0.435	(0.416, 0.442)
RiskAssortivity	Risk assortivity	0.663	(0.648, 0.673)
SeedYrHigh	Seed year	1986.562	(1982.000, 1988.000)
SexualDebutAgeFemaleWeibullHeterogeneity	Heterogeneity parameter of Weibull distribution of female age of sexual debut	0.086	(0.083, 0.086)
SexualDebutAgeFemaleWeibullScale	Scale parameter of Weibull distribution of female age of sexual debut	16.013	(15.540, 16.038)
SexualDebutAgeMaleWeibullHeterogeneity	Heterogeneity parameter of Weibull distribution of male age of sexual debut	0.040	(0.040, 0.040)
SexualDebutAgeMaleWeibullScale	Scale parameter of Weibull distribution of male age of sexual debut	15.708	(15.155, 16.090)
SiayaInfrmlCondomsMax	Maximum rate of condom use in informal relationships in Siaya	0.160	(0.148, 0.161)
SiayaLOWRisk	Proportion of the population that is low-risk in Siaya	0.729	(0.722, 0.731)
SiayaTrnsCondomsMax	Maximum rate of condom use in transitory relationships in Siaya	0.300	(0.293, 0.304)
TrnsCondomMax	Maximum rate of condom use in transitory relationships	0.243	(0.242, 0.253)
TrnsCondomMid	Year midpoint of logistic scale-up of condom use in transitory relationships	1997.960	(1996.962, 1999.273)
TrnsCondomRate	Rate of logistic scale-up of condom use in transitory relationships	0.999	(0.978, 1.000)
TrnsFormRate	Transitory relationship formation rate	2003.008	(2002.920, 2003.837)

* Median and interquartile ranges (IQRs) reported for all dynamic parameters used in the calibration process from 100 best-fitting parameter sets. †

Table S12b. Select model parameters used to fit the EMOD-HIV transmission model to survey data on prevalence and ART coverage from South Africa.

Parameter	Description	Fitted Median	(IQR)
ART Link Max	Maximum probability of linkage to ART	1.000	(0.997, 1.000)
ART Link Mid	Year of ART linkage (given eligibility), that is, time of the inflection point in the sigmoid trend.	2,005.96	(2,005.86, 2,006.08)
All: Infmrl Condom	Modern condom usage rate in informal relationships across all locations	0.61	(0.58, 0.64)
All: LOW Risk	Proportion of the population that is low-risk	0.936	(0.930, 0.942)
All: Trns Condom	Modern condom usage rate in transitory relationships across all locations	0.2	(0.16, 0.23)
Base Infectivity	The probability of transmission when none of the transmission multipliers apply to a coital act (or when all multipliers are set to 1).	0.0015	(0.0015, 0.0016)
Circumcision Reduced Acquire	The reduction of susceptibility to STI by voluntary male medical circumcision (VMMC).	0.6	
Infmrl Condoms Late	Modern condom usage rate in informal relationships, by county	0.37	(0.35, 0.39)
Infmrl Form Rate	Informal relationship formation rate	0.0009	(0.0008, 0.0009)
Infrml Condom Mid	Year midpoint of logistic scale-up of condom use in informal relationships	1,998.27	(1,997.81, 1,999.03)
Infrml Condom Rate	Rate of logistic scale-up of condom use in informal relationships	2.03	(1.89, 2.18)
Infrml Dur Het	Heterogeneity in duration of informal relationships	0.693	(0.675, 0.711)
Male To Female Old	Male-to-female relative risk of infection among older individuals	2.33	(2.22, 2.49)
Male To Female Young	Male-to-female relative risk of infection among young individuals	2.97	(2.74, 3.37)
Max Infmrl F LOW	Maximum number of informal relationships among low-risk females	1.67	(1.61, 1.72)
Max Infmrl F MED	Maximum number of informal relationships among medium-risk females	0.92	(0.89, 0.94)
Max Infmrl M LOW	Maximum number of informal relationships among low-risk males	1.73	(1.69, 1.76)
Max Infmrl M MED	Maximum number of informal relationships among medium-risk males	0.75	(0.70, 0.79)
Max Mrtl F ME	Maximum number of marital relationships among medium-risk females	1.23	(1.16, 1.27)
Max Mrtl M ME	Maximum number of marital relationships among medium-risk males	0.93	(0.91, 0.96)
Max Trns F LOW	Maximum number of transitory relationships among low-risk females	1.5	(1.48, 1.52)
Max Trns F MED	Maximum number of transitory relationships among medium-risk females	3.1	(3.06, 3.14)
Max Trns M LOW	Maximum number of transitory relationships among low-risk males	1.49	(1.46, 1.53)

Max Trns M MED	Maximum number of transitory relationships among medium-risk males	3.02	(2.88, 3.10)
Mrtl Condom Max	Maximum rate of condom use in marital relationships	0.191	(0.184, 0.211)
Mrtl Condom Mid	Year midpoint of logistic scale-up of condom use in marital relationships	1,994.92	(1,994.19, 1,995.27)
Mrtl Condom Rate	Rate of logistic scale-up of condom use in marital relationships	3.6	(3.49, 3.69)
Mrtl Form Rate	Marital relationship formation rate	0.0001	(0.0001, 0.0001)
Pr Ex Infmrl Fem LOW	Probability of potential for extra-relational informal relationship among low-risk females	0.08	(0.06, 0.10)
Pr Ex Infmrl Fem MED	Probability of potential for extra-relational informal relationship among medium-risk females	0.391	(0.381, 0.404)
Pr Ex Infmrl Male LOW	Probability of potential for extra-relational informal relationship among low-risk males	0.46	(0.42, 0.50)
Pr Ex Infmrl Male MED	Probability of potential for extra-relational informal relationship among medium-risk males	0.379	(0.370, 0.390)
Pr Ex Trns Fem LOW	Probability of potential for extra-relational transitory relationship among low-risk females	0.066	(0.058, 0.081)
Pr Ex Trns Fem MED	Probability of potential for extra-relational transitory relationship among medium-risk females	0.58	(0.56, 0.62)
Pr Ex Trns Male LOW	Probability of potential for extra-relational transitory relationship among low-risk males	0.17	(0.12, 0.19)
Pr Ex Trns Male MED	Probability of potential for extra-relational transitory relationship among medium-risk males	0.59	(0.58, 0.62)
PreART Link Max	Maximum probability of linkage to pre-ART care	0.89	(0.85, 0.96)
PreART Link Mid	Year midpoint of logistic scale-up of pre-ART linkage	1,998.28	(1,997.76, 1,998.72)
PreART Link Min	Minimum probability of linkage to pre-ART care	0.63	(0.62, 0.65)
Risk Assortivity	Risk assortivity	0.47	(0.44, 0.49)
SeedYr HIGH	Seed year	1991	
Sexual Debut Age Female Weibull Heterogeneity	Heterogeneity parameter of Weibull distribution of female age of sexual debut	0.062	(0.051, 0.067)
Sexual Debut Age Female Weibull Scale	Scale parameter of Weibull distribution of female age of sexual debut	16.49	(16.34, 16.62)
Sexual Debut Age Male Weibull Heterogeneity	Heterogeneity parameter of Weibull distribution of male age of sexual debut	0.043	(0.038, 0.050)
Sexual Debut Age Male Weibull Scale	Scale parameter of Weibull distribution of male age of sexual debut	16.47	(16.24, 16.62)
Trns Condom Late	Modern condom usage rate in transitory relationships	0.61	(0.59, 0.64)
Trns Condom Mid	Year midpoint of logistic scale-up of condom use in transitory relationships	2,006.77	(2,006.08, 2,007.21)
Trns Condom Rate	Rate of logistic scale-up of condom use in transitory relationships	1.94	(1.84, 2.01)

Trns Form Rate	Transitory relationship formation rate	0.0013	(0.0013, 0.0014)

* Median and interquartile ranges (IQRs) reported for all dynamic parameters used in the calibration process from 100 best-fitting parameter sets. †

Table S12c. Select model parameters used to fit the EMOD-HIV transmission model to survey data on prevalence and ART coverage from Zimbabwe.

Parameter	Parameter Description	Fitted Median	(IQR)
All: LOW Risk	Proportion of the population that is low-risk	0.523	(0.500, 0.540)
Base Infectivity	The probability of transmission when none of the transmission multipliers apply to a coital act	0.0038	(0.0035, 0.0040)
Demographic Coverage	Demographic coverage of the intervention	0.193	(0.185, 0.205)
HCT Uptake Post Debut Max	Maximum rate of HIV counseling and testing (HCT) post sexual debut	0.34	(0.28, 0.38)
HCT Uptake Post Debut Mid	Year midpoint of logistic scale-up of HIV counseling and testing (HCT) post sexual debut	2,008.86	(2,008.32, 2,009.70)
Informal Form Rate	Informal relationship formation rate	0.0012	(0.0011, 0.0013)
Infrml Condom Mid	Year midpoint of logistic scale-up of condom use in informal relationships	1,998.13	(1,997.41, 1,998.67)
Infrml Condom Rate	Rate of logistic scale-up of condom use in informal relationships	3.85	(3.73, 4.00)
Infrml Condoms Max	Maximum rate of condom use in informal relationships	0.21	(0.19, 0.26)
Male To Female Old	Male-to-female relative risk of infection among older individuals	1.44	(1.33, 1.66)
Male To Female Young	Male-to-female relative risk of infection among young individuals	2.66	(2.26, 2.83)
Marital Form Rate	Marital relationship formation rate	0.00011	(0.00009, 0.00014)
Max Infrml F LOW	Maximum number of informal relationships among low-risk females	1.25	(1.18, 1.32)
Max Infrml F MED	Maximum number of informal relationships among medium-risk females	2.83	(2.35, 3.15)
Max Infrml M LOW	Maximum number of informal relationships among low-risk males	1.17	(1.13, 1.21)
Max Infrml M MED	Maximum number of informal relationships among medium-risk males	2.27	(2.13, 2.46)
Max Mrtl F MED	Maximum number of marital relationships among medium-risk females	1.18	(1.12, 1.24)
Max Mrtl M MED	Maximum number of marital relationships among medium-risk males	0.93	(0.90, 0.98)
Max Trns F LOW	Maximum number of transitory relationships among low-risk females	1.7	(1.65, 1.73)
Max Trns F MED	Maximum number of transitory relationships among medium-risk females	2.78	(2.55, 3.03)
Max Trns M LOW	Maximum number of transitory relationships among low-risk males	1.95	(1.91, 2.00)
Max Trns M MED	Maximum number of transitory relationships among medium-risk males	2.52	(2.41, 2.60)
Mrtl Condom Max	Maximum rate of condom use in marital relationships	0.18	(0.18, 0.21)

Mrtl Condom Mid	Year midpoint of logistic scale-up of condom use in marital relationships	2,000.76	(2,000.28, 2,002.48)
Mrtl Condom Rate	Rate of logistic scale-up of condom use in marital relationships	3.49	(3.20, 3.61)
Pr Ex Infrml Fem LOW	Probability of potential for extra-relational informal relationship among low-risk females	0.28	(0.26, 0.32)
Pr Ex Infrml Fem MED	Probability of potential for extra-relational informal relationship among medium-risk females	0.34	(0.24, 0.37)
Pr Ex Infrml Male LOW	Probability of potential for extra-relational informal relationship among low-risk males	0.49	(0.46, 0.56)
Pr Ex Infrml Male MED	Probability of potential for extra-relational informal relationship among medium-risk males	0.24	(0.20, 0.28)
Pr Ex Trns Fem LOW	Probability of potential for extra-relational transitory relationship among low-risk females	0.098	(0.086, 0.118)
Pr Ex Trns Fem MED	Probability of potential for extra-relational transitory relationship among medium-risk females	0.72	(0.66, 0.78)
Pr Ex Trns Male LOW	Probability of potential for extra-relational transitory relationship among low-risk males	0.2	(0.14, 0.23)
Pr Ex Trns Male MED	Probability of potential for extra-relational transitory relationship among medium-risk males	0.69	(0.68, 0.75)
Risk Assortivity	Risk assortivity	0.62	(0.61, 0.65)
SeedYr HIGH	Seed year	1,976.87	(1,975.63, 1,977.77)
Trans Form Rate	Transitory relationship formation rate	0.0003	(0.0001, 0.0004)
Trns Condom Mid	Year midpoint of logistic scale-up of condom use in transitory relationships	2,001.80	(1,999.58, 2,003.89)
Trns Condom Rate	Rate of logistic scale-up of condom use in transitory relationships	3.23	(2.88, 3.48)
Trns Condoms Max	Maximum rate of condom use in transitory relationships	0.22	(0.19, 0.25)

* Median and interquartile ranges (IQRs) reported for all dynamic parameters used in the calibration process from 100 best-fitting parameter sets. †

Table S13. Utility weights for estimating disability-adjusted life-years averted

Health State	DALY Weight	Reference
HIV-negative	0	Vos *et al (28)*
HIV and not on ART	0.274	
HIV and on ART	0.078	

Costing parameters

Table S14 Kenya costing parameter calculations

Cost parameter	Estimate (USD)	Year	Data source	Calculation detials, notes
**HIV costs**				
Annual health care costs (among those not on ART)				
HIV-positive CD4 < 200	110.3	2021	Eaton 2014 (29)	Adjusted for inflation and GDP/capita ratio using this approach: **Step 1:** Adjust South Africa value in 2012 USD for inflation to be in 2021 USD ($374.08 = $167*2.24) Cost of health care use, CD4 count <200 cells per µL, not in HIV care (per person-year) in South Africa= $167 USD Inflation Rate between time of costing (2012) and 2021: 2.24 =4.7/2.1 **Step 2:** Adjust South Africa 2021 USD value by multiplying by the Kenya GDP/cap ratio ($374.08*0.295) South Africa 2021 GDP per capita in $USD = 7,055; Kenya $USD = 2,082 Kenya GDP/ ZA GDP ratio adjustment: (2,082/7,055)= 0.295
HIV-positive CD4 200 - 349	30.38	2021	Eaton 2014 (29)	Adjusted for inflation and GDP/capita ratio (see steps above)
HIV-positive CD4 > 350	8.59	2021	Eaton 2014 (29)	Adjusted for inflation and GDP/capita ratio (see steps above)
End of life care	105.68	2021	Eaton 2014 (29)	Adjusted for inflation and GDP/capita ratio (see steps above)
Annual ART provision costs (30)	196.85	2020	Long et al. (2010) (30)	1st line ART delivery cost: $121 in 2016 USD → $131 in 2020 USD includes labs + staff encounters Cost of 1st line ART: $131 USD (delivery cost in 2020 USD) + $43.20 (ART)*1.2(additional 20% suplly chain)=$ 182.84; The delivery cost ratio between 2nd and 1st ART is 2.4; Cost of 2nd line ART: $131*2.4 USD (delivery cost in 2020 USD) + $279.60*1.2=$649.92; weighted average of 1st adn 2nd lines ART cost assuming 3% on 2nd line ART: $182.84*0.97 + $649.92*0.03=$196.85
**Oral PrEP costs**				
Oral PrEP per person month, facility	10.88	2019	Wanga et al. 2019 (31)	Data from micro-costing study, including viable (personnel, drug, lab and HIV testing, and other) and fixed (traning, demand creation, personnel, capital, overhead) costs
Facility-based HIV-positive test	3.68	2017	Meisner et al. (2021) (32)	Data from micro-costing study. Inputs include screening test kit, other supply costs, and personnel costs.
Facility-based HIV-negative test	2.64	2017	Meisner et al. (2021) (32)	Data from micro-costing study. Inputs include screening test kit, other supply costs, and personnel costs.

**Lenacapavir costs**				
Demand generation	10%			Assumption
Delivery	$8.55: $2.50 for HIV test, $0.05 for syringe, $6 for delivery overhead (facilities, staff, etc.)		Galactionova et al. (2015) (33)<br><br>Ojal et al. (2019) (34)<br><br>Mvundura et al. (2015) (35)<br><br>Mangale et al. (2022) (36)<br><br>UNICEF (2024) (37)	Syringe estimates were derived from UNICEF vaccine program estimates. Delivery overhead estimates were derived from the oral PrEP literature; provider time or overhead that is above and beyond resources needed to dispense oral PrEP are not included.
Product wastage	5% of the product goes to waste		Ojal et al. (2019) (34)	

Table S15 South Africa costing parameter calculations

**South Africa**				
**Cost parameter**	**Estimate (USD)**	**Year**	**Data source**	**Calculation detials, notes**
**HIV costs**				
Annual health care costs (among those not on ART)				
HIV-positive CD4 < 200	374.08	2021	Eaton 2014 (29)	Adjusted for inflation and GDP/capita ratio using this approach:<br>Adjust South Africa value in 2012 USD for inflation to be in 2021 USD ($374.08 = $167*2.24)<br>Cost of health care use, CD4 count <200 cells per µL, not in HIV care (per person-year) in South Africa= $167<br>USD Inflation Rate between time of costing (2012) and 2021: 2.24 =4.7/2.1
HIV-positive CD4 200 - 349	102.95	2021	Eaton 2014 (29)	Adjusted for inflation and GDP/capita ratio (see above)
HIV-positive CD4 > 350	29.10	2021	Eaton 2014 (29)	Adjusted for inflation and GDP/capita ratio (see above)
End of life care	358.10	2021	Eaton 2014 (29)	Adjust for inflation using World Bank CPI: the cost in 2021 USD for SA $160*4.7/2.1=$358.1 2021 USD.

Annual ART provision costs	189.56	2020	Long et al. (2010) (30)	1st line ART delivery cost: $ 119 (including personnel, building, equipment, etc) in 2018 USD → $124 in 2020 USD<br>Cost of 1st line ART: $124 USD (delivery cost in 2020 USD) + $43.20 (ART)*1.2(additional 20% supply chain)=$ 175.84; The delivery cost ratio between 2nd and 1st ART is 2.4;<br>Cost of 2nd line ART: $124*2.4 USD (delivery cost in 2020 USD) + $279.60*1.2=$633.12 per year; weighted average of 1st and 2nd lines ART cost assuming 3% on 2nd line ART: $175.84*0.97 + $633.12*0.03=$189.56
**Oral PrEP costs**				
Oral PrEP per person month, facility	15.20	2021	Jamieson et al. (2022) (38)<br><br>Jamieson et al. (2020) (38)	Ingredients-based approach, including rapid HIV testing, counselling, provision of condoms, syndromic screening with treatment referral, adherence counselling, traning, outreach, mobilization, monitoring and evaluation costs; Average cost of oral PrEP per person initiated:$76-78 across FSW, AGYW, and heterosexual men with a duration of 5 months use. This translates to a monthly cost of ~76/5=$15.20
Facility-based HIV-positive test	5.62	2018	Meyer-Rath et al. (2019) (39)	Including supply, furniture, and staff salaries
Facility-based HIV-negative test	3.62	2018	Meyer-Rath et al. (2019) (39)	Including supply, furniture, and staff salaries

Table S16 Zimbabwe costing parameter calculations

	**Kenya**			
**Cost parameter**	**Estimate**	**Year**	**Data source**	**Calculation details, notes**
**HIV costs**				
Annual health care costs (among those not on ART)				
HIV-positive CD4 < 200	93.98	2021	Eaton et al. (2014) (29)	Adjusted for inflation and GDP/capita ratio using this approach:<br>**Step 1:** Adjust South Africa value in 2012 USD for inflation to be in 2021 USD ($374.08 = $167*2.24)<br>Cost of health care use, CD4 count <200 cells per µL, not in HIV care (per person-year) in South Africa= $167<br>USD Inflation Rate between time of costing (2012) and 2021: 2.24 =4.7/2.1<br>**Step 2:** Adjust South Africa 2021 USD value by multiplying by the Zimbabwe GDP/cap ratio ($374.08*0.251)<br>South Africa 2021 GDP per capita in $USD = 7,055; ZA $USD = 1,774

				Kenya GDP/ ZA GDP ratio adjustment: (1,774/7,055)= 0.251
HIV-positive CD4 200 - 349	25.89	2021	Eaton et al. (2014) (29)	Adjusted for inflation and GDP/capita ratio (see steps above)
HIV-positive CD4 > 350	7.32	2021	Eaton et al. (2014) (29)	Adjusted for inflation and GDP/capita ratio (see steps above)
End of life care	89.83	2021	Eaton et al. (2014) (29)	Adjusted for inflation and GDP/capita ratio (see steps above)
Annual ART provision costs	176.01	2020	Long et al. (2010) (30)	1st line ART delivery cost, use ratio of Kenya:Zim GDPs (1774/2082=0.85): $131*0.85 =$111 in 2020 USD includes labs + staff encounters<br>Cost of 1st line ART: $111 USD (delivery cost in 2020 USD) + $43.20 (ART)*1.2(additional 20% suplly chain)=$ 162.84; The delivery cost ratio between 2nd and 1st ART is 2.4;<br>Cost of 2nd line ART: $111*2.4 USD (delivery cost in 2020 USD) + $279.60*1.2=$601.92; weighted average of 1st adn 2nd lines ART cost assuming 3% on 2nd line ART: $162.84*0.97 + $601.92*0.03=$176.01
**Oral PrEP costs**				
Oral PrEP per person month, facility	9.25	2019	Wanga et al. (2019) (31)	Translated from the cost from Kenya using ratio of Kenya:Zim GDPs (1774/2082=0.85)
Facility-based HIV-positive test	3.13	2017	Meisner et al. 2021 (32)	same with above
Facility-based HIV-negative test	2.24	2017	Meisner et al. 2021 (32)	same with above

Number of Oral PrEP initiations in Western Kenya, South Africa, and Zimbabwe

Table S17 Number of PrEP initiations in Western Kenya, South Africa, and Zimbabwe

	Western Kenya	South Africa	Zimbabwe
2016	655.2	722	288
2017	4376.4	3362	2677
2018	13208.4	8476	6131
2019	3370.8	45576	9302
2020	11543.6	106402	9501
2021	18007.2	205657	32019
2022	68220.4	422239	83580

Source: Global PrEP tracker, AVAC (40,41)

VOICE Risk Score

The VOICE risk score is an empirical HIV risk score derived from the VOICE trial to predict HIV acquisition risk in SSA with a higher score indicating a higher risk of HIV acquisition (42). The VOICE score can be calculated based on summing the point values that correspond to the following factors: age<25 years (2 points), unmarried or not living with a primary partner(2 points), partner does not provide financial or material support (1 point); primary partner has other partners (yes or don't know, 2 points); alcohol use in the past 3 months (1 point); having a curable sexually transmitted infection (1 point); herpes simplex virus type 2 (HSV-2) status (2 points). Due to several of these factors are not available in EMOD, we adapted the VOICE score calculation and only took the sum of the point values for the following factors: younger age (i.e., 15-24, 2 points), unmarried status (2 points), ≥ 1 male sexual partner who has other partners (2 points) and being identified as median sexual risk by EMOD (2 points). The modeled population in EMOD is stratified into three levels of sexual risk behavior groups: high-risk group representing sex workers and clients, medium risk group with short-term partnerships and increased propensity to form multiple concurrent partnerships, and low-risk group with fewer, lower-term partnerships (43). The median sexual risk category in EMOD was used as a proxy for other risk factors included in the original VOICE score calculation but not available in EMOD.

FSW and male client of FSW estimation

The sizes of the FSW and male client populations in each setting were estimated based on Ministry of Health surveillance, FSW enumeration studies, and DHS data. Details of this process have been previously published paper (43). Briefly, we utilize primary data on mean and standard deviation age of FSWs and employ a 0 to 5 year delay from sexual debut to onset of female sex work using a Weibull distribution; based on data, we assume that FSWs engage in sex work for a for a mean of 5.4 years (95% CI 2 - 9 years), parameterized using a uniform distribution. Triangulating the number of females engaging in sex work at any one time and the duration of sex work, we estimated the lifetime probability that a female would engage in FSW by country. Similarly, we used DHS data on number of men reporting ever having paid for sex in their lifetime and in the last 12 months to inform estimates of ever being a male client of FSWs by setting.

Table S18: Lifetime probability of becoming a female sex worker or male client of FSW in western Kenya by county

	Homa Bay	Kisii	Kisumu	Migori	Nyamira	Siaya
Prevalence of ever becoming FSW (among females)	0.0434	0.1302	0.14982	0.09936	0.05192	0.09406
Prevalence of ever becoming a male client of FSWs (among males)	0.063	0.2035	0.2341	0.1553	0.0811	0.147

Table S19: Lifetime probability of becoming a female sex worker or male client of FSW in South Africa

South Africa (national)	
Prevalence of ever becoming FSW (among females)	0.030
Prevalence of ever becoming a male client of FSWs (among males)	0.150

Table S20: Lifetime probability of becoming a female sex worker or male client of FSW in Zimbabwe

Lifetime probability of becoming a female sex worker or male client of FSW in South Africa

Zimbabwe (National)	
Prevalence of ever becoming FSW (among females)	0.125
Prevalence of ever becoming a male client of FSWs (among males)	0.195

Data sources:

Odek WO, Githuka GN, Avery L, Njoroge PK, Kasonde L, Gorgens M, Kimani J, Gelmon L, Gakii G, Isac S, Faran E, Musyoki H, Maina W, Blanchard JF, Moses S. Estimating the size of the female sex worker population in Kenya to inform HIV prevention programming. PLoS One. 2014 Mar 3;9(3):e89180.

Fearon E, Chabata ST, Magutshwa S, Ndori-Mharadze T, Musemburi S, Chidawanyika H, Masendeke A, Napierala S, Gonese E, Herman Roloff A, et al. Estimating the Population Size of Female Sex Workers in Zimbabwe: Comparison of Estimates Obtained Using Different Methods in Twenty Sites and Development of a National-Level Estimate. J Acquir Immune Defic Syndr. 2020 Sep 1;85(1):30-38.

Konstant TL, Rangasami J, Stacey MJ, Stewart ML, Nogoduka C. Estimating the number of sex workers in South Africa: rapid population size estimation. AIDS Behav. 2015 Feb;19 Suppl 1:S3-15. doi: 10.1007/s10461-014-0981-y. PMID: 25582921.

South African Health Monitoring Survey (SAHMS): A Biological and Behavioural Survey among Female Sex Workers, South Africa 2018: Final Report. Accessed from: https://auruminstitute.org/images/Docs/SAHMS_II_FSW_BBS_Full_Report.pdf on August 29 2024.

South Africa Demographic and Health Survey. Accessed from: https://dhsprogram.com/publications/publication-fr337-dhs-final-reports.cfm on August 29 2024.

Kenya Demographic and Health Survey. Accessed from: https://dhsprogram.com/pubs/pdf/PR143/PR143.pdf on August 29 2024.

Kenya Demographic and Health Survey. Accessed from: https://dhsprogram.com/methodology/survey/survey-display-556.cfm August 29 2024.

References

1. Pfau B, Ba AS, Cox SN, Wu L, Wittenauer R, Callen E, et al. User Preferences on Long-Acting Pre-Exposure Prophylaxis for HIV Prevention in Sub-Saharan Africa: A Scoping Review [Internet]. medRxiv; 2024 [cited 2024 Aug 14]. p. 2024.04.01.24305173. Available from: https://www.medrxiv.org/content/10.1101/2024.04.01.24305173v1

2. Bigogo G, Audi A, Aura B, Aol G, Breiman RF, Feikin DR. Health-seeking patterns among participants of population-based morbidity surveillance in rural western Kenya: implications for calculating disease rates. Int J Infect Dis. 2010;14(11):e967–73.

3. Abuya TO, Mutemi W, Karisa B, Ochola SA, Fegan G, Marsh V. Use of over-the-counter malaria medicines in children and adults in three districts in Kenya: implications for private medicine retailer interventions. Malar J. 2007 Dec;6(1):57.

4. Panzner U, Pak GD, Aaby P, Adu-Sarkodie Y, Ali M, Aseffa A, et al. Utilization of Healthcare in the Typhoid Fever Surveillance in Africa Program. Clin Infect Dis Off Publ Infect Dis Soc Am. 2016 Mar 15;62(Suppl 1):S56–68.

5. Abera Abaerei A, Ncayiyana J, Levin J. Health-care utilization and associated factors in Gauteng province, South Africa. Glob Health Action. 2017 Jan 1;10(1):1305765.

6. Lafort Y, Greener R, Roy A, Greener L, Ombidi W, Lessitala F, et al. Sexual and reproductive health services utilization by female sex workers is context-specific: results from a cross-sectional survey in India, Kenya, Mozambique and South Africa. Reprod Health. 2017 Jan 19;14(1):13.

7. Pande G, Bulage L, Kabwama S, Nsubuga F, Kyambadde P, Mugerwa S, et al. Preference and uptake of different community-based HIV testing service delivery models among female sex workers along Malaba-Kampala highway, Uganda, 2017. BMC Health Serv Res. 2019 Nov 5;19(1):799.

8. Richter M, Chersich MF, Vearey J, Sartorius B, Temmerman M, Luchters S. Migration Status, Work Conditions and Health Utilization of Female Sex Workers in Three South African Cities. J Immigr Minor Health. 2014;16(1):7–17.

9. Ngure K, Mugo NR, Bukusi EA, Kiptinness C, Oware K, Gakuo S, et al. Pills, injections, rings, or implants? PrEP formulation preferences of PrEP-experienced African women for HIV prevention. JAIDS J Acquir Immune Defic Syndr. 2021;88(4):e30–2.

10. Wara NJ, Mvududu R, Marwa MM, Gómez L, Mashele N, Orrell C, et al. Preferences and acceptability for long-acting PrEP agents among pregnant and postpartum women with experience using daily oral PrEP in South Africa and Kenya. J Int AIDS Soc. 2023 May;26(5):e26088.

11. Minnis AM, Atujuna M, Browne EN, Ndwayana S, Hartmann M, Sindelo S, et al. Preferences for long-acting pre-exposure prophylaxis (PrEP) for HIV prevention among South African youth: results of a discrete choice experiment. Afr J Reprod Gynaecol Endosc. 2020;23(6):e25528.

12. Montgomery ET, Atujuna M, Krogstad E, Hartmann M, Ndwayana S, O’Rourke S, et al. The invisible product: preferences for sustained-release, long-acting pre-exposure

prophylaxis to HIV among South African youth. JAIDS J Acquir Immune Defic Syndr. 2019;80(5):542–50.

13. Cheng CY, Quaife M, Eakle R, Cabrera Escobar MA, Vickerman P, Terris-Prestholt F. Determinants of heterosexual men’s demand for long-acting injectable pre-exposure prophylaxis (PrEP) for HIV in urban South Africa. BMC Public Health. 2019 Dec;19(1):996.

14. Tolley EE, Li S, Zangeneh SZ, Atujuna M, Musara P, Justman J, et al. Acceptability of a long-acting injectable HIV prevention product among US and African women: findings from a phase 2 clinical Trial (HPTN 076). Afr J Reprod Gynaecol Endosc [Internet]. 2019 [cited 2024 Aug 12];22(10). Available from: https://journals.lww.com/jrge/fulltext/2019/21100/Acceptability_of_a_long_acting_injectable_HIV.14.aspx

15. van der Straten A, Agot K, Ahmed K, Weinrib R, Browne EN, Manenzhe K, et al. The Tablets, Ring, Injections as Options (TRIO) study: what young African women chose and used for future HIV and pregnancy prevention. J Int AIDS Soc. 2018 Mar;21(3):e25094.

16. Jansen Van Vuuren CJ, Lewis L, Harkoo I, Dawood H, Mansoor LE. Experience with Contraceptive Dosage Forms and Interest in Novel PrEP Technologies in Women. AIDS Behav. 2023 Nov;27(11):3596–602.

17. Kidman R, Nachman S, Kohler HP. Interest in HIV pre-exposure prophylaxis (PrEP) among adolescents and their caregivers in Malawi. AIDS Care. 2020 May 13;32(sup2):23–31.

18. Mayanja Y, Kamacooko O, Lunkuse JF, Muturi-Kioi V, Buzibye A, Omali D, et al. Oral pre-exposure prophylaxis preference, uptake, adherence and continuation among adolescent girls and young women in Kampala, Uganda: a prospective cohort study. J Int AIDS Soc. 2022 May;25(5):e25909.

19. Chanda MM, Ortblad KF, Mwale M, Chongo S, Kanchele C, Kamungoma N, et al. HIV self-testing among female sex workers in Zambia: a cluster randomized controlled trial. PLoS Med. 2017;14(11):e1002442.

20. Luecke EH, Cheng H, Woeber K, Nakyanzi T, Mudekunye-Mahaka IC, van der Straten A, et al. Stated product formulation preferences for HIV pre-exposure prophylaxis among women in the VOICE-D (MTN-003D) study. Afr J Reprod Gynaecol Endosc [Internet]. 2016 [cited 2024 Aug 12];19(1). Available from: https://journals.lww.com/jrge/fulltext/2016/19010/Stated_product_formulation_preferences_for_HIV.58.aspx

21. Beckham SW, Mantsios A, Galai N, Likindikoki S, Mbwambo J, Davis W, et al. Acceptability of multiple modalities of pre-exposure prophylaxis (PrEP) among female sex workers in Tanzania: a mixed-methods study. BMJ Open. 2022;12(8):e058611.

22. Siedner MJ, Hettema A, Hughey A, Oldenburg CE, Kohler S, Bärnighausen K, et al. Preference for injectable over oral HIV pre-exposure prophylaxis in public-sector primary-care clinics in Swaziland. Aids. 2018;32(11):1541–2.

23. Were D. Preferences and Potential Demand for Multipurpose Prevention Technology (MPT) Implants and Other Longer-Acting PrEP Formulations in Kenya [Internet]. Jhpiego Unpubl; 2023 Feb [cited 2024 Jan 8]. Available from: www.unaids.org/sites/default/files/media_asset/prevention-2025-roadmap_en.pdf

24. Rousseau E. PrEPared to Choose: Choice counselling to empower providers and youth. 24th Int AIDS Conf. 2024;

25. Klein DJ. Relationship formation and flow control algorithms for generating age-structured networks in HIV modeling. In: 2012 IEEE 51st IEEE Conference on Decision and Control (CDC) [Internet]. IEEE; 2012 [cited 2024 Aug 14]. p. 1041–6. Available from: https://ieeexplore.ieee.org/abstract/document/6426573/?casa_token=QhJzbG6HaxIAAAAA:thKHcDxfjFJ9HMBSjtIGs3FjcPp5yOYR7sGszB6SObPKLiFDXH2ZBriQr5paafjIq9uV8XhBWQ

26. Institutes for Disease Modeling. Relationships and contact networks — HIV Model documentation [Internet]. [cited 2024 Aug 14]. Available from: https://docs.idmod.org/projects/emod-hiv/en/latest/stimodel-relationships.html

27. Bershteyn A, Klein D. STI and HIV Model Introduction [Internet]. Vancouver, Canada: Institute for Disease Modelling; 2015 [cited 2024 Aug 14]. Available from: https://institutefordiseasemodeling.github.io/EMOD/STI_and_HIV_Tutorials.pdf

28. Vos T, Lim SS, Abbafati C, Abbas KM, Abbasi M, Abbasifard M, et al. Global burden of 369 diseases and injuries in 204 countries and territories, 1990–2019: a systematic analysis for the Global Burden of Disease Study 2019. The Lancet. 2020 Oct 17;396(10258):1204–22.

29. Eaton JW, Menzies NA, Stover J, Cambiano V, Chindelevitch L, Cori A, et al. Health benefits, costs, and cost-effectiveness of earlier eligibility for adult antiretroviral therapy and expanded treatment coverage: a combined analysis of 12 mathematical models. Lancet Glob Health. 2014;2(1):e23–34.

30. Long L, Fox M, Sanne I, Rosen S. The high cost of second-line antiretroviral therapy for HIV/AIDS in South Africa. Aids. 2010;24(6):915–9.

31. Wanga V, Peebles K, Obiero A, Mogaka F, Omollo V, Odoyo JB, et al. Cost of pre-exposure prophylaxis delivery in family planning clinics to prevent HIV acquisition among adolescent girls and young women in Kisumu, Kenya. PLoS One. 2021;16(4):e0249625.

32. Meisner J, Roberts DA, Rodriguez P, Sharma M, Owiredu MN, Gomez B, et al. Optimizing HIV retesting during pregnancy and postpartum in four countries: a cost-effectiveness analysis. Afr J Reprod Gynaecol Endosc. 2021;24(4):e25686.

33. Galactionova K, Bertram M, Lauer J, Tediosi F. Costing RTS,S introduction in Burkina Faso, Ghana, Kenya, Senegal, Tanzania, and Uganda: A generalizable approach drawing on publicly available data. Vaccine. 2015 Nov 11;33(48):6710.

34. Ojal J, Griffiths U, Hammitt LL, Adetifa I, Akech D, Tabu C, et al. Sustaining pneumococcal vaccination after transitioning from Gavi support: a modelling and cost-effectiveness study in Kenya. Lancet Glob Health. 2019 May 1;7(5):e644–54.

35. Mvundura M, Lorenson K, Chweya A, Kigadye R, Bartholomew K, Makame M, et al. Estimating the costs of the vaccine supply chain and service delivery for selected districts in Kenya and Tanzania. Vaccine. 2015 May 28;33(23):2697–703.

36. Mangale D, Ortblad KF, Heitner J, Mogere P, Kiptinness C, Mugo N, et al. Comparing the cost of six-month PrEP dispensing with interim HIV self-testing to the standard-of-care three-month PrEP dispensing with clinic-based testing in Kenya. 24th Int AIDS Conf. 2022;

37. UNICEF Supply Division. Syringe and safety box bundles price data [Internet]. 2024 [cited 2024 Aug 14]. Available from: https://www.unicef.org/supply/documents/syringe-and-safety-box-bundles-price-data

38. Jamieson L, Gomez GB, Rebe K, Brown B, Subedar H, Jenkins S, et al. The impact of self-selection based on HIV risk on the cost-effectiveness of preexposure prophylaxis in South Africa. Aids. 2020;34(6):883–91.

39. Meyer-Rath G, van Rensburg C, Chiu C, Leuner R, Jamieson L, Cohen S. The per-patient costs of HIV services in South Africa: systematic review and application in the South African HIV investment case. PloS One. 2019;14(2):e0210497.

40. AVAC: Global Advocacy for HIV Prevention. A map-based tool to explore trends in PrEP use globally as countries introduce and scale up PrEP programs. [Internet]. 2022 [cited 2024 Aug 12]. Available from: https://data.prepwatch.org/

41. PrEPWatch [Internet]. [cited 2024 Jul 22]. 2024 Q1 Global PrEP Tracker. Available from: https://www.prepwatch.org/resources/global-prep-tracker/

42. Balkus JE, Brown E, Palanee T, Nair G, Gafoor Z, Zhang J, et al. An Empiric HIV Risk Scoring Tool to Predict HIV-1 Acquisition in African Women. J Acquir Immune Defic Syndr 1999. 2016 Jul 1;72(3):333–43.

43. Bershteyn A, Mutai KK, Akullian AN, Klein DJ, Jewell BL, Mwalili SM. The influence of mobility among high-risk populations on HIV transmission in Western Kenya. Infect Dis Model. 2018;3:97–106.

Supplemental Appendix Part II:

Additional Results

Table S1: Five-year budget impact analysis - main scenario*

Costs 2021 USD	ART	HIV testing	Illness	Lenacapavir	Oral PrEP	Total
**Kenya**						
**2026**	598 (-9,658-10,702)	-518 (-9,620-9,067)	-654 (-17,501-15,977)	109,145 (104,823-113,221)	-72 (-2,275-2,113)	108,500 (89,887-128,366)
**2027**	1,269 (-33,174-25,945)	739 (-19,543-23,762)	1,295 (-32,465-32,122)	511,609 (491,538-532,322)	-304 (-3,653-2,722)	514,609 (463,967-565,154)
**2028**	-7,317 (-57,020-34,239)	-381 (-28,510-26,795)	-3,915 (-45,995-48,932)	940,924 (905,327-976,905)	-564 (-4,377-2,370)	928,747 (853,022- 992,976)
**2029**	-23,076 (-76,911-27,004)	-4,064 (-45,952-30,595)	-10,361 (-63,341-39,034)	1,287,926 (1,239,114-1,340,491)	-750 (-3,639-2,384)	1,249,675 (1,173,326-1,348,527)
**2030**	-52,352 (-139,170-8,826)	-588 (-31,439-41,121)	-14,002 (-65,397-39,552)	1,352,654 (1,297,412-1,408,029)	-910 (-3,701-2,147)	1,284,802 (1,185,136-1,372,277)
**Zimbabwe**						
**2026**	4,422 (-86,509-97,020)	1,716 (-60,132-44,224)	-6,711 (-208,357-161,700)	449,411 (372,081-577,388)	2,023 (-62,663-78,781)	450,861 (231,596-644,784)
**2027**	3,899 (-308,356-280,334)	6,228 (-135,723-140,041)	-7,500 (-362,517-405,167)	2,091,999 (1,764,809-2,726,499)	-2,449 (-87,512-99,835)	2,092,177 (1,574,311-2,762,909)
**2028**	-33,819 (-448,030-380,004)	7,473 (-134,623-169,348)	-9,379 (-429,594-388,012)	3,826,941 (3,222,764-4,950,398)	-4,430 (-101,463-95,259)	3,786,785 (2,972,012-5,087,655)
**2029**	-81,827 (-608,861-433,635)	11,428 (-220,070-204,198)	-12,384 (-570,417-417,110)	5,255,650 (4,413,523-6,696,249)	-18,603 (-116,073-82,184)	5,154,264 (4,115,489-7,057,242)
**2030**	-152,235 (-806,483-428,085)	13,282 (-170,265-232,270)	-65,981 (-524,489-389,438)	5,539,841 (4,679,231-7,141,145)	-15,492 (-99,465-83,356)	5,319,415 (4,122,008- 7,370,707)
**South Africa**						
**2026**	-191,240 (-3,687,130-3,221,022)	-37,102 (-882,803-855,072)	136,001 (-4,041,431-5,001,473)	22,555,962 (19,966,912-25,607,930)	-45,189 (-681,159-487,307)	22,418,432 (17,244,933- 28,588,396)
**2027**	-867,145 (-9,134,705-7,569,881)	21,202 (-1,354,212-1,530,871)	-2,742 (-7,328,791-6,965,232)	71,379,936 (63,819,567-80,697,012)	-92,023 (-726,464-597,678)	70,439,229 (56,677,761- 85,601,275)
**2028**	-2,478,365 (-13,915,291-9,311,474)	-11,287 (-1,630,872-1,542,747)	153,445 (-8,558,129-8,828,681)	122,949,797 (110,085,834-139,028,641)	-171,695 (-769,228-681,205)	120,441,894 (102,823,061- 142,258,318)
**2029**	-5,345,799 (-19,354,290-11,086,700)	-8,836 (-1,543,391-1,576,058)	-549,516 (-12,035,809-9,826,002)	153,237,728 (136,887,260-171,468,172)	-184,454 (-812,938-375,945)	147,149,124 (126,802,110- 173,014,308)
**2030**	-9,058,861 (-24,774,120-9,020,046)	46,389 (-1,712,121-1,926,140)	-1,360,296 (-15,431,644-10,455,926)	157,403,032 (140,541,726-175,469,171)	-226,463 (-994,104-495,660)	146,803,800 (120,657,269-171,380,117)

*Budget impact for each category is calculated as the total cost in the Lenacapavir scenario minus the total cost in the standard of care scenario. Values in parathesis represent the 95% uncertainty interval across 100 simulations

Table S2: Five-year budget impact analysis - Higher lenacapavir coverage scenario*

	ART	HIV testing	Illness	Lenacapavir	Oral PrEP	Total
**Kenya**						
**2026**	172<br>(-9,663-9,414)	-276<br>(-11,986-9,717)	-1,640<br>(-17,950-15,960)	197,904<br>(189,374-205,684)	-73<br>(-2,550-2,129)	196,087<br>(176,033-212,656)
**2027**	-1,554<br>(-42,861-28,654)	-982<br>(-24,358-22,041)	-2,426<br>(-39,460-34,172)	929,592<br>(891,481-963,422)	-378<br>(-3,317-3,202)	924,252<br>(860,506- 995,097)
**2028**	-15,046<br>(-53,411-33,669)	-2,663<br>(-26,820-26,120)	-5,042<br>(-54,517-44,853)	1,716,222<br>(1,645,233-1,783,237)	-801<br>(-4,101-2,352)	1,692,670<br>(1,603,892-1,776,781)
**2029**	-46,814<br>(-99,645-9,614)	-4,644<br>(-45,410, 33,052)	-17,905<br>(-73,192-41,374)	2,358,613<br>(2,269,345-2,454,948)	-1,876<br>(-5,306-1,943)	2,287,374<br>(2,185,152-2,419,890)
**2030**	-101,109<br>(-157,669,-32,200)	-741<br>(-40,241-37,220)	-24,998<br>(-75,970-39,489)	2,480,094<br>(2,382,858-2,581,250)	-1,745<br>(-5,379-1,865)	2,351,502<br>(2,219,309-2,498,364)
**South Africa**						
**2026**	11,887<br>(-4,541,931-3,624,260)	20,903<br>(-1,032,983-843,447)	22,609<br>(-5,535,460-5,268,538)	36,590,444<br>(31,866,990-41,203,503)	-44,793<br>(-561,176-658,553)	36,601,049<br>(28,679,928-45,113,995)
**2027**	-876,205<br>(-9,028,238-7,873,421)	-84,211<br>(-1,184,761-1,383,958)	-337,786<br>(-7,428,363-6,303,533)	117,063,847<br>(104,244,419-132,074,544)	-271,039<br>(-940,996-439,586)	115,494,606<br>(97,929,171-133,194,088)
**2028**	-3,853,749<br>(-14,733,940-8,314,937)	49,630<br>(-1,450,532-1,262,742)	-418,045<br>(-10,551,637-10,125,512)	201,866,378<br>(180,817,995-227,079,565)	-414,574<br>(-1,057,122-292,494)	197,229,640<br>(170,752,264- 229,561,525)
**2029**	-8,908,284<br>(-20,532,338-5,515,834)	80,671<br>(-1,523,786-1,741,487)	-1,928,912<br>(-12,555,063-8,067,923)	253,669,880<br>(225,891,187-284,381,258)	-524,453<br>(-1,303,585-193,905)	242,388,901<br>(208,384,187- 280,324,980)
**2030**	-15,795,203<br>(-29,862,273-482,180)	205,673<br>(-1,759,965-1,883,472)	-2,999,234<br>(-16,867,474-10,486,753)	262,730,099<br>(232,963,491-295,973,542)	-563,109<br>(-1,160,032-135,606)	243,578,225<br>(209,629,502- 283,128,586)
**Zimbabwe**						
**2026**	10,112<br>(-74,096-92,977)	6,147<br>(-46,418-52,675)	-20,767<br>(-191,957-140,852)	784,594<br>(650,762-1,031,338)	-5,568<br>(-68,808-70,004)	774,517<br>(549,437-1,069,033)
**2027**	24,421<br>(-210,204-328,004)	1,028<br>(-147,777-170,901)	-497<br>(-325,594-337,184)	3,653,027<br>(3,060,826-4,818,256)	-7,473<br>(-108,179-95,394)	3,670,506<br>(2,907,375-4,868,673)
**2028**	-33,796<br>(-419,045-291,639)	-3,515<br>(-137,648-146,610)	-31,523<br>(-537,676-467,924)	6,727,725<br>(5,601,747-8,839,838)	-26,595<br>(-119,304-68,221)	6,632,296<br>(5,462,924-8,977,177)
**2029**	-129,656<br>(-565,982-238,431)	14,891<br>(-193,424-198,210)	-49,393<br>(-506,257-299,973)	9,239,225<br>(7,706,121-12,065,100)	-42,257<br>(-127,868-39,737)	9,032,810<br>(7,485,084-11,876,617)
**2030**	-260,280<br>(-801,502-220,918)	9,144<br>(-200,940-207,986)	-76,460<br>(-680,828-392,322)	9,764,000<br>(8,159,272-12,751,418)	-46,159<br>(-145,942-30,976)	9,390,244<br>(7,649,022-12,651,096)

**Budget impact for each category is calculated as the total cost in the Lenacapavir scenario minus the total cost in the standard of care scenario. Values in parathesis represent the 95% uncertainty interval across 100 simulations

Table S3 Five-year budget impact analysis: main scenario with lenacapavir price of $50 per dose*

Costs 2021 USD	ART	HIV testing	Illness	Lenacapavir	Oral PrEP	Total
**Kenya**						
**2026**	598 (-9,658-10,702)	-518 (-9,620-9,067)	-654 (-17,501-15,977)	249,528 (239,645-258,846)	-72 (-2,275-2,113)	248,883 (226,083-267,342)
**2027**	1,269 (-33,174-25,945)	739 (-19,543-23,762)	1,295 (-32,465-32,122)	1,169,641 (1,123,753-1,216,995)	-304 (-3,653-2,722)	1,172,641 (1,107,783-1,236,119)
**2028**	-7,317 (-57,020-34,239)	-381 (-28,510-26,795)	-3,915 (-45,995-48,932)	2,151,141 (2,069,758-2,233,400)	-564 (-4,377-2,370)	2,138,964 (2,040,201-2,244,920)
**2029**	-23,076 (-76,911-27,004)	-4,064 (-45,952-30,595)	-10,361 (-63,341-39,034)	2,944,456 (2,832,863-3,064,630)	-750 (-3,639-2,384)	2,906,205 (2,797,495-3,053,661)
**2030**	-52,352 (-139,170-8,826)	-588 (-31,439-41,121)	-14,002 (-65,397-39,552)	3,092,438 (2,966,143-3,219,036)	-910 (-3,701-2,147)	3,024,586 (2,872,707-3,173,869)
**Zimbabwe**						
**2026**	4,422 (-86,509-97,020)	1,716 (-60,132-44,224)	-6,711 (-208,357-161,700)	867,145 (717,937-1,114,078)	2,023 (-62,663-78,781)	868,595 (609,622-1,117,805)
**2027**	3,899 (-308,356-280,334)	6,228 (-135,723-140,041)	-7,500 (-362,517-405,167)	4,036,543 (3,405,226-5,260,821)	-2,449 (-87,512-99,835)	4,036,721 (3,319,621-5,211,219)
**2028**	-33,819 (-448,030-380,004)	7,473 (-134,623-169,348)	-9,379 (-429,594-388,012)	7,384,138 (6,218,372-9,551,867)	-4,430 (-101,463-95,259)	7,343,983 (6,042,631-9,631,483)
**2029**	-81,827 (-608,861-433,635)	11,428 (-220,070-204,198)	-12,384 (-570,417-417,110)	10,140,854 (8,515,957-12,920,512)	-18,603 (-116,073-82,184)	10,039,468 (8,192,464-13,236,149)
**2030**	-152,235 (-806,483-428,085)	13,282 (-170,265-232,270)	-65,981 (-524,489-389,438)	10,689,205 (9,028,646-13,778,945)	-15,492 (-99,465-83,356)	10,468,779 (8,668,543-14,009,206)
**South Africa**						
**2026**	-191,240 (-3,687,130-3,221,022)	-37,102 (-882,803-855,072)	136,001 (-4,041,431-5,001,473)	11,134,292 (9,856,260-12,640,834)	-45,189 (-681,159-487,307)	10,996,763 (6,419,118-16,756,069)
**2027**	-867,145 (-9,134,705-7,569,881)	21,202 (-1,354,212-1,530,871)	-2,742 (-7,328,791-6,965,232)	35,235,255 (31,503,232-39,834,440)	-92,023 (-726,464-597,678)	34,294,547 (21,674,490-46,364,101)
**2028**	-2,478,365 (-13,915,291-9,311,474)	-11,287 (-1,630,872-1,542,747)	153,445 (-8,558,129-8,828,681)	60,691,668 (54,341,634-68,628,663)	-171,695 (-769,228-681,205)	58,183,765 (44,823,009-74,537,093)
**2029**	-5,345,799 (-19,354,290-11,086,700)	-8,836 (-1,543,391-1,576,058)	-549,516 (-12,035,809-9,826,002)	75,642,690 (67,571,614-84,641,778)	-184,454 (-812,938-375,945)	69,554,085 (54,449,254-86,017,235)
**2030**	-9,058,861 (-24,774,120-9,020,046)	46,389 (-1,712,121-1,926,140)	-1,360,296 (-15,431,644-10,455,926)	77,698,807 (69,375,567-86,616,789)	-226,463 (-994,104-495,660)	67,099,576 (46,518,565-89,172,098)

**Budget impact for each category is calculated as the total cost in the Lenacapavir scenario minus the total cost in the standard of care scenario. Values in parathesis represent the 95% uncertainty interval across 100 simulations

Table S4: Health and budget impact and maximum price threshold for South Africa under varying scenarios of background oral PrEP*

	Main Scenario	Higher background oral PrEP scenario	58% background oral PrEP effectiveness	95% background oral PrEP effectiveness
**Lenacapavir coverage (%)**	1.6 (1.5-1.8)	1.6 (1.5-1.8)	1.6 (1.4-1.8)	1.6 (1.4-1.8)
**Infection averted (%)**	12.3 (5.4-19.5)	12.5 (5.4-18.5)	11.9 (4.2-21.3)	13.1 (5.5-21.0)
**Death averted (%)**	3.0 (-0.7-6.8)	3.1 (-1.1-7.1)	2.9 (3.0-7.6)	3.6 (2.4-8.1)
**Doses Required (5 years)**	3,942,509 (3,541,901-4,408,477)	3,959,450 (3,515,260-4,437,445)	3,869,458 (3,433,210-4,372,347)	3,889,630 (3,417,282-4,315,224)
**HIV infections averted per 1,000 doses**	28 (11-46)	28 (12-45)	28 (8-55)	30 (11-56)
**DALYs averted**	1,324,790 (-79,790-2,908,432)	1,312,541 (-226,047-2,868797)	1,312,026 (850,103-3,596,336)	1,651,701 (612,220-4,216,461)
**Max price per dose ($) ($500/DALY averted)**	106.28 (95.72-115.87)	108.21 (99.34-117.20)	106.13 (88.88-122.89)	101.07 (86.65-117.61)
**Max price per dose ($) ($200/DALY averted)**	74.87 (66.65- 82.33)	77.14 (69.98-84.14)	75.41 (65.30-86.96)	71.09 (59.62-80.55)
**5-year budget impact ($)**	507,252,479 (436,138,529-585,420,059)	522,058,105 (456,003,817-602,484,388)	519,881,715 (363,414,213-687,088,517)	487,113,336 (316,524,765-660,462,998)
**Modeled population size (2026)**	41,792,955 (41,519,588-42,030,162)	41,797,010 (41,525,744-42,048,639)	41,792,955 (41,519,588-42,030,162)	41,792,955 (41,519,588-42,030,162)

*Health impacts are compared to baseline scenario of daily oral PrEP only over. HIV infections averted and Lenacapavir coverage reported for age 15-49 over 10 of years of Lenacapavir implementation, deaths averted are calculated over the 35-year time horizon. Prices are in 2021 USD. Values in parentheses show 95% uncertainty intervals representing the 2.5th and 97.5th percentiles across 100 parameter sets.

Table S5: Health and budget impact and maximum price threshold varying lenacapavir effectiveness in men *

	Main scenario (95% effectiveness for both sexes)			80% lenacapavir effectiveness in men		
	**Western Kenya**	**Zimbabwe**	**South Africa**	**Western Kenya**	**Zimbabwe**	**South Africa**
**Lenacapavir coverage (%)**	2.9 (2.8-3.0)	4.0 (3.4-5.1)	1.6 (1.4-1.8)	2.9 (2.8-3.0)	4.0 (3.4-5.1)	1.6 (1.4-1.8)
**Infection averted (%)**	18.0 (11.0-22.9)	17.0 (3.3-28.2)	12.3 (5.4-19.5)	17.2 (11.8-22.5)	14.2 (1.30-27.8)	11.2 (2.8-20.1)
**Death averted (%)**	3.5 (0.1-6.6)	5.8 (-3.2-14.3)	3.0 (-0.7-6.8)	3.6 (0.1-6.8)	5.0 (-6.0-14.6)	2.6 (3.1-7.7)
**Doses Required (5 years)**	145,454 (139,757-151,137)	501,406 (421,531-645,223)	3,942,509 (3,541,901-4,408,477)	145,484 (139,814-151,069)	500,694 (420,877-643,617)	3,882,885 (3,434,260-4,316,709)
**HIV infections averted per 1,000 doses**	29 (17-40)	34 (6-74)	28 (11-46)	27 (17-36)	29 (3-69)	26 (6-52)
**DALYs averted**	45,315 (73,231-22,453)	282,649 (-12,843-833,338)	1,324,790 (-79,790-2,908,432)	43,493 (17,781-71,566)	247,710 (135,186-707,479)	1,218,712 (996,586-3,772,855)
**Max price per dose ($) ($500/DALY averted)**	16.58 (15.44-17.7)	21.15 (17.7-24.89)	106.28 (95.72-115.87)	15.29 (14.24-16.38)	17.59 (13.16-21.94)	99.2 (82.0-114.4)
**Max price per dose ($) ($200/DALY averted)**	11.32 (10.34,12.23)	11.68 (9.09-14.21)	74.87 (66.65-82.33)	10.21 (9.50-11.10)	9.09 (6.52-12.16)	69.24 (58.36-80.71)
**5-year budget impact ($)**	4,086,333 (3,863,089-4,297,703)	16,803,502 (13,951,046-22,639,690)	507,252,479 (436,138,529-585,420,059)	3,871,105 (3,655,496-4,080,375)	14,791,795 (12,216,409-19,509,836)	487,340,616 (324,605,098-633,326,000)
**Modeled population size (2026)**	4,299,746 (4,291,296-4,310,059)	10,683,464 (10,621,909-10,754,812)	41,792,955 (41,519,588-42,030,162)	4,299,746 (4,291,296-4,310,059)	10,683,464 (10,621,909-10,754,812)	41,792,955 (41,519,588-42,030,162)

* Health impacts are compared to baseline scenario of daily oral PrEP only over. HIV infections averted and Lenacapavir coverage reported for age 15-49 over 10 of years of Lenacapavir implementation, deaths averted are calculated over the 35-year time horizon. Prices are in 2021 USD. Values in parentheses show 95% uncertainty intervals representing the 2.5th and 97.5th percentiles across 100 parameter sets.

Figure S1: Sensitivity analyses: Expanding coverage to females only by VOICE score

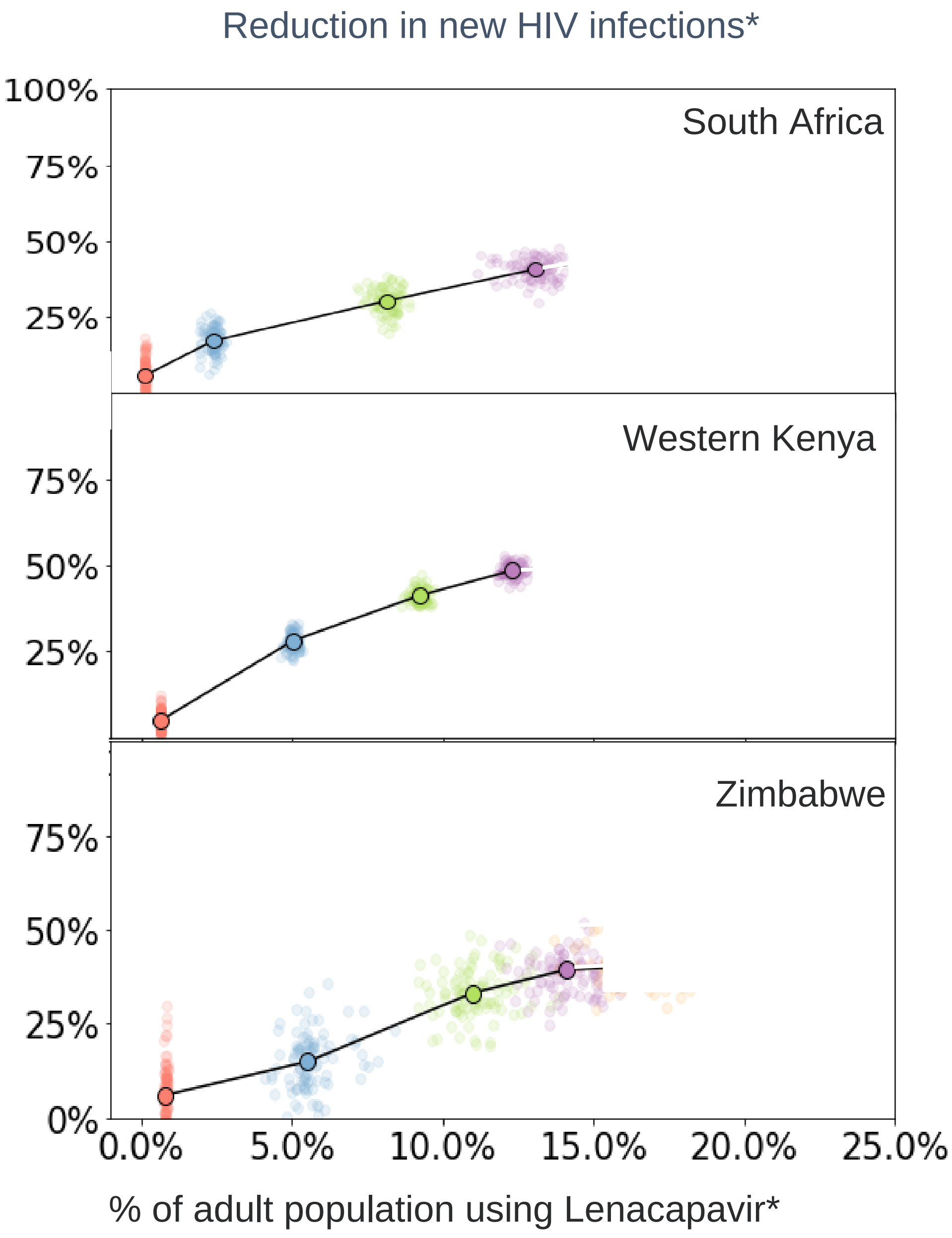

Per-dose price threshold

	Sex workers	VOICE score		
		≥ 5	≥ 3	≥ 1
South Africa	**$589** ($454, $726)	**$92** ($84, $100)	**$43** ($40, $45)	**$33** ($32, $35)
Western Kenya	**$23** ($18, 28)	**$14** ($13.2, $14.7)	**$8.9** ($8.5, $9.3)	**$6.4** ($6.1, $6.7)
Zimbabwe	**$39** ($28, $54)	**$4.9** ($2.6, $7.2)	**$6.0** ($4.7, $7.2)	**$4.3** ($3.2, $5.5)

* Reductions in HIV infections over 10 years of Lenacapavir implementation. Colored dots represent each of the 100 model simulations.
** Average % of adults ages 15-49 using Lenacapavir over implementation period.

Figure S2: Sensitivity analyses: Expanding lenacapavir coverage to females by VOICE score and males with >1 partner

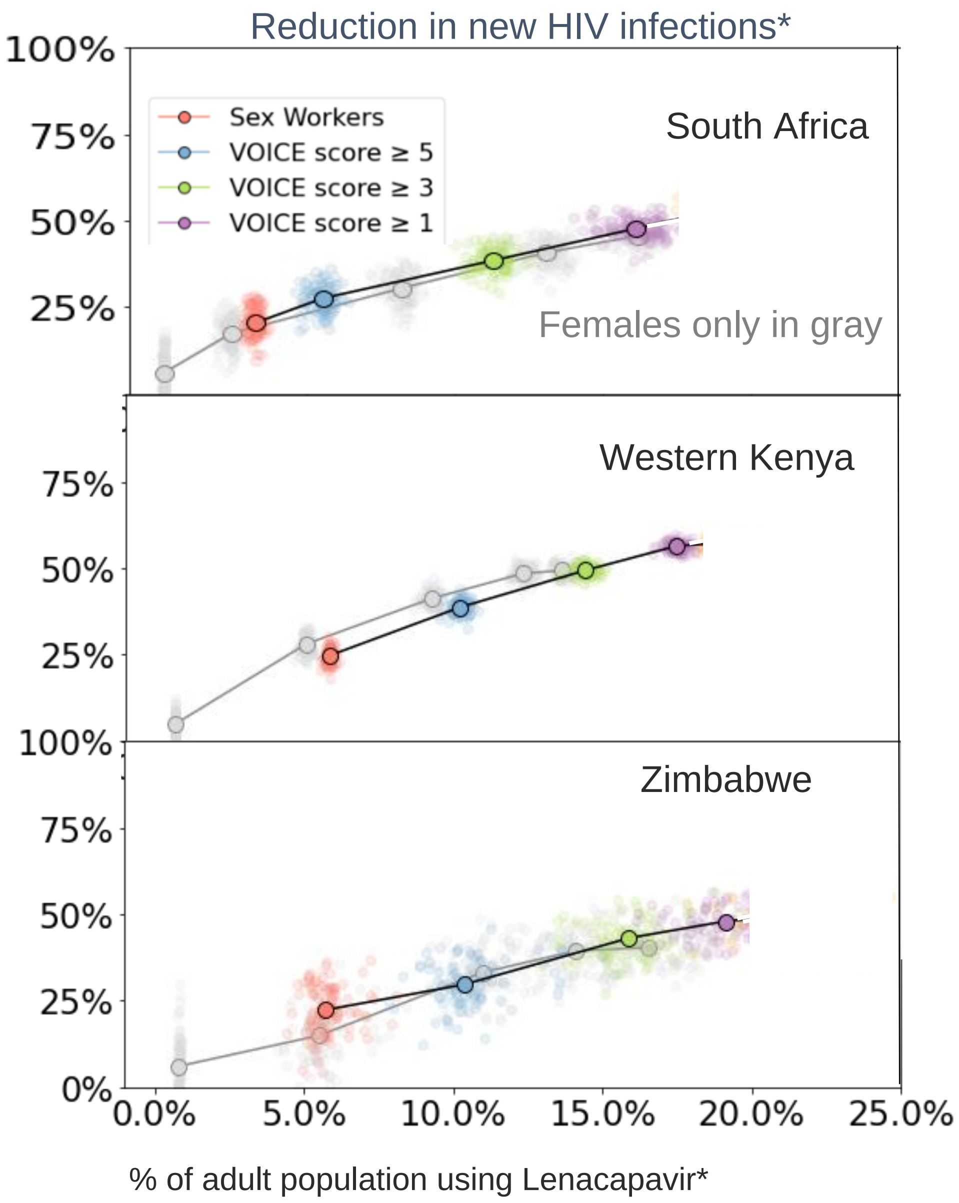

Per-dose price threshold¥

	Sex workers	VOICE score		
		≥ 5	≥ 3	≥ 1
South Africa	**$87** ($81, $92)	**$65** ($61, $69)	**$41** ($39, $42)	**$32** ($31, $33)
Western Kenya	**$10.2** ($9.6, $10.7)	**$7.5** ($7.2, $8.0)	**$5.4** ($5.2, $5.7)	**$4.2** ($4.0, $4.4)
Zimbabwe	**$13.3** ($11.2, $15.8)	**$7.2** ($5.9, $8.9)	**$5.3** ($4.5, $6.2)	**$4.0** ($3.3, $4.9)

* Reductions in HIV infections over 10 years of Lenacapavir implementation. Colored dots represent each of the 100 model simulations.
** Average % of adults ages 15-49 using Lenacapavir over implementation period.

¥ All scenarios include males with >1 partner

Table S6: Western Kenya: lenacapavir distribution to FSWs and males

	FSWs			FSWs and Male Clients			FSW and Males >1 partner		
	**Mean**	**CI Low**	**CI High**	**Mean**	**CI Low**	**CI High**	**Mean**	**CI Low**	**CI High**
**HIV Infections**	66,044	58,830	72,472	62,915	57,010	69,133	49,011	44,446	53,597
**HIV-related deaths**	66,382	60,430	71,172	65,545	60,503	70,403	61,399	57,172	66,127
**LEN doses (5 yrs)**	51,239	49,517	52,717	224,168	220,497	228,138	477,407	463,624	488,610
**LEN doses (10 yrs)**	116,189	113,627	118,445	511,014	504,350	518,005	1,090,121	1,061,123	1,118,122
**LEN PrEP coverage**	0.006	0.006	0.007	0.028	0.028	0.029	0.060	0.058	0.062
**Infections Averted (n)**	5,045	1,125	9,284	8,175	4,514	12,047	22,078	18,456	26,733
**Infections Averted (prop.)**	0.070	0.017	0.128	0.114	0.066	0.165	0.310	0.274	0.350
**Infections Averted per 1,000 doses**	43.4	10.0	80.0	16.0	8.7	23.7	20.3	16.8	24.9
**Deaths Averted (n)**	1,152	900	3,205	1,989	98	4,025	6,135	4,247	8,378
**Deaths Averted (prop.)**	0.017	0.013	0.050	0.029	0.001	0.058	0.091	0.065	0.118
**DALYs Averted**	20,974	5,693	49,030	35,608	11,368	61,086	103,533	78,963	127,316
**DALYs Averted per 1,000 doses**	181	49	415	70	22	120	95	71	119
**5 Year Budget Impact (USD)**	103,926,394	98,565,584	108,118,224	105,288,515	99,787,498	109,539,248	109,853,294	104,469,256	114,145,585
**Max price per dose**	23.00	18.00	28.00	6.50	5.30	7.50	10.20	9.60	10.70

[*] Health impacts are compared to baseline scenario of daily oral PrEP only over. HIV infections averted and Lenacapavir coverage reported for age 15-49 over 10 of years of Lenacapavir implementation, deaths averted are calculated over the 35-year time horizon. Prices are in 2021 USD. Values in parentheses show 95% uncertainty intervals representing the 2.5th and 97.5th percentiles across 100 parameter sets.

Table S7: Western Kenya: lenacapavir distribution to females VOICE≥5 and males

	Females only VOICE≥5			Females VOICE≥5 & Male Clients of FSWs			VOICE≥5 and Males >1 partner		
	**Mean**	**CI Low**	**CI High**	**Mean**	**CI Low**	**CI High**	**Mean**	**CI Low**	**CI High**
**HIV Infections**	45,998	42,509	51,014	43,851	40,620	47,904	36,581	33,495	39,982
**HIV-related deaths**	62,004	56,668	66,975	61,477	56,841	65,755	59,306	54,975	63,858
**5-year LEN doses**	407,169	390,109	424,861	580,424	563,675	597,811	833,731	807,015	863,810
**Total LEN doses**	925,062	884,674	961,994	1,321,054	1,283,631	1,359,420	1,901,173	1,837,570	1,965,977
**LEN PrEP coverage**	0.05	0.05	0.05	0.07	0.07	0.08	0.11	0.10	0.11
**Infections Averted (n)**	25,092	20,631	30,057	27,238	22,910	32,629	34,508	29,563	40,556
**Infections Averted (prop.)**	0.35	0.32	0.39	0.38	0.35	0.42	0.49	0.46	0.52
**Infections Averted per 1,000 doses**	27.1	22.5	32.9	20.6	17.6	24.9	18.2	15.6	21.3
**Deaths Averted (n)**	5,530	3,617	7,854	6,057	3,562	8,384	8,228	6,163	10,554
**Deaths Averted (prop.)**	0.08	0.06	0.11	0.09	0.05	0.12	0.12	0.09	0.15
**DALYs Averted**	102,248	75,313	132,013	112,287	83,902	140,705	147,353	120,521	176,788
**DALYs Averted per 1,000 doses**	111	83	143	85	62	105	78	65	92
**5 Year Budget Impact (USD)**	109,992,628	104,424,367	114,264,253	110,877,051	105,385,565	115,067,574	113,139,381	107,603,084	117,512,169
**Max price per dose**	14.00	13.20	14.70	9.40	8.90	9.90	7.50	7.20	8.00

[*] Health impacts are compared to baseline scenario of daily oral PrEP only over. HIV infections averted and Lenacapavir coverage reported for age 15-49 over 10 of years of Lenacapavir implementation, deaths averted are calculated over the 35-year time horizon. Prices are in 2021 USD. Values in parentheses show 95% uncertainty intervals representing the 2.5th and 97.5th percentiles across 100 parameter sets.

Table S8: Western Kenya: lenacapavir distribution to females VOICE≥3 and males

	Females only VOICE≥3			Females VOICE≥3 & Male Clients of FSWs			VOICE≥3 and Males >1 partner		
	**Mean**	**CI Low**	**CI High**	**Mean**	**CI Low**	**CI High**	**Mean**	**CI Low**	**CI High**
**HIV Infections**	32,407	28,803	35,670	30,907	28,383	34,213	25,998	23,683	28,333
**HIV-related deaths**	59,251	54,725	63,548	58,878	54,356	62,987	57,360	53,116	61,684
**LEN doses (5 yrs)**	802,176	771,248	833,671	975,460	945,046	1,003,873	1,228,989	1,189,660	1,269,295
**LEN doses (10 yrs)**	1,759,759	1,695,024	1,828,006	2,155,367	2,087,678	2,222,918	2,736,088	2,647,115	2,822,821
**LEN PrEP coverage**	0.097	0.093	0.101	0.119	0.115	0.122	0.151	0.146	0.155
**Infections Averted (n)**	38,682	34,037	44,517	40,182	34,961	46,538	45,092	39,942	51,800
**Infections Averted (prop.)**	0.544	0.515	0.574	0.565	0.534	0.591	0.634	0.609	0.660
**Infections Averted per 1,000 doses**	22.0	18.9	25.6	18.6	16.1	21.6	16.5	14.5	18.8
**Deaths Averted (n)**	8,283	6,351	10,514	8,656	6,426	10,842	10,175	7,842	12,810
**Deaths Averted (prop.)**	0.122	0.097	0.152	0.128	0.100	0.155	0.150	0.120	0.180
**DALYs Averted**	155,815	129,252	182,116	161,488	128,912	186,784	185,448	156,188	214,665
**DALYs Averted per 1,000 doses**	89	75	104	75	59	87	68	57	79
**5 Year Budget Impact (USD)**	113,737,660	108,129,304	117,962,809	114,039,696	108,424,167	118,150,567	115,951,299	110,360,448	120,211,433
**Max price per dose**	8.90	8.50	9.30	6.40	6.10	6.80	5.40	5.20	5.70

* Health impacts are compared to baseline scenario of daily oral PrEP only over. HIV infections averted and Lenacapavir coverage reported for age 15-49 over 10 of years of Lenacapavir implementation, deaths averted are calculated over the 35-year time horizon. Prices are in 2021 USD. Values in parentheses show 95% uncertainty intervals representing the 2.5th and 97.5th percentiles across 100 parameter sets.

Table S9: Western Kenya: lenacapavir distribution to females VOICE≥1 and males

	Females only VOICE≥1			Females VOICE≥1 & Male Clients of FSWs			VOICE≥1 and Males >1 partner		
	**Mean**	**CI Low**	**CI High**	**Mean**	**CI Low**	**CI High**	**Mean**	**CI Low**	**CI High**
**HIV Infections**	26,424	24,135	29,202	24,988	22,788	27,352	20,433	18,734	22,412
**HIV-related deaths**	58,099	53,681	62,285	57,755	53,004	61,995	56,421	51,779	60,850
**LEN doses (5 yrs)**	1,088,735	1,056,519	1,126,587	1,261,919	1,228,483	1,299,674	1,515,401	1,469,903	1,560,595
**LEN doses (10 yrs)**	2,354,608	2,286,728	2,434,464	2,750,316	2,678,950	2,829,133	3,330,383	3,233,422	3,426,022
**LEN PrEP coverage**	0.13	0.13	0.13	0.15	0.15	0.16	0.18	0.18	0.19
**Infections Averted (n)**	44,666	39,053	51,456	46,101	41,037	52,736	50,656	44,845	57,681
**Infections Averted (prop.)**	0.63	0.60	0.66	0.65	0.63	0.67	0.71	0.69	0.73
**Infections Averted per 1,000 doses**	19	17	22	17	15	19	15	13	17
**Deaths Averted (n)**	9,435	7,110	11,808	9,779	7,574	12,776	11,113	9,186	13,782
**Deaths Averted (prop.)**	0.139	0.111	0.167	0.144	0.117	0.180	0.164	0.140	0.194
**DALYs Averted**	178,260	147,249	211,780	184,339	154,155	219,058	205,974	176,917	238,319
**DALYs Averted per 1,000 doses**	76	63	89	67	56	79	62	53	72
**5 Year Budget Impact**	115,430,512	109,722,442	119,621,788	115,948,751	110,400,110	120,256,594	117,553,634	112,023,247	121,900,073
**Max price per dose**	6.40	6.10	6.70	5.00	4.70	5.20	4.20	4.00	4.40

[*] Health impacts are compared to baseline scenario of daily oral PrEP only over. HIV infections averted and Lenacapavir coverage reported for age 15-49 over 10 of years of Lenacapavir implementation, deaths averted are calculated over the 35-year time horizon. Prices are in 2021 USD. Values in parentheses show 95% uncertainty intervals representing the 2.5th and 97.5th percentiles across 100 parameter sets.

Table S10: Zimbabwe: lenacapavir distribution to female sex workers (FSWs) and males

	FSWs			FSWs and Male Clients			FSW and Males >1 partner		
	**Mean**	**CI Low**	**CI High**	**Mean**	**CI Low**	**CI High**	**Mean**	**CI Low**	**CI High**
**HIV Infections**	262,357	192,000	355,126	254,991	186,070	333,603	217,454	157,349	281,978
**HIV-related deaths**	417,600	318,258	530,666	414,596	318,519	527,299	395,882	304,211	497,142
**LEN doses (5 yrs)**	153,780	141,632	166,463	467,175	441,311	494,855	1,056,361	905,258	1,320,277
**LEN doses (10 yrs)**	308,747	287,561	333,971	967,767	916,451	1,018,532	2,171,033	1,882,575	2,699,276
**LEN PrEP coverage**	0.007	0.006	0.007	0.021	0.020	0.023	0.048	0.042	0.059
**Infections Averted (n)**	17,660	20,775	51,764	25,026	10,989	68,697	62,563	22,260	112,284
**Infections Averted (prop.)**	0.057	0.092	0.172	0.082	0.050	0.203	0.217	0.101	0.338
**Infections Averted per 1,000 doses**	57	66	166	26	11	72	29	11	54
**Deaths Averted (n)**	10,579	18,232	47,968	13,583	17,954	46,882	32,297	2,023	71,764
**Deaths Averted (prop.)**	0.023	0.045	0.097	0.029	0.041	0.092	0.074	0.006	0.147
**DALYs Averted**	102,673	168,488	385,883	140,785	154,505	468,529	344,622	28,636	700,681
**DALYs Averted per 1,000 doses**	332	523	1,324	147	161	484	161	13	336
**5 Year Budget Impact**	265,493,674	231,704,027	305,201,413	264,014,121	231,006,775	302,750,986	274,207,153	239,883,402	314,675,166
**Max price per dose**	39.00	28.00	54.00	13.00	8.00	17.00	13.30	11.20	15.80

* Health impacts are compared to baseline scenario of daily oral PrEP only over. HIV infections averted and Lenacapavir coverage reported for age 15-49 over 10 of years of Lenacapavir implementation, deaths averted are calculated over the 35-year time horizon. Prices are in 2021 USD. Values in parentheses show 95% uncertainty intervals representing the 2.5th and 97.5th percentiles across 100 parameter sets.

Table S11: Zimbabwe: lenacapavir distribution to females VOICE≥5 and males

	Females only VOICE≥5			Females VOICE≥5 & Male Clients of FSWs			VOICE≥5 and Males >1 partner		
	**Mean**	**CI Low**	**CI High**	**Mean**	**CI Low**	**CI High**	**Mean**	**CI Low**	**CI High**
**HIV Infections**	241,012	181,591	312,530	228,744	176,010	297,428	197,439	144,274	248,734
**HIV-related deaths**	412,439	312,932	525,592	403,634	311,063	508,611	387,274	294,833	486,801
**LEN doses (5 yrs)**	1,032,353	819,176	1,346,094	1,346,429	1,135,391	1,666,492	1,936,905	1,630,874	2,496,153
**LEN doses (10 yrs)**	2,110,752	1,686,810	2,753,712	2,769,453	2,318,185	3,414,117	3,975,712	3,364,221	5,111,979
**LEN PrEP coverage**	0.047	0.038	0.061	0.061	0.051	0.075	0.088	0.074	0.113
**Infections Averted (n)**	39,005	354	85,738	51,273	9,229	98,448	82,578	38,845	136,333
**Infections Averted (prop.)**	0.130	0.002	0.254	0.174	0.051	0.301	0.288	0.186	0.405
**Infections Averted per 1,000 doses**	18.41	0.16	43.45	18.50	3.57	37.70	20.87	10.30	35.48
**Deaths Averted (n)**	15,740	18,514	50,286	24,546	16,451	63,291	40,905	3,540	94,950
**Deaths Averted (prop.)**	0.034	0.051	0.110	0.054	0.040	0.142	0.092	0.008	0.178
**DALYs Averted**	195,866	164,748	491,487	268,334	92,308	600,970	451,940	135,063	910,543
**DALYs Averted per 1,000 doses**	92	75	241	96	38	213	114	34	242
**5 Year Budget Impact**	268,810,195	234,274,604	309,398,849	269,350,148	235,284,302	309,675,209	278,111,435	243,026,859	321,762,670
**Max price per dose**	4.90	2.60	7.20	5.60	4.00	7.40	7.20	5.90	8.90

[*] Health impacts are compared to baseline scenario of daily oral PrEP only over. HIV infections averted and Lenacapavir coverage reported for age 15-49 over 10 of years of Lenacapavir implementation, deaths averted are calculated over the 35-year time horizon. Prices are in 2021 USD. Values in parentheses show 95% uncertainty intervals representing the 2.5th and 97.5th percentiles across 100 parameter sets.

Table S12: Zimbabwe: lenacapavir distribution to females VOICE≥3 and males

	Females only VOICE≥3			Females VOICE≥3 & Male Clients of FSWs			VOICE≥3 and Males >1 partner		
	**Mean**	**CI Low**	**CI High**	**Mean**	**CI Low**	**CI High**	**Mean**	**CI Low**	**CI High**
**HIV Infections**	192,955	139,641	257,589	186,405	140,772	240,798	165,253	117,137	219,305
**HIV-related deaths**	391,670	301,665	499,886	386,987	295,225	492,585	375,152	284,038	482,836
**LEN doses (5 yrs)**	2,014,993	1,737,804	2,316,826	2,330,340	2,034,209	2,639,880	2,919,646	2,455,323	3,450,648
**LEN doses (10 yrs)**	4,138,584	3,594,592	4,755,734	4,804,696	4,208,939	5,453,506	6,008,055	5,143,986	7,077,561
**LEN PrEP coverage**	0.092	0.079	0.106	0.106	0.094	0.121	0.133	0.113	0.157
**Infections Averted (n)**	87,062	42,968	146,755	93,612	53,041	148,263	114,764	67,839	173,897
**Infections Averted (prop.)**	0.31	0.19	0.40	0.33	0.24	0.42	0.40	0.30	0.51
**Infections Averted per 1,000 doses**	21.1	10.0	35.5	19.6	10.9	30.9	19.2	11.6	29.0
**Deaths Averted (n)**	36,509	5,377	82,574	41,192	11,269	78,879	53,027	17,392	93,377
**Deaths Averted (prop.)**	0.08	0.01	0.17	0.09	0.03	0.16	0.12	0.04	0.19
**DALYs Averted**	426,735	110,790	839,665	471,337	220,032	864,103	595,498	291,298	1,042,021
**DALYs Averted per 1,000 doses**	104	26	206	99	46	179	99	46	162
**5 Year Budget Impact**	279,775,320	245,652,932	320,010,897	278,545,428	244,679,099	318,107,549	284,588,009	249,824,317	326,438,480
**Max price per dose**	6.00	4.70	7.20	5.30	4.30	6.30	5.30	4.50	6.20

[*] Health impacts are compared to baseline scenario of daily oral PrEP only over. HIV infections averted and Lenacapavir coverage reported for age 15-49 over 10 of years of Lenacapavir implementation, deaths averted are calculated over the 35-year time horizon. Prices are in 2021 USD. Values in parentheses show 95% uncertainty intervals representing the 2.5th and 97.5th percentiles across 100 parameter sets.

Table S13: Zimbabwe: lenacapavir distribution to females VOICE≥1 and males

	Females only VOICE≥1			Females VOICE≥1 & Male Clients of FSWs			VOICE≥1 and Males >1 partner		
	**Mean**	**CI Low**	**CI High**	**Mean**	**CI Low**	**CI High**	**Mean**	**CI Low**	**CI High**
**HIV Infections**	180,509	137,823	242,395	171,387	127,090	229,617	154,006	111,462	204,547
**HIV-related deaths**	386,757	303,656	492,854	383,617	298,730	490,751	369,923	285,743	467,507
**LEN doses (5 yrs)**	2,620,621	2,292,096	2,922,073	2,937,429	2,626,878	3,240,703	3,528,274	3,047,249	3,969,289
**LEN doses (10 yrs)**	5,329,747	4,682,873	5,948,679	5,990,663	5,358,948	6,638,811	7,199,858	6,257,267	8,102,494
**LEN PrEP coverage**	0.12	0.10	0.13	0.13	0.12	0.15	0.16	0.14	0.18
**Infections Averted (n)**	99,508	56,547	155,833	108,630	61,758	159,341	126,011	81,182	176,241
**Infections Averted (prop.)**	0.35	0.26	0.44	0.38	0.28	0.48	0.45	0.37	0.53
**Infections Averted per 1,000 doses**	18.763	10.967	30.076	18.232	9.886	28.755	17.590	10.669	27.396
**Deaths Averted (n)**	41,422	6,435	82,421	44,562	12,759	83,036	58,256	24,537	101,067
**Deaths Averted (prop.)**	0.09	0.02	0.18	0.10	0.04	0.17	0.13	0.06	0.21
**DALYs Averted**	486,428	177,157	822,435	526,992	239,923	900,238	648,602	344,857	1,114,948
**DALYs Averted per 1,000 doses**	92	33	156	88	38	154	91	46	153
**5 Year Budget Impact**	282,599,968	248,604,820	322,059,223	281,144,111	246,394,335	319,726,238	286,885,555	251,714,351	327,393,991
**Max price per dose**	4.30	3.20	5.50	3.90	3.00	4.70	4.00	3.30	4.90

* Health impacts are compared to baseline scenario of daily oral PrEP only over. HIV infections averted and Lenacapavir coverage reported for age 15-49 over 10 of years of Lenacapavir implementation, deaths averted are calculated over the 35-year time horizon. Prices are in 2021 USD. Values in parentheses show 95% uncertainty intervals representing the 2.5th and 97.5th percentiles across 100 parameter sets.

Table S14: South Africa: lenacapavir distribution to female sex workers (FSWs) and males

	FSWs			FSWs and Male Clients			FSW and Males >1 partner		
	**Mean**	**CI Low**	**CI High**	**Mean**	**CI Low**	**CI High**	**Mean**	**CI Low**	**CI High**
**HIV Infections**	2,289,509	1,983,132	2,556,430	2,118,620	1,880,365	2,392,208	1,969,688	1,721,410	2,269,778
**HIV-related deaths**	4,248,287	3,726,125	4,726,742	4,178,874	3,649,992	4,692,426	4,122,014	3,614,253	4,616,165
**LEN doses (5 yrs)**	479,916	402,361	565,211	5,872,186	5,513,199	6,338,869	11,163,581	10,157,411	12,127,878
**LEN doses (10 yrs)**	933,625	790,955	1,072,783	11,213,480	10,532,624	11,975,192	21,212,862	19,384,506	22,975,877
**LEN PrEP coverage**	0.001	0.001	0.001	0.014	0.013	0.015	0.026	0.024	0.028
**Infections Averted (n)**	136,637	25,386	339,713	307,526	142,327	479,928	456,458	283,293	615,728
**Infections Averted (prop.)**	0.06	0.01	0.14	0.13	0.06	0.19	0.19	0.13	0.25
**Infections Averted per 1,000 doses**	146.224	26.820	346.930	27.463	12.705	43.751	21.661	12.572	30.450
**Deaths Averted (n)**	57,214	104,572	214,430	126,627	16,073	276,126	183,487	53,787	307,764
**Deaths Averted (prop.)**	0.013	0.025	0.049	0.029	0.004	0.065	0.043	0.013	0.071
**DALYs Averted**	548,139	819,950	2,001,765	1,282,165	21,334	2,469,982	1,939,766	510,789	3,163,054
**DALYs Averted per 1,000 doses**	591.598	902.939	2,089.348	114.119	1.921	218.595	91.840	25.634	149.937
**5 Year Budget Impact**	7,344,912,783	6,648,777,695	8,024,850,880	7,680,064,982	7,026,485,389	8,362,511,693	7,976,659,644	7,337,853,884	8,587,317,673
**Max price per dose**	589.00	454.00	726.00	112.00	102.00	123.00	87.00	81.00	92.00

* Health impacts are compared to baseline scenario of daily oral PrEP only over. HIV infections averted and Lenacapavir coverage reported for age 15-49 over 10 of years of Lenacapavir implementation, deaths averted are calculated over the 35-year time horizon. Prices are in 2021 USD. Values in parentheses show 95% uncertainty intervals representing the 2.5th and 97.5th percentiles across 100 parameter sets.

Table S15: South Africa: lenacapavir distribution to females VOICE≥5 and males

	Females only VOICE≥5			Females VOICE≥5 & Male Clients of FSWs			VOICE≥5 and Males >1 partner		
	**Mean**	**CI Low**	**CI High**	**Mean**	**CI Low**	**CI High**	**Mean**	**CI Low**	**CI High**
HIV Infections	2,047,511	1,835,757	2,284,787	1,934,154	1,715,490	2,152,971	1,826,668	1,603,269	2,094,663
HIV-related deaths	4,159,870	3,680,208	4,645,242	4,103,070	3,620,870	4,627,896	4,050,787	3,533,069	4,533,181
LEN doses (5 yrs)	8,314,922	6,909,900	9,320,727	13,742,716	12,130,735	15,036,659	19,029,719	16,775,709	20,819,387
LEN doses (10 yrs)	15,682,232	13,020,805	17,477,003	26,013,834	23,317,847	28,207,302	36,023,122	31,812,452	39,054,018
LEN PrEP coverage	0.019	0.016	0.022	0.032	0.029	0.035	0.044	0.039	0.048
Infections Averted (n)	378,635	224,297	548,156	491,992	326,064	699,832	599,478	446,637	779,684
Infections Averted (prop.)	0.16	0.10	0.22	0.20	0.14	0.26	0.25	0.20	0.31
Infections Averted per 1,000 doses	24.4	13.9	37.6	19.0	12.4	27.8	16.8	12.5	23.7
Deaths Averted (n)	145,631	4,926	271,762	202,431	60,344	353,439	254,714	109,699	399,816
Deaths Averted (prop.)	0.034	0.001	0.063	0.047	0.015	0.083	0.059	0.029	0.095
DALYs Averted	1,483,412	218,083	2,819,972	2,055,762	795,644	3,266,038	2,522,383	1,322,796	3,911,081
DALYs Averted per 1,000 doses	96	14	178	80	31	131	70	34	111
5 Year Budget Impact	7,788,872,795	7,132,190,126	8,433,304,193	8,036,996,047	7,375,510,581	8,682,138,215	8,231,620,084	7,566,276,893	8,895,846,456
Max price per dose	92.00	84.00	100.00	76.00	71.00	81.00	65.00	61.00	69.00

[*] Health impacts are compared to baseline scenario of daily oral PrEP only over. HIV infections averted and Lenacapavir coverage reported for age 15-49 over 10 of years of Lenacapavir implementation, deaths averted are calculated over the 35-year time horizon. Prices are in 2021 USD. Values in parentheses show 95% uncertainty intervals representing the 2.5th and 97.5th percentiles across 100 parameter sets.

Table S16: South Africa: lenacapavir distribution to females VOICE≥3 and males

	Females only VOICE≥3			Females VOICE≥3 & Male Clients of FSWs			VOICE≥3 and Males >1 partner		
	**Mean**	**CI Low**	**CI High**	**Mean**	**CI Low**	**CI High**	**Mean**	**CI Low**	**CI High**
**HIV Infections**	1,765,726	1,532,175	1,991,446	1,678,128	1,479,334	1,875,318	1,584,379	1,401,725	1,797,341
**HIV-related deaths**	4,055,255	3,601,778	4,476,272	4,016,094	3,532,696	4,496,736	3,956,652	3,433,242	4,398,056
**LEN doses (5 yrs)**	28,802,245	26,409,891	30,559,556	34,214,140	31,565,877	36,198,071	39,531,709	36,545,543	41,789,742
**LEN doses (10 yrs)**	53,545,933	49,328,410	56,819,452	63,867,163	59,612,147	67,705,384	73,967,440	68,541,172	78,260,816
**LEN PrEP coverage**	0.066	0.061	0.070	0.079	0.073	0.083	0.091	0.085	0.097
**Infections Averted (n)**	660,420	452,387	876,139	748,018	574,292	939,616	841,766	652,972	1,066,846
**Infections Averted (prop.)**	0.27	0.20	0.34	0.31	0.26	0.36	0.35	0.30	0.39
**Infections Averted per 1,000 doses**	12.4	8.5	16.6	11.7	8.8	15.3	11.4	8.9	14.6
**Deaths Averted (n)**	250,247	96,094	399,955	289,407	148,923	422,403	348,850	200,819	493,524
**Deaths Averted (prop.)**	0.058	0.023	0.088	0.067	0.032	0.098	0.081	0.050	0.115
**DALYs Averted**	2,578,975	1,297,933	3,777,709	2,968,781	1,772,623	4,213,474	3,554,900	2,307,312	4,913,849
**DALYs Averted per 1,000 doses**	48	24	73	47	27	67	48	32	65
**5 Year Budget Impact**	8,319,881,476	7,690,791,407	8,979,908,912	8,469,077,577	7,847,043,197	9,145,051,645	8,687,490,286	8,056,025,146	9,348,067,578
**Max price per dose**	43.00	40.00	45.00	41.00	39.00	43.00	41.00	39.00	42.00

[*] Health impacts are compared to baseline scenario of daily oral PrEP only over. HIV infections averted and Lenacapavir coverage reported for age 15-49 over 10 of years of Lenacapavir implementation, deaths averted are calculated over the 35-year time horizon. Prices are in 2021 USD. Values in parentheses show 95% uncertainty intervals representing the 2.5th and 97.5th percentiles across 100 parameter sets.

Table S17: South Africa: lenacapavir distribution to females VOICE≥1 and males

	Females only VOICE≥1			Females VOICE≥1 & Male Clients of FSWs			VOICE≥1 and Males >1 partner		
	**Mean**	**CI Low**	**CI High**	**Mean**	**CI Low**	**CI High**	**Mean**	**CI Low**	**CI High**
**HIV Infections**	1,553,773	1,384,915	1,739,361	1,459,923	1,290,115	1,625,904	1,393,791	1,235,376	1,553,787
**HIV-related deaths**	3,974,636	3,484,547	4,453,847	3,932,357	3,432,637	4,430,777	3,902,504	3,401,788	4,443,105
**LEN doses (5 yrs)**	46,014,102	41,543,544	48,925,754	51,417,577	46,922,192	54,458,722	56,772,999	51,740,681	60,220,576
**LEN doses (10 yrs)**	85,593,939	77,400,981	91,040,671	95,923,382	87,352,828	101,451,040	106,048,727	97,231,010	112,214,167
**LEN PrEP coverage**	0.105	0.095	0.112	0.118	0.108	0.126	0.130	0.119	0.138
**Infections Averted (n)**	872,373	682,130	1,080,884	966,223	763,067	1,162,424	1,032,355	834,267	1,232,363
**Infections Averted (prop.)**	0.358	0.302	0.412	0.398	0.351	0.435	0.425	0.383	0.469
**Infections Averted per 1,000 doses**	10.2	7.7	13.1	10.1	7.9	12.6	9.8	7.8	12.1
**Deaths Averted (n)**	330,865	194,439	482,674	373,145	243,365	545,430	402,997	259,268	553,254
**Deaths Averted (prop.)**	0.077	0.046	0.107	0.087	0.057	0.119	0.094	0.061	0.126
**DALYs Averted**	3,424,076	2,008,845	4,624,508	3,867,288	2,668,754	5,400,402	4,137,154	2,714,364	5,423,547
**DALYs Averted per 1,000 doses**	40	24	56	40	27	56	39	24	51
**5 Year Budget Impact**	8,659,482,597	8,057,852,317	9,264,500,355	8,822,827,206	8,199,717,334	9,432,025,013	8,954,499,222	8,342,195,004	9,548,457,366
**Max price per dose**	33.00	32.00	35.00	33.00	32.00	34.00	32.00	31.00	33.00

[*] Health impacts are compared to baseline scenario of daily oral PrEP only over. HIV infections averted and Lenacapavir coverage reported for age 15-49 over 10 of years of Lenacapavir implementation, deaths averted are calculated over the 35-year time horizon. Prices are in 2021 USD. Values in parentheses show 95% uncertainty intervals representing the 2.5th and 97.5th percentiles across 100 parameter sets.

Table S18: Summary of HIV incidence reduction and maximum price per dose for expanding lenacapavir distribution scenarios

	South Africa		Western Kenya		Zimbabwe	
	Price threshold	HIV incidence reduction (%)	Price threshold	HIV incidence reduction (%)	Price threshold	HIV incidence reduction (%)
FSWs						
FSWs only	$589.0 (454.0 – 726.0)	6.0% (1.0 – 14.0)	$23.0 (18.0 – 28.0)	7.0% (1.7 – 12.8)	$39.0 (28.0 – 54.0)	5.7 (9.2 – 17.2)
FSWs and male clients of FSWs	$112.0 (102.0 – 123.0)	13.0% (6.0 – 19.0)	$6.5 (5.3 – 7.5)	11.4% (6.6 – 16.5)	$13.0 (8.0 – 17.0)	8.2 (5.0 – 20.3)
FSWs and males >1 partner	$87.0 (81.0 – 92.0)	19.0% (13.0 – 25.0)	$10.2 (9.6 – 10.7)	31.0% (27.4 – 35.0)	$13.3 (13.0 – 15.8)	21.7 (10.1 – 33.8)
VOICE Scores						
Females VOICE > 5 only	$92.0 (84.0 – 100.0)	16.0% (10.0 – 22.0)	$14.0 (7.4 –13.2)	35.0% (32.0 – 39.0)	$4.9 (2.6 – 7.2)	13.0 (0.2 – 25.4)
Females VOICE > 5, and male clients of FSWs	$76.0 (71.0 – 81.0)	20.0% (14.0 – 26.0)	$9.4 (8.9 – 9.9)	38.0% (35.0 – 42.0)	$5.6 (4.0 – 7.4)	17.4 (5.1 – 30.1)
Females VOICE > 5, and males >1 partner	$65.0 (61.0 – 69.0)	25.0% (20.0 – 31.0)	$7.5 (7.2 – 8.0)	49.0% (49.0 – 52.0)	$7.2 (5.9 – 8.9)	28.8 (18.6 – 40.5)
Females VOICE > 3 only	$43.0 (40.0 – 45.0)	27.0% (20.0 – 34.0)	$8.9 (8.5 – 9.3)	54.4% (51.5 – 57.4)	$6.0 (4.7 – 7.2)	31.0 (19.0 – 40.0)
Females VOICE > 3, and male clients of FSWs	$41.0 (39.0 – 43.0)	31.0% (26.0 – 36.0)	$6.4 (6.1 – 6.8)	56.5% (53.4 – 59.1)	$5.3 (4.3 – 6.3)	33.0 (24.0 – 42.0)
Females VOICE > 3, and males >1 partner	$41.0 (39.0 – 42.0)	35.0% (30.0 – 39.0)	$5.4 (5.2 – 5.7)	63.4% (60.9 – 66.0)	$5.3 (4.5 – 6.2)	40.0 (30.0 – 51.0)
Females VOICE > 1 only	$33.0 (32.0 – 35.0)	35.8% (30.2 – 41.2)	$6.4 (6.1 – 6.7)	63.0% (60.0 – 66.0)	$4.3 (3.2 – 5.5)	35.0% (26.0 – 44.0)
Females VOICE > 1, and male clients of FSWs	$33.0 (32.0 – 34.0)	39.8% (35.1 – 43.5)	$5.0 (4.7 – 5.2)	65.0% (63.0 – 67.0)	$3.9 (3.0 – 4.7)	38.0% (28.0 – 48.0)
Females VOICE > 1, and males >1 partner	$32.0 (31.0 – 33.0)	42.5% (38.3 – 46.9)	$4.2 (4.0 – 4.4)	71.0% (69.0 – 73.0)	$4.0 (3.3 – 4.9)	45.0% (37.0 – 53.0)

Figure S3: Five-Year Budget Impact for higher lenacapavir coverage scenario: Western Kenya

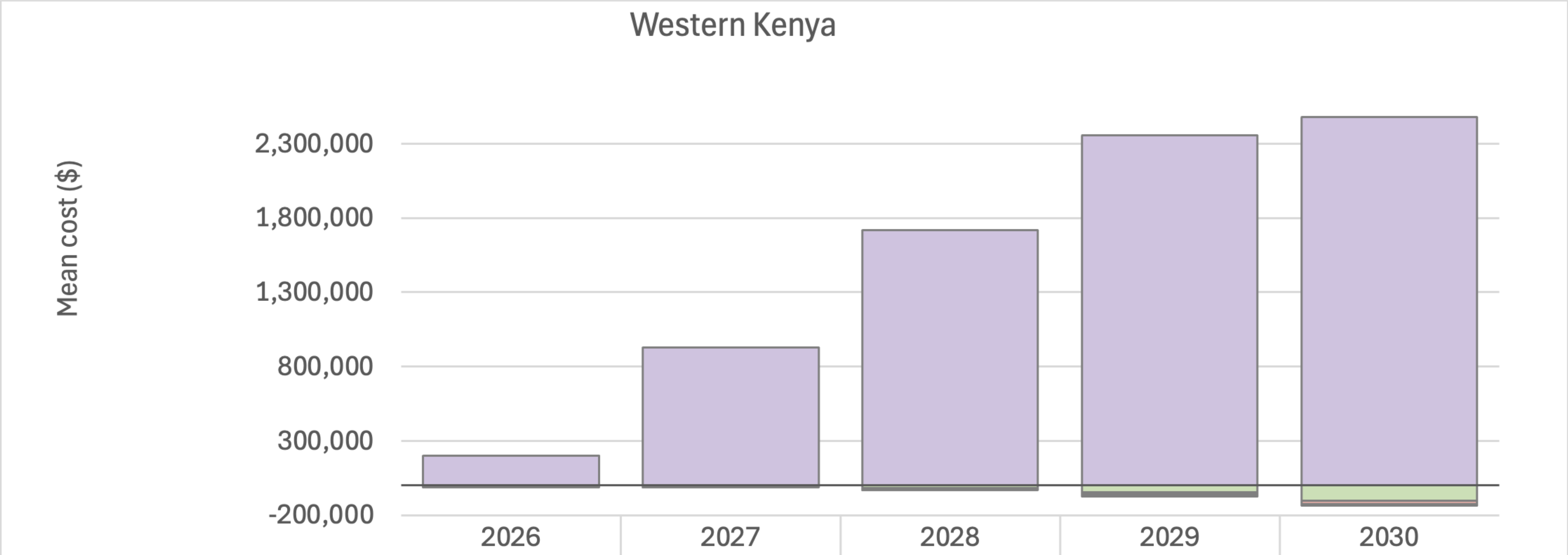

	2026	2027	2028	2029	2030
HIV testing	-276	-982	-2,663	-4,644	-741
HIV related illness	-1,640	-2,426	-5,042	-17,905	-24,998
Oral PrEP	-73	-378	-801	-1,876	-1,745
LEN	197,904	929,592	1,716,222	2,358,613	2,480,094
ART	172	-1,554	-15,046	-46,814	-101,109

Figure S4: Five-Year Budget Impact for higher lenacapavir coverage scenario: South Africa

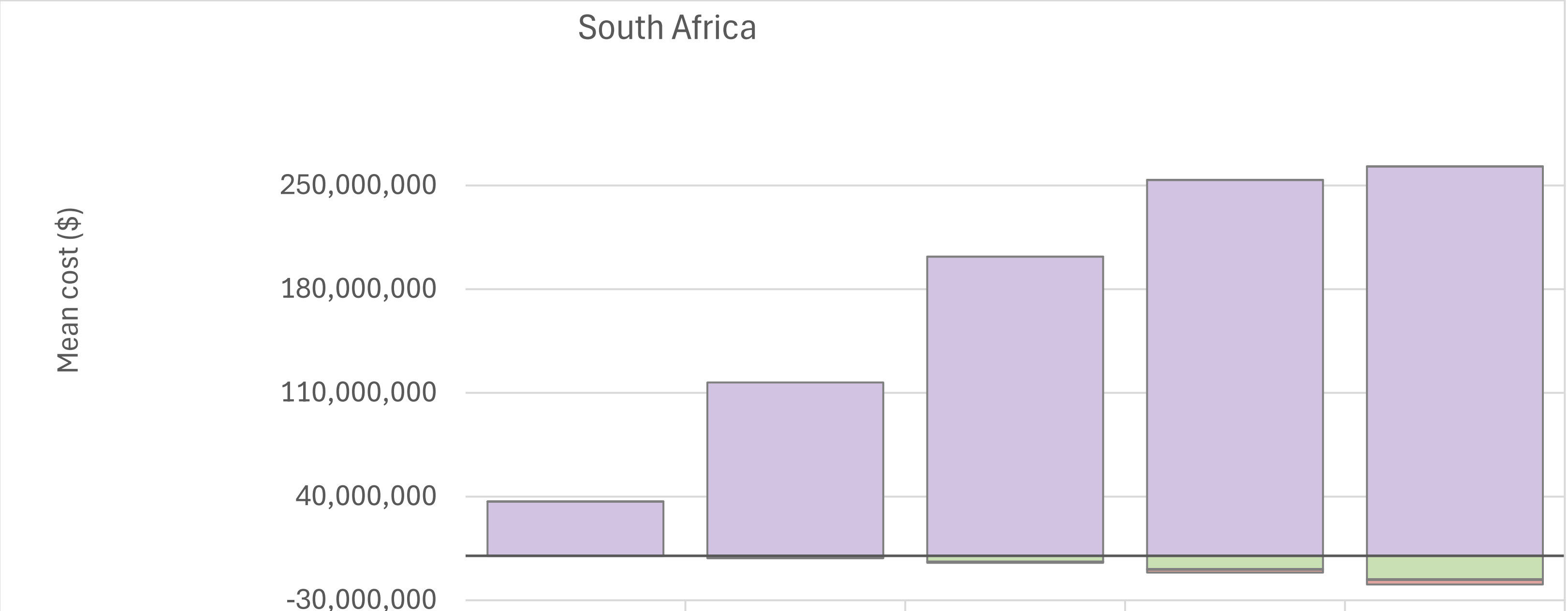

	2026	2027	2028	2029	2030
HIV testing	20,903	-84,211	49,630	80,671	205,673
HIV related illness	22,609	-337,786	-418,045	-1,928,912	-2,999,234
Oral PrEP	-44,793	-271,039	-414,574	-524,453	-563,109
LEN	36,590,444	117,063,847	201,866,378	253,669,880	262,730,099
ART	11,887	-876,205	-3,853,749	-8,908,284	-15,795,203

Figure S5: Five-Year Budget Impact for higher lenacapavir coverage scenario: Zimbabwe

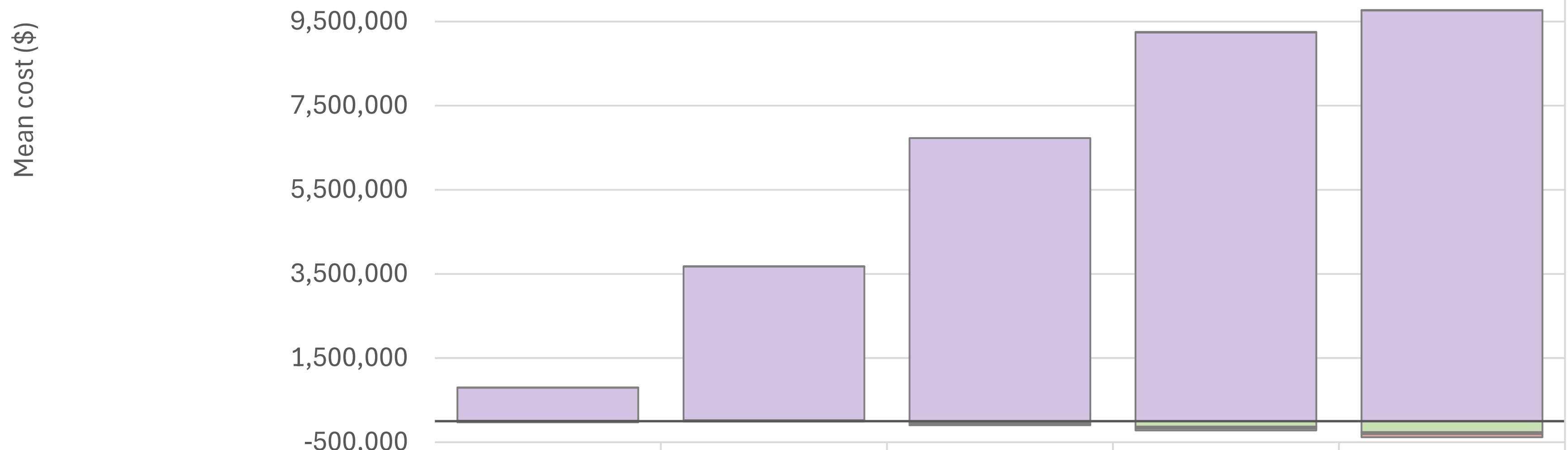

	2026	2027	2028	2029	2030
HIV testing	6,147	1,028	-3,515	14,891	9,144
HIV related illness	-20,767	-497	-31,523	-49,393	-76,460
Oral PrEP	-5,568	-7,473	-26,595	-42,257	-46,159
LEN	784,594	3,653,027	6,727,725	9,239,225	9,764,000
ART	10,112	24,421	-33,796	-129,656	-260,280

Figure S6: Component Costs by Scenario in western Kenya

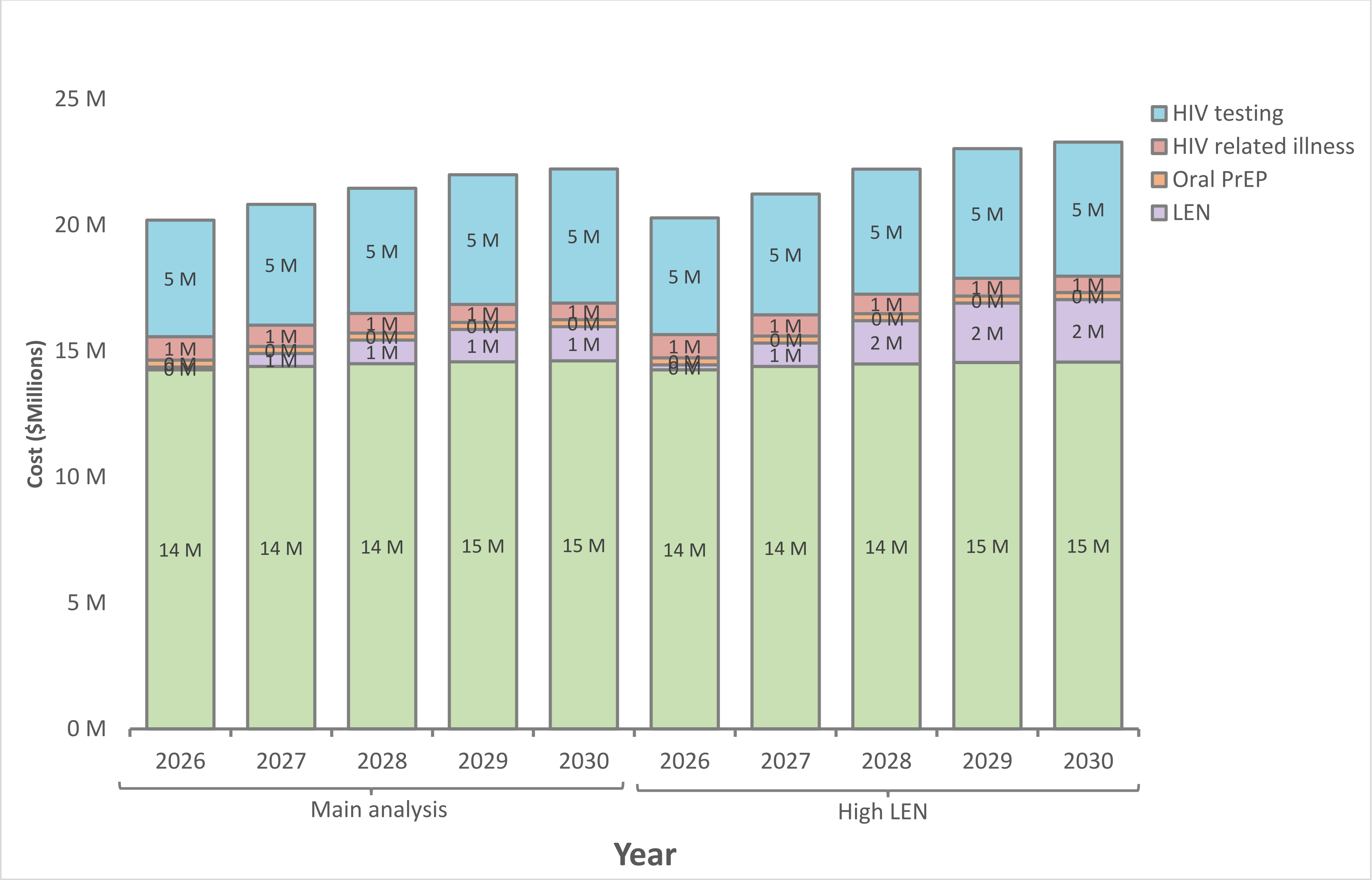

Figure S7: Component Costs by Scenario in western Kenya

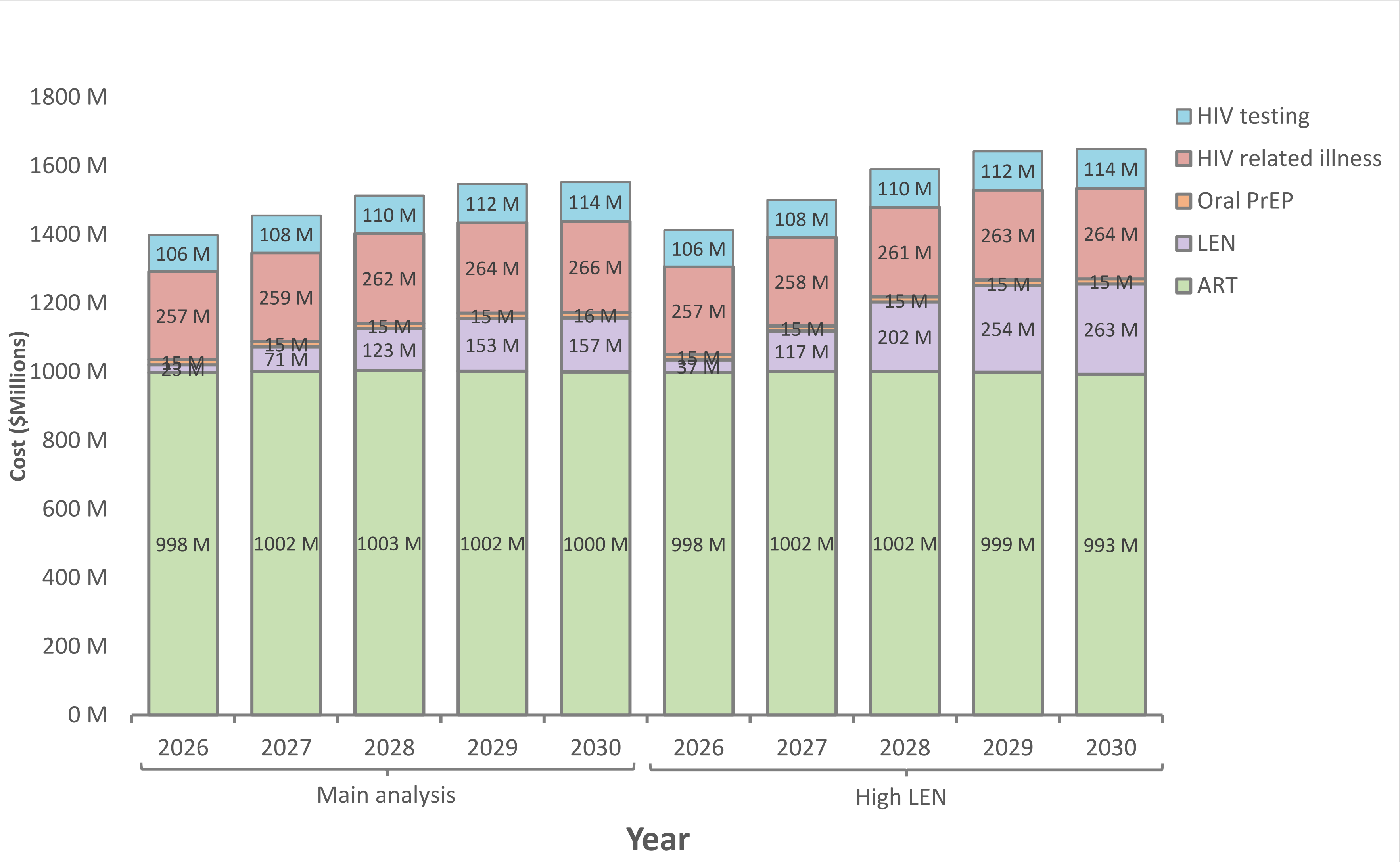

Figure S8: Component Costs by Scenario in Zimbabwe

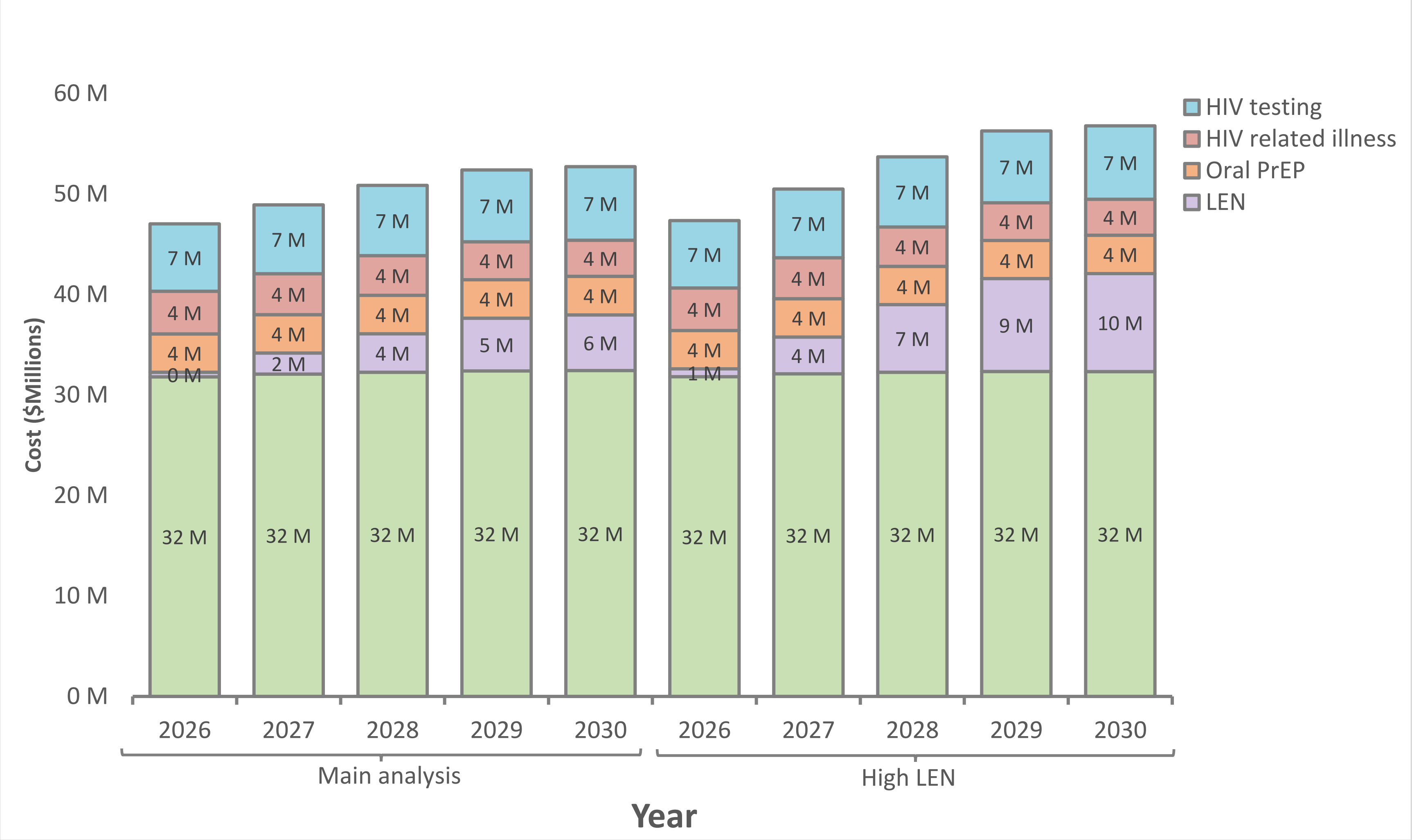

Figure S9 Tornado diagram of one-way sensitivity analyses of price threshold in three countries

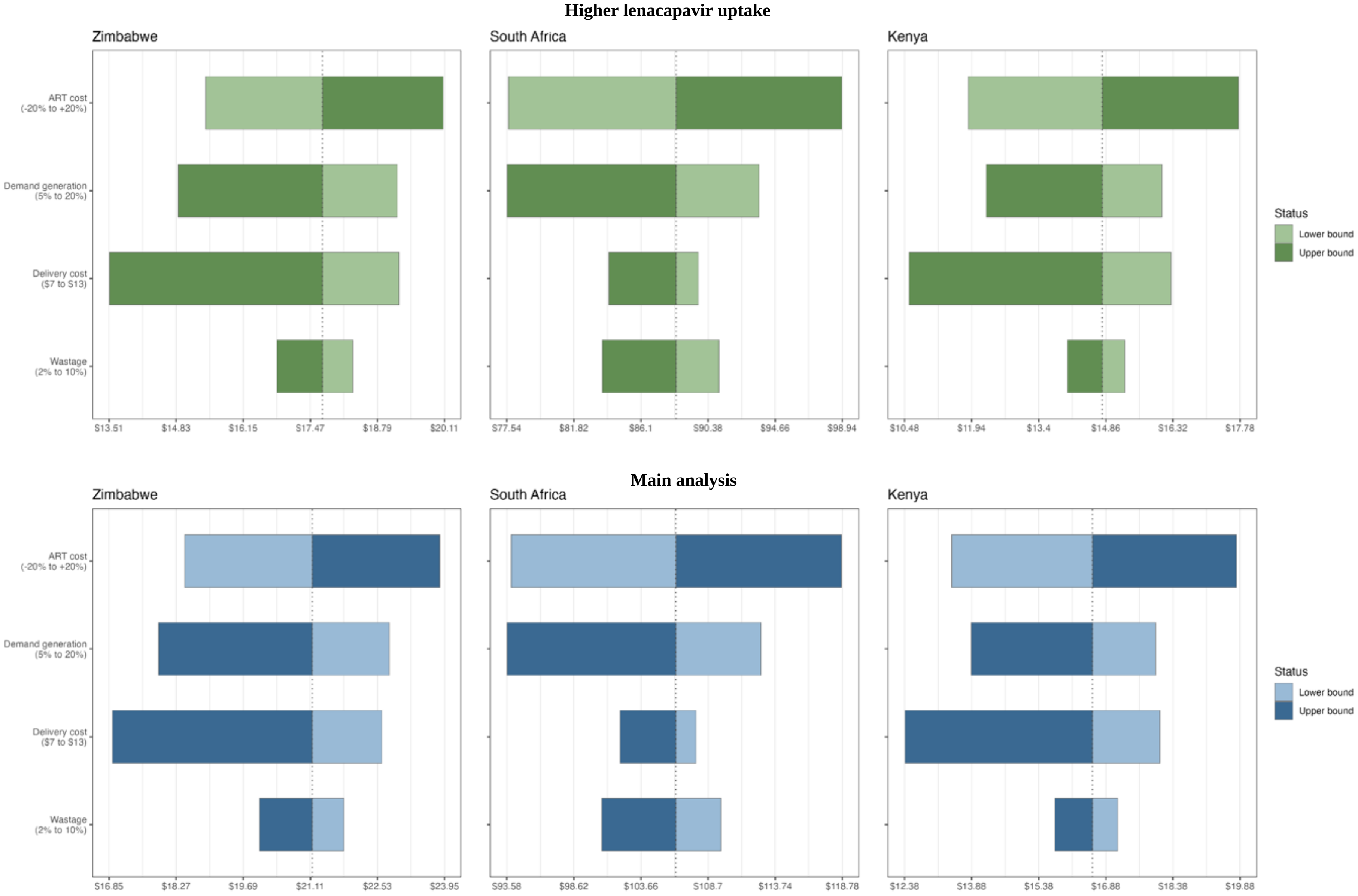

Supplement: Supplementary appendix [file mmc1.pdf]
